# An intramolecular inverse electron demand Diels–Alder approach to annulated α-carbolines

**DOI:** 10.3762/bjoc.8.93

**Published:** 2012-06-06

**Authors:** Zhiyuan Ma, Feng Ni, Grace H C Woo, Sie-Mun Lo, Philip M Roveto, Scott E Schaus, John K Snyder

**Affiliations:** 1 Department of Chemistry and the Center for Chemical Methodology and Library Development (CMLD-BU), Boston University, 590 Commonwealth Avenue, Boston, Massachusetts 02215

**Keywords:** α-carboline, chemical diversity, inverse electron demand Diels–Alder, isatin, pyrido[2,3-*b*]indole, 1,2,4-triazine

## Abstract

Intramolecular inverse electron demand cycloadditions of isatin-derived 1,2,4-triazines with acetylenic dienophiles tethered by amidations or transesterifications proceed in excellent yields to produce lactam- or lactone-fused α-carbolines. Beginning with various isatins and alkynyl dienophiles, a pilot-scale library of eighty-eight α-carbolines was prepared by using this robust methodology for biological evaluation.

## Introduction

**Figure 1:**
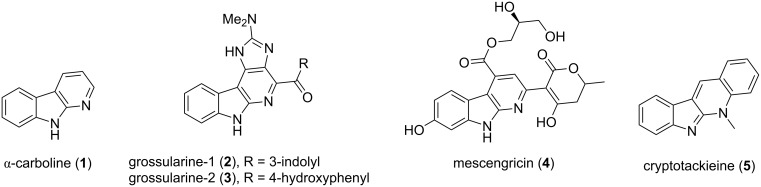
Natural products with α-carboline subunits.

In comparison with the well-known β-carbolines, α-carboline alkaloids are quite rare, and only a few natural products isolated to date contain this pyrido[2,3-*b*]indole (α-carboline, **1**, Figure 1) core. The most prominent examples are grossularine-1 (**2**) and grossularine-2 (**3**), which are marine cytotoxic agents that were isolated from the tunicate *Dendrodoa grossularia* (Stylidae) [1–2], and desmethylgrossularine-1 from tunicate *Polycarpa aurata* [3]. Other natural α-carbolines include mescengricin (**3**), an inhibitor of L-glutamate excitotoxicity in neutrons, isolated from *Streptomyces griseoflavus* [4], and cryptotackieine (**4**) [5], also known as neocryptolepine [6], isolated from roots of the West African plant *Cryptolepis sanguinolenta* [7]. Cryptotackieine, a member of the indolo[2,3-*b*]quinoline class of heterocycles [8], has been shown to be a strong inhibitor of *Plasmodium falciparum* growth [9]. 2-Amino-α-carbolines have also been identified as mutagens produced in the pyrolysis of proteins [10–11] as well as the pyrolysis of tryptophan [12]. Isoeudistomin U, isolated from the ascidian *Lissoclinum fragile*, was originally reported to have an α-carboline skeleton [13], but this assignment was later shown to be incorrect [14].

Given their isomeric relationship to β-carbolines, α-carbolines have, unsurprisingly, attracted attention from synthetic chemists for a long time [15], and more recently from medicinal chemists [16]. Synthetic members of this class were shown to have a wide range of activities, including topoisomerase II inhibition [17], and 4-amino-α-carbolines have also been shown to possess anxiolytic properties by stabilization of the open chloride channel [18–19]. Relatively few patents have been granted on the medicinal use of α-carbolines, particularly in comparison to β-carbolines, with recent applications including use as antiviral agents [20], inhibitors of ApoB-100-associated lipoprotein production for cholesterol lowering [21], and more recently, as inhibitors of CDK1 kinase as potential anticancer agents [22]. This later filing has triggered investigations into α-carbolines as potential multikinase inhibitors [23].

Existing synthetic approaches to α-carbolines are numerous and often independent of a specific target, but are instead methodology oriented. Exceptions include those efforts to target the grossularines [24–30] and the more popular cryptotackieine [31–35].

The first reported synthesis of an α-carboline by Robinson in 1924 [15,36] proceeded through the acid-catalyzed decomposition of 1-(2-pyridyl)benzotriazole, a modification of the Graebe–Ullmann carbazole synthesis, which closes the indole ring. This procedure was improved upon and exploited for decades [37–40]. Later, reversing the roles of the benzene and pyridine rings, with the former as the nucleophile and latter as electrophile through the diazonium salt, led to improved yields in the indole ring closure [41], as did microwave promotion of the original methodology [42]. Nitrene insertion chemistry of appropriately substituted 3-arylpyridines also found application [43–44], and likewise falls into the category of indole ring closure onto an existing substituted pyridine, though in this case with the formation of the C–N bond. More recently, the group of Cuny has reported two strategies that exploit cross-couplings to prepare anilinopyridines, with final indole ring closure occurring by either a second cross-coupling [45] or a photocyclization [46]. The former, sequential cross-coupling strategy closely follows the previous work by Queguiner [47]. A Fischer indole synthesis route through the 2-pyridylhydrazone of cyclohexanone, catalyzed by PPA, followed by dehydrogenation over Pd–C has also been reported for the preparation of the unsubstituted α-carboline [48], but no other successful applications of this strategy have appeared in the literature.

A more common strategy to α-carbolines closes the pyridine ring onto an existing indole framework. In general, applications of this construction sequence fall into two major camps, namely condensations routed through a 2-aminoindole synthon [49–56], and those targeting pyridine closures onto a 2-oxindole [57–58]. In addition, intramolecular Hartwig–Buchwald cross-coupling onto a 2-bromoindole, with formation of the pyridine was reported by Dodd [59], and C–N bond closures in presumed nitrogen-radical processes have also been reported by Narasaka [60–61]. Electrocyclic ring closures forming the pyridine ring from 2-aminoindole-derived intermediates are also known [62–64]. In addition, cycloadditions of 3-vinyl-7-azaindole have also been used to close the pyridine ring [65], as have intramolecular dipolar cycloadditions of 2-azidoindole derivatives [66], and intramolecular cycloadditions of carbodiimides [67–68].

**Scheme 1:**
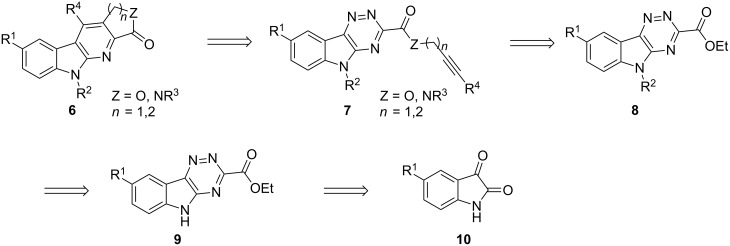
Retrosynthetic inverse electron Diels–Alder approach to α-carbolines.

We had envisioned that a library of annulated α-carboline structures **6** could be prepared by the intramolecular inverse electron demand Diels–Alder reaction (IEDDA) of isatin-derived 1,2,4-triazines **7** with tethered electron-rich dienophiles (Scheme 1) [69]. Inverse electron demand Diels–Alder cycloadditions employing electron-deficient heteroaromatic azadienes are well-established for the synthesis of heterocyclic compounds, and have been previously applied to the synthesis of α-carbolines. Intramolecular IEDDA chemistry to prepare α-carbolines employing 2-*N*-(*o*-alkynylanilino)pyrimidine systems have been successful in a limited number of examples [70–71]. Hoornaert and co-workers have also employed pyrazinones as dienes in IEDDA reactions to prepare α-carbolines [72].

Though the existing synthetic routes can readily produce various α-carbolines, the intramolecular IEDDA approach outlined in Scheme 1 has several advantages from the perspective of library synthesis. First, easy access exists to a wide selection of commercially available or easily synthesized starting materials, i.e., isatins, propargylic and homopropargylic amines and alcohols. Second, three easily modifiable diversification sites, R^1^, R^2^ and R^4^, can be built into the core α-carboline structure. Furthermore, construction of the annulated lactam or lactone as the fourth ring would be easily accomplished by the IEDDA reactions, thereby adding two more diversification points in the form of R^3^ and the lactam/lactone ring size. A particular goal in this work was to establish the reaction chemistry and scope in order to build a library of α-carbolines for biological screening. The targeted α-carbolines **6** (X = O) are similar to those prepared by Dodd by a rather lengthy route, but with a transposed carbonyl group on the lactone ring [59].

One consideration in this design was the electron donation from the indole nitrogen into the isatin-derived triazine ring of **9**, which would result in an elevated LUMO of the triazinyl azadiene, and thereby inhibit the desired cycloaddition. Thus, it was anticipated that an electron-withdrawing group R^2^, which could also serve as a diversification point, would be needed on the indole nitrogen.

## Results and Discussion

**Scheme 2:**
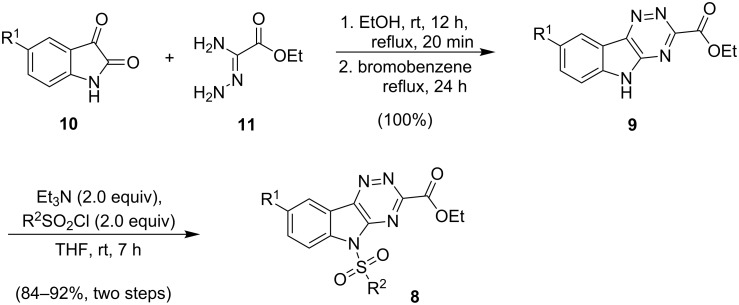
Condensation of isatins with ethyl oxaloamidrazonate to form triazines.

Feasibility studies began with isatin-derived 1,2,4-triazine **9a** (**9**, R^1^ = H) [73–74], which was easily prepared by the condensation of isatin (**10**, R^1^= H) with ethyl oxaloamidrazonate [75–77] (**11**) in quantitative yield (Scheme 2). The first step in the cyclocondensation was accomplished by stirring in ethanol at rt for 12 h and heating under reflux for 20 min, after which the solvent was removed and the cyclocondensation completed by heating under reflux in bromobenzene (bp 156 °C) for 24 h. The two-step condensation with different solvents was needed to optimize the triazine formation. Sulfonylation of the indole nitrogen also proceeded routinely to give triazine **8a** (R^1^ = H, R^2^ = *p*-Tol), and served two purposes. As noted, the reduction of electron donation from this nitrogen into the triazine ring was thought to be important for the subsequent cycloaddition to proceed, as was shown to be correct in later studies. Furthermore, sulfonylation greatly improved the solubility of the triazines **8** in organic solvents in comparison to **9**, which showed only limited solubility in dichloromethane, chloroform, THF, toluene, methanol and acetone. Starting with other 5-substituted isatin derivatives, analogous triazines were similarly prepared in good yields (84–92%, Table 1). 5-Nitro- and 5-carboxamidoisatins also readily participated in the cyclocondensation with the oxaloamidrazonate **11** to form the corresponding triazines (**9**, R^1^ = NO_2_, CONH_2_), but due to the electron withdrawing nature of the isatin substituents, subsequent sulfonylations were not successful (not shown).

**Table 1:** Preparation of N-protected isatin-derived 1,2,4-triazines **8**.^a^

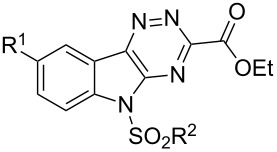				

R^2^	R^1^ = H	R^1^ = Me	R^1^ = MeO	R^1^ = F

*p*-MePh	**8a**, 88%	**8b**, 92%	**8c**, 89%	**8d**, 85%
*p*-MeOPh	**8e**, 90%	**8f**, 87%	**8g**, 84%	**8h**, 88%
Ph	**8i**, 90%	**8j**, 88%	**8k**, 87%	**8l**, 91%

^a^Isolated yield over two steps (Scheme 2).

The intermolecular IEDDA reactions of **8a** with various electron-rich dienophiles, including ethyl ethynyl ether, the enamines (*E*)-1-*N*-propenylpyrrolidine, 1-pyrrolidino-1-cyclohexene, and 2-(dimethylamino)imidazole [78–80], all of which have been shown to react quite well with triazines or tetrazines in intermolecular IEDDA cycloaditions, were investigated. However, **8a** showed no reactivity with any of these dienophiles under any conditions, further confirming the importance of the proposed intramolecular strategy for preparing α-carbolines.

**Scheme 3:**
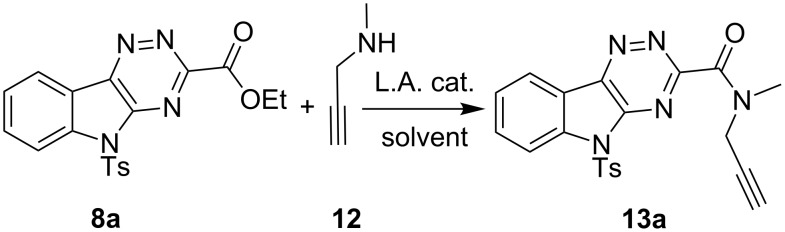
Amidation of triazine ester **8a**.

Lewis acid catalyzed amidation of **8a** with methyl propargyl amine (**12**) gave the cycloaddition precursor **13a** in excellent yield when Al(Me)_3_ was used as catalyst (Scheme 3, Table 2) [81–84]. However, the most convenient procedure for library protocols employed 1.2 equiv of Zr(O*t*-Bu)_4_ [85]. Weinreb amidation with Al(Me)_3_ also gave excellent yields, but required the extra step of first mixing Al(Me)_3_ and the amine, then cannulating this amine–AlMe_3_ complex into the triazine solution in order to avoid ketonization of the ester [86]. Other Lewis acids, MgCl_2_, Mg(OTf)_2_, Zn(OTf)_2_, Yb(OTf)_3_ and Sc(OTf)_3_, were not successful in catalyzing the amidation. Stoichiometric amounts of catalyst were required for the amidation, presumably due to the product itself sequestering the catalyst and preventing efficient turnover.

**Table 2:** Lewis acid catalysis of amidation of triazine **8a**.^a^

Entry	Catalyst	Loading (equiv)	Solvent	Yield (%)

1^b^	Al(Me)_3_	1.5	DCM	>95
2	Zr(O*t*-Bu)_4_	0.5	toluene	60
3	Zr(O*t*-Bu)_4_	1.2	toluene	90
4^c^	Zr(O*t*-Bu)_4_	1.2	DCM	>95

^a^All reactions were carried out at rt for 20 h unless otherwise noted; isolated yield. ^b^Mix catalyst and amine, then transfer the mixture of the amine–aluminum complex into a triazine solution by cannula. ^c^Reaction time was 16 h.

**Scheme 4:**
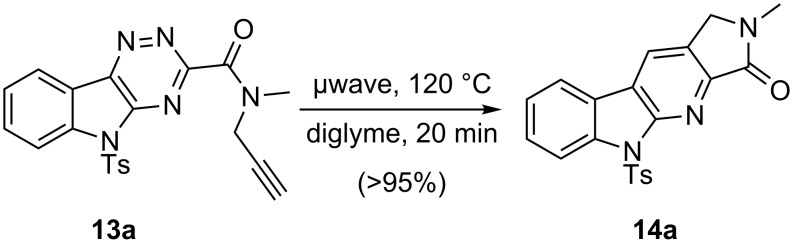
Microwave-promoted IEDDA reaction of isatin derived triazines.

The intramolecular cycloaddition of **13a**, the alkyne-tethered triazine, was studied under various conditions (Scheme 4, Table 3). Ultimately, it was found that the IEDDA reaction proceeded smoothly under microwave irradiation, in diglyme (120 °C, 20 min; Table 3, entry 3) to give the γ-lactam annulated α-carboline **14a** in quantitative yield. The microwave reaction conditions were preferred over the more traditional heating (Table 3, entry 1) due to the shorter reaction time. Attempts to lower the temperature and/or shorten the reaction time led to lower yields (Table 3, entries 2, 4 and 5). Little reaction occurred in toluene under microwave irradiation (Table 3, entry 6) unless silicon carbide chips were added as a microwave facilitator (Table 3, entry 7) [87].

**Table 3:** IEDDA reaction conditions of **13a** to give cycloadduct **14a**.^a^

Entry	Solvent	Temperature (°C)	Time	Yield

1^b^	diglyme	164	7 h	>95%
2	diglyme	100	20 min	40%
3	diglyme	120	20 min	>95%
4	diglyme	120	10 min	70%
5	diglyme	160	10 min	85%
6	toluene	160	30 min	trace
7^c^	toluene/Si–C	160	30 min	60%

^a^IEDDA reactions were carried out under microwave irradiation unless otherwise noted. All yields were determined by UPLC unless otherwise noted. ^b^Heating in oil bath. ^c^Si–C chip was added to the reaction under microwave irradiation. Isolated yield.

**Scheme 5:**
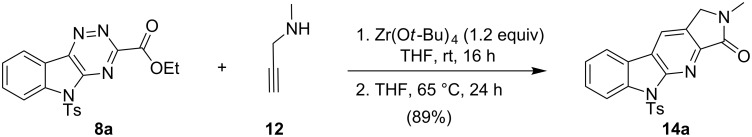
One-pot amidation/cycloaddition of triazine ester **8a**.

Upon further exploration of the reaction conditions, it was discovered that the amidation/cycloaddition sequence could be accomplished in one pot (Scheme 5). The Zr(IV)-catalyzed amidation was thus accomplished in tetrahydrofuran (16 h, rt), then the reaction mixture was heated under reflux at 65 °C for another 24 h to yield cycloadduct **14a**. This one-pot procedure gave a yield (89%) that was comparable to the overall yield (94%) of the corresponding two-step sequence, with isolation of the intermediate amide. Control experiments were carried out to determine the possible catalytic role of Zr(O*t*-Bu)_4_ in the cycloaddition. In two side-by-side reactions, **13a**, with and without Zr(O*t*-Bu)_4_ catalyst, was heated to 65 °C. The two reactions required the same amount of time (24 h) to achieve complete conversions, indicating that the presence of amidation catalyst Zr(O*t*-Bu)_4_ in the one-pot two-step sequence had no effect on the cycloaddition of **13a**. For the library synthesis, the two-step sequence was adopted in order to isolate the triazine intermediates **13**, which can also serve as library members.

**Scheme 6:**
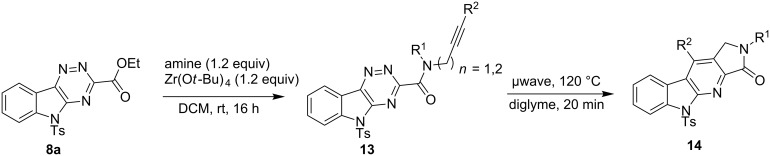
Amidation/cycloaddition forming α-carbolines **14**.

With the two-step sequence optimized for the preparation of **14a**, the scope of this chemistry was then probed with other alkynyl amines. The amidations all proceeded in high yields under the optimized conditions with Zr(O*t*-Bu)_4_ as catalyst. The cycloaddition precursors were then subjected to the optimized microwave-promoted cycloadditions to give the final cycloadducts **14** (Scheme 6 and Table 4).

**Table 4:** Amidation-IEDDA sequence to α-carbolines **14** from triazine **8a**.^a^

Entry	Amine	Cycloaddition precursor **13** (yield)	Cycloadduct **14** (yield)^b^

1	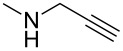	**13a** (98%)	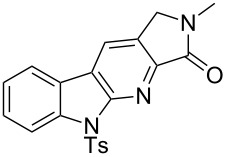 **14a** (96%)
2^c^	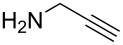	**13b** (90%)	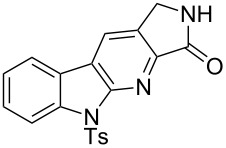 **14b** (83%)
3^d^	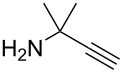	**13c** (89%)	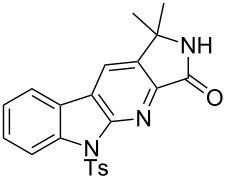 **14c** (91%)
4	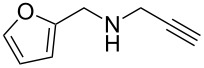	**13d** (96%)	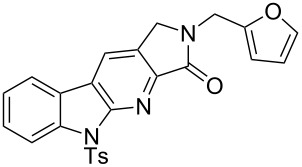 **14d** (98%)
5	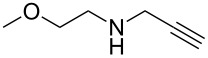	**13e** (96%)	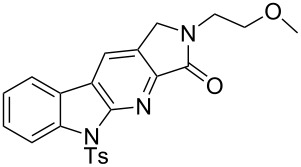 **14e** (97%)
6^e^	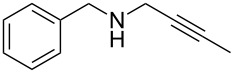	**13f** (95%)	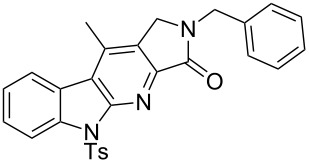 **14f** (93%)
7^f^	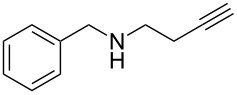	**13g** (93%)	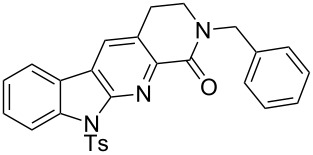 **14g** (95%)
8	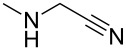	**13h** (95%)	no reaction

^a^Amidation reactions were carried out in DCM, with 1.2 equiv of amine and 1.2 equiv of Zr(O*t*-Bu)_4_ at rt for 16 h, 0.25 M. IEDDA reactions were carried out in diglyme under microwave irradiation at 120 °C for 20 min, unless otherwise noted. All IEDDA reaction concentrations were 0.1 M. ^b^Isolated yields. ^c^IEDDA in DMF at 160 °C for 30 min. ^d^IEDDA in diglyme at 160 °C for 30 min. ^e^IEDDA in diglyme at 120 °C for 40 min, 0.1 M. ^f^IEDDA in diglyme at 160 °C for 30 min.

Tertiary propargyl amides **13a**, **13d**, and **13e** with terminal alkyne dienophiles all showed comparable reactivity (Table 4, entry 1, 4, and 5), under the optimized cycloaddition conditions (diglyme, microwave irradiation, 120 °C, 20 min), producing the desired α-carbolines in excellent yields (96–98%). However, propargyl amide **13f** with the internal alkyne (Table 4, entry 6) required a longer reaction time (40 min) for the cycloaddition to be completed to good yield (93%). Increasing the tether length between the alkyne group and the amide linkage also decreased the reactivity (Table 4, entry 7). The cycloaddition of tertiary propargyl amide **13g** to produce the δ-lactam **14g** required higher temperatures (160 °C versus 120 °C) to proceed in high yield, still in 30 min.

**Scheme 7:**
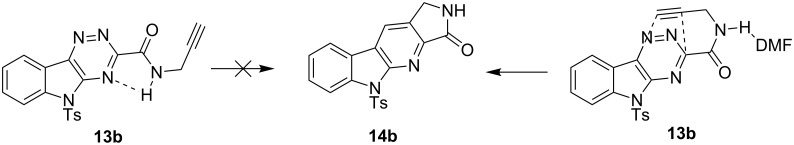
Intramolecular hydrogen bonding prevents IEDDA cycloaddition of **14b**.

In contrast to the tertiary amides, the secondary amides **13b** and **13c** (Table 4, entry 2 and 3) showed no or very little reaction under the standard microwave conditions. The reason for this lack of reactivity was thought to be strong intramolecular hydrogen bonding, which inhibits the rotation of the alkyne group to the proper position for the cycloaddition to occur (Scheme 7).

The NMR spectra also supported this hypothesis: While all tertiary amides showed the presence of two rotamers in a 1:1 ratio in CDCl_3_, the secondary amides **13b** and **13c** showed only a single rotamer in the NMR spectra. In the ^1^H NMR spectrum for **13b**, a very slowly exchanging proton (48 h for complete exchange) at δ 8.254 supported the presence of a single conformation with strong intramolecular hydrogen bonding. When the cycloaddition of **13b** was run in DMF instead of diglyme (160 °C, 20 min), the cycloadditions proceeded in good yield to give the desired cycloadduct **14b** (83%; Table 4, entry 3). Presumably the significantly greater capabilities of DMF to accept hydrogen bonds in comparison to diglyme help to disrupt the intramolecular hydrogen bonding and enable the cycloaddition to proceed. When **13c**, with the gem dimethyl substituted propargylic carbon, was run in diglyme at a higher temperature (160 °C, 30 min), the cycloaddition proceeded in good yield to give the desired product **14c** (91%; Table 4, entry 3), presumably aided by the Thorpe–Ingold effect [88–90].

**Scheme 8:**
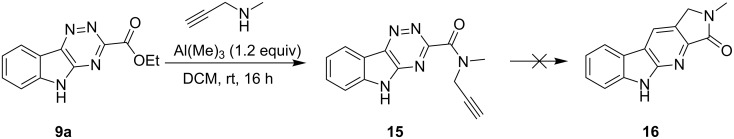
Preparation of unprotected triazine **15**, and its lack of reactivity in cycloadditions.

To confirm the importance of the electron-withdrawing group on the original indole nitrogen position, the reactivity of amide **9a** (R^1^ = H) was also examined (Scheme 8). As expected, no cycloaddition was observed under any conditions, presumably due to the greater electron donation from the indole nitrogen, which elevates the LUMO of the triazine ring, thereby preventing the desired cycloaddition.

**Scheme 9:**
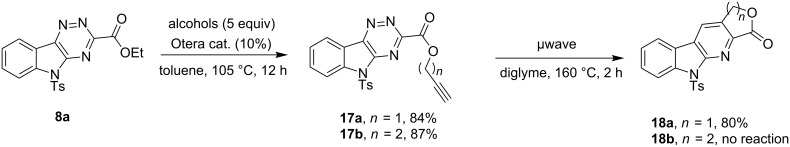
Transesterification and subsequent cycloaddition of **17a**.

In addition to the amide linkage of the alkyne dienophiles, transesterification of **8a** with alkynyl alcohols led to tethered alkynyl esters as cycloaddition precursors **17a** and **17b** in good yields (Scheme 9). Various catalysts for the transesterification reaction were screened. Boronic acid [91] and indium(III) iodide [92] yielded no transesterification product with propargyl alcohol, while Ti(OiPr)_4_ [93] gave only a trace of the desired product, with mostly detosylation resulting. Otera’s catalyst [94] proved to be optimal, giving cycloaddition precursors **17a** and **17b** in excellent yields (84% and 87%, respectively).

Cycloaddition precursors **17a** and **17b** showed a much lower reactivity in the cycloadditions in comparison to the amides **13**. For ester **17a**, after microwave irradiation at 160 °C for 2 h, the desired cycloadduct **17a** with the annulated γ-lactone was produced in good yield (80%). In contrast, **17b**, with the longer tether (*n* = 2) failed to produce the desired δ-lactone under any conditions. A sharp decrease in reactivity in intramolecular IEDDA reactions of alkynes tethered to 1,2,4-triazines upon progression from 5- to 6-membered ring annulations was previously noted by Taylor [95–98], and has been ascribed to the greater entropy loss for the larger rings [99]. Indeed, it has been estimated that the effective molarity for 5-membered ring closures can be as high as 1,000-fold greater in comparison to 6-membered ring cyclizations [100].

Using this optimized two-step amidation-IEDDA reaction-sequence methodology, an 88-membered pilot-scale library of α-carbolines was prepared by using triazine analogues **8** and alkynyl amines (Table 5). The crude library was analyzed by UPLC. The average yield of the library was 94%, with 90% of the library members produced in yields greater than 85%. Purification by LC–MS produced the final library.

**Table 5:** Library synthesis matrix.^a^

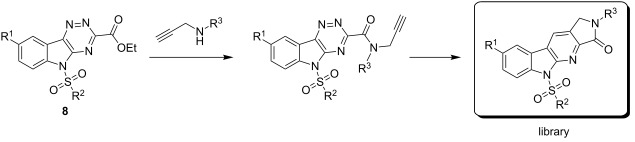												

R^3^ ↓	R^2^ (*p-*) →	R^1^ = H			R^1^ = Me			R^1^ = OMe		R^1^ = F		

		MeOPh	MePh	Ph	MePh	MeOPh	Ph	MeOPh	Ph	MeOPh	MePh	Ph

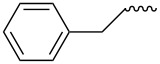		100	100	100	50	100	50	90	72	100	100	85
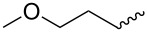		100	94^b^	100	100	100	66	100	100	99	100	100
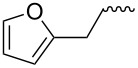		97	94^b^	100	77	98	90	100	100	100	98	100
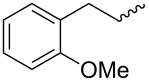		40	100	97	98	90	85	95	97	80	93	96
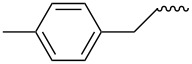		100	100	100	100	100	95	100	100	100	100	100
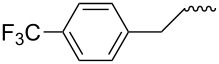		100	100	100	100	100	100	100	100	100	100	100
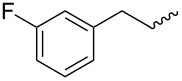		65	100	95	100	100	92	100	100	100	100	100
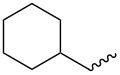		–^c^	100	100	100	–^c^	56	–^c^	–^c^	94	100	–^c^

^a^UPLC yields against starting materials. ^b^Isolated yields. ^c^Members were not prepared due to the limited amount of cyclohexyl propargylamine.

## Conclusion

Isatin-derived 1,2,4-triazines have proven to be excellent heteroaromatic azadienes for intramolecular inverse electron demand Diels–Alder reactions with tethered alkynyl dienophiles. These cycloadditions led to lactam-annulated α-carbolines in excellent yields under microwave assistance. The scope of the chemistry was probed by using various alkynyl amines and alkynyl alcohols, and by variation of the tether length between the aminoalkyne and the triazine. The triazines with ester linkages showed significantly less reactivity in cycloadditions compared to those with amide linkages. The longer tether length also led to a decrease in cycloaddition reactivity. This chemistry was subsequently applied to a library synthesis, producing a focused library of eighty-eight members. Diversity was introduced by using a combination of various substituted isatin-derived triazines, with various sulfonylations of the indole nitrogen, and propargyl amine derivatives as dienophiles.

## Experimental

**General procedure A, preparation of isatin-derived triazines 9:** Freshly prepared ethyl oxalamidrazonate (**11**) [76] was dissolved in anhydrous EtOH (0.1 M) and the isatin (1.0 equiv) was added at rt under stirring. The reaction mixture was stirred at rt for 12 h, and then heated under reflux for 20 min. After removal of the EtOH in vacuo, the residue was dissolved in anhydrous bromobenzene (0.2 M) and refluxed for 24 h. After removal of the solvent in vacuo, the residue was dried by the addition and evaporation of toluene three times, and used directly in the next step without any further purification.

**General procedure B, preparation of sulfonamides 8:** The isatin-derived triazine **9** was suspended in THF (0.25 M) and triethylamine (2.0 equiv) was added to the solution at rt. The reaction mixture was stirred at rt for 30 min until dissolution was completed, then the sulfonyl chloride (2.0 equiv) was added at rt, and the reaction mixture was stirred at rt for 7 h. After removal of the solvent in vacuo, the residue was purified by flash chromatography to yield the desired sulfonylated triazine **8**.

**Representative example:** Ethyl 5-[(4-methylbenzene)sulfonyl]-5*H*-[1,2,4]triazino[5,6-*b*]indole-3-carboxylate (**8a**). According to general procedures A and B, beginning with isatin (350 mg, 2.38 mmol, 1.0 equiv) and *p*-toluenesulfonyl chloride (907 mg, 4.76 mmol, 2.0 equiv). Purification by flash chromatography, *R*_f_ 0.68 (hexanes/EtOAc, 1:1) gave **8a** (827 mg, 2.09 mmol, 88% yield) as a yellow solid: mp 145–146 °C; IR (neat): 2982, 1742, 1379, 1191, 1180, 671, 579 cm^−1^; ^1^H NMR (400 MHz, CDCl_3_) δ 1.54 (t, *J* = 7.0 Hz, 3H), 2.36 (s, 3H), 4.60 (q, *J* = 7.0 Hz, 2H), 7.29 (d, *J* = 8.4 Hz, 2H), 7.58 (ddd, *J* = 8.0, 7.4, 0.8 Hz, 1H), 7.83 (ddd, *J* = 8.6, 7.4, 1.0 Hz, 1H), 8.20 (d, *J* = 8.4 Hz, 2H), 8.49 (ddd, *J* = 8.0, 0.8, 0.8 Hz, 1H), 8.52 (ddd, *J* = 8.6, 1.0, 0.8 Hz, 1H); ^13^C NMR (100 MHz, CDCl_3_) δ 14.4, 21.9, 63.1, 115.1, 118.9, 123.4, 125.8, 128.8 (2C), 130.1 (2C), 133.7, 134.4, 140.5, 145.0, 146.8, 146.9, 152.6, 162.7; HRMS–ESI (*m*/*z*): [M + Na]^+^ calcd for C_19_H_16_N_4_O_4_SNa, 419.0790; found, 419.0770 (100%).

**General procedure C, amidation of 8 to amides 13:** To a solution of triazine **8** (1.0 equiv) and propargyl amine (1.2 equiv) in CH_2_Cl_2_ (0.25 M) was added Zr(O*t*-Bu)_4_ (1.2 equiv) at rt. The reaction mixture was stirred at rt for 16 h, then the mixture was diluted with CH_2_Cl_2_ (2 mL) and passed through an HM/HCl plug (hydro-matrix plug saturated with 0.1 M HCl aqueous solution) eluting with CH_2_Cl_2_. The filtrate was dried over sodium sulfate, and then the solvent was removed in vacuo. The residue was purified by flash chromatography to yield the desired amide **13**.

**Representative example:*****N*-Methyl-5-[(4-methylbenzene)sulfonyl]-*N*-(prop-2-yn-1-yl)-5*H*-[1,2,4]triazino[5,6-*b*]indole-3-carboxamide (**13a**). According to general procedure C, beginning with **8a** (80 mg, 0.20 mmol, 1.0 equiv) and *N*-methyl-propargylamine (16.7 mg, 0.24 mmol, 1.2 equiv). Purification by flash chromatography, *R*_f_ 0.54 (DCM/EtOAc, 10:1, 83 mg, 0.19 mmol, 98% yield) gave **13a** as a brown-orange oil: IR (neat): 2933, 1655, 1370, 1192, 1178, 732, 666 cm^−1^; ^1^H NMR (400 MHz, CDCl_3_) δ 2.31 (t, *J* = 2.4 Hz, 0.37H, minor rotamer), 2.34–2.36 (overlap, 3.63H), 3.12 (s, 1.89H, major rotamer), 3.31 (s, 1.11H, minor rotamer), 4.22 (d, *J* = 2.4 Hz, 0.74H, minor rotamer), 4.52 (d, *J* = 2.4 Hz, 1.26H, major rotamer), 7.29 (d, *J* = 8.6 Hz, 2H), 7.57 (dd, *J* = 8.0, 8.0 Hz, 1H), 7.81 (dd, *J* = 8.2, 8.0 Hz, 1H), 8.11 (d, *J* = 8.6 Hz, 1.24H, major rotamer), 8.12 (d, *J* = 8.6 Hz, 0.74H, minor rotamer), 8.44 (d, *J* = 8.0 Hz, 1H), 8.49 (d, *J* = 8.2 Hz, 1H); ^13^C NMR (75 MHz, CDCl_3_) δ 21.9, (33.2, 36.0, 1C), (36.6, 40.9, 1C), (73.1, 73.9, 1C), (77.4, 77.9, 1C), 115.0, 119.1, 123.0, 125.8, 128.3 (2C), 130.3 (2C), 133.3, 134.4, 139.9, 144.3, 146.8, 147.0, (156.6, 156.8, 1C), 164.6; HRMS–ESI (*m*/*z*): [M + Na]^+^ calculated for C_21_H_17_N_5_O_3_SNa, 442.0950; found, 442.0935 (100%).

**General procedure D, cycloaddition of 13 to 14:** A solution of the amide **13** in diglyme (0.1 M) was placed in a thick-walled microwave tube, and then the reaction mixture was subjected to microwave irradiation at 160 °C for 20 min under stirring, unless otherwise noted. After the irradiation, the solvent was removed in vacuo and the residue was purified by flash chromatography to yield the cycloadducts **14**.

**Representative example:** 13-Methyl-8-[(4-methylbenzene)sulfonyl]8,10,13-triazatetracyclo[7.7.0.0^2,7^.0^11,15^]hexadeca-1(16),2(7),3,5,9,11(15)-hexaen-12-one (**14a**). According to general procedure D, beginning with **13a** (30 mg, 0.071 mmol). Purification by flash chromatography, *R*_f_ 0.17 (DCM:EtOAc, 2:1, 27 mg, 0.069 mmol, 96% yield) gave product **14a** as a white yellow solid: mp 271–272 °C; IR (neat): 3057, 2987, 1739, 1692, 1375, 1175, 667 cm^−1^; ^1^H NMR (400 MHz, CDCl_3_) δ 2.28 (s, 3H), 3.26 (s, 3H), 4.40 (s, 2H), 7.20 (d, *J* = 8.4 Hz, 2H), 7.32 (dd, *J* = 8.0, 7.8 Hz, 1H), 7.51 (dd, *J* = 7.8, 7.8 Hz, 1H), 7.86 (d, *J* = 7.8 Hz, 1H), 8.13 (s, 1H), 8.20 (d, *J* = 8.4 Hz, 2H), 8.42 (d, *J* = 8.0 Hz, 1H); ^13^C NMR (100 MHz, CDCl_3_) δ 21.8, 30.4, 49.7, 115.0, 120.3, 121.1, 121.7, 122.8, 123.9, 128.6 (2C), 129.3, 129.6 (2C), 130.6, 135.5, 138.8, 145.4, 147.7, 151.3, 166.3; HRMS–ESI (*m*/*z*) [M + Na]^+^ calcd for C_21_H_17_N_3_O_3_SNa, 414.0888; found, 414.0873 (100%).

## Supporting Information

File 1 Experimental details and characterization data of new compounds, ^1^H NMR and ^13^C NMR spectra.
