# Supplementary material for: An intramolecular inverse electron demand Diels–Alder approach to annulated α-carbolines
Source: Beilstein J Org Chem. 2012 Jun 6;8:829–40. doi: 10.3762/bjoc.8.93 (PMC3388871; doi:10.3762/bjoc.8.93)
Supplement: File 1 — Experimental details and characterization data of new compounds, 1H NMR and 13C NMR spectra. [file Beilstein_J_Org_Chem-08-829-s001.pdf]

Supporting Information

for

An intramolecular inverse electron demand Diels–Alder route to α-carbolines

Zhiyuan Ma, Feng Ni, Grace H. C. Woo, Sie-Mun Lo, Philip M. Roveto, Scott E. Schaus, John K. Snyder*

Address: Department of Chemistry and the Center for Chemical Methodology and Library Development (CMLD-BU), Boston University, 590 Commonwealth Avenue, Boston, Massachusetts 02215

Email: John K. Snyder - jsnyder@bu.edu

* Corresponding author

Experimental details and characterization data of new compounds, ^{1}H NMR and ^{13}C NMR spectra

Table of Contents

1. General methods S2
2. General procedures S3–S4
3. Compound procedures and characterization data S5–S23
4. References S24
5. ^{1}H NMR and ^{13}C NMR spectra S25–S86

General methods. Melting points were determined on a capillary melting point apparatus and are uncorrected. The ^{1}H NMR and ^{13}C NMR spectra were recorded at 117.42 kG (^{1}H 500 MHz, ^{13}C 125 MHz), 93.94 kG (^{1}H 400 MHz, ^{13}C 100 MHz), or 70.50 kG (^{1}H 300 MHz, ^{13}C 75 MHz) at ambient temperature as noted. Hydrogen chemical shifts are expressed in parts per million (ppm) relative to the residual protio solvent resonance: $CDCl_3$ δ 7.24, DMSO δ 2.50. For ^{13}C spectra, the centerline of the solvent signal was used as an internal reference: $CDCl_3$ δ 77.16, DMSO δ 39.52. Unless otherwise noted, each carbon resonance represents a single carbon (relative intensity). Inverse gated decoupled spectra using 10 s delays between transients were used for carbon resonance integration to establish relative intensities greater than 1 carbon when not obvious. Infrared spectra were recorded on an FTIR spectrometer by depositing one drop of a sample solution in a volatile solvent (typically $CHCl_3$) directly onto the reflective plate, followed by evaporation of the solvent. For infrared spectra, only diagnostic bands (including, but not limited to: OH, carbonyl and SO_2 stretching frequencies) are reported. High-resolution mass spectrometric data were obtained on a QToF (hybrid quadrupolar/time-of-flight) API US system by electrospray (ESI) in the positive-ion mode. Mass correction was done by an external reference using a lockspray accessory. Mobile phases were water and acetonitrile (1:9) with 0.1% formic acid with a flow rate of 0.2 mL/min. The MS settings were: capillary voltage 3 kV, cone voltage 45, source temperature 120 °C and desolvation temperature 350 °C. All reactions were carried out under an argon atmosphere unless otherwise noted. All microwave reactions were run in sealed 10 mL thick-walled microwave pressure tubes in a microwave reactor. Microwave reaction temperatures were measured by using a volume-independent infrared temperature sensor located within the microwave cavity. An "HM/HCl plug" refers to a Hydromatrix SPE column saturated with 1.0 M HCl water solution. Flash chromatography was

performed on silica gel-60 (43–60 μm). Tetrahydrofuran (THF) was distilled from sodium/benzophenone and dichloromethane (CH_2Cl_2) was distilled from calcium hydride immediately prior to use. Ethanol and methanol were distilled from magnesium and iodine immediately prior to use. Bromobenzene was dried over sodium sulfate prior to use. Triethylamine ($C_6H_{15}N$) was distilled from potassium hydroxide. All isatins are commercially available and were used without further purification. Other commercially available starting materials were used without further purification unless otherwise noted.

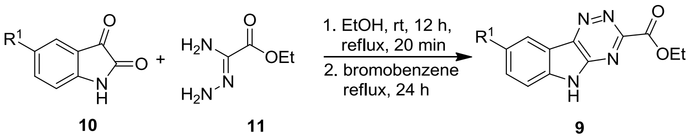

General procedure A, preparation of isatin-derived triazines 9: Freshly prepared ethyl oxalamidrazonate (**11**) [1] was dissolved in anhydrous EtOH (0.1 M), and the isatin (1.0 equiv) was added at rt under stirring. The reaction mixture was stirred at rt for 12 h, and then heated under reflux for 2 h. After removal of the EtOH in vacuo, the residue was dissolved in anhydrous bromobenzene (0.2 M) and heated under reflux for 24 h. After removal of the solvent in vacuo, the residue was dried by addition and evaporation of toluene three times, and used directly for the next step without any further purification.

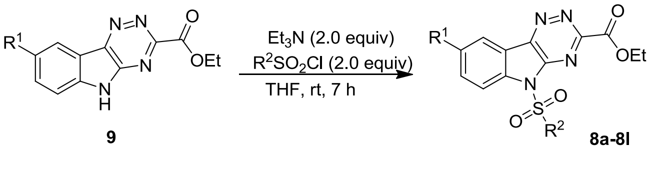

General procedure B, preparation of sulfonamides 8: The isatin-derived triazine **9** was suspended in THF (0.25 M) and triethylamine (2.0 equiv) was added into the solution at rt. The reaction mixture was stirred at rt for 30 min until dissolution was completed, then *p*-toluenesulfonyl chloride (2.0 equiv) was added at rt, and the reaction mixture was stirred at rt for 7 h. After removal of the solvent in vacuo, the residue was purified by flash chromatography to yield the desired triazine **8**.

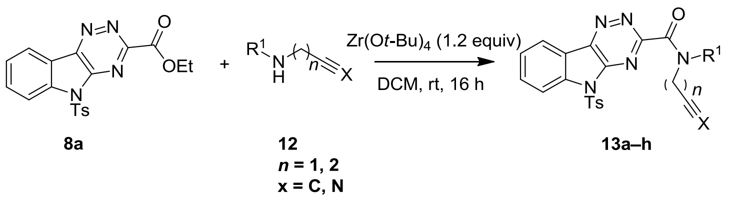

General procedure C, amidation of 8a to amides 13: To a solution of triazine **8a** (1.0 equiv) and propargyl amine (1.2 equiv) in CH_2Cl_2 (0.25 M) was added Zr(O*t*-Bu)$_4$ (1.2 equiv) at rt. The reaction mixture was stirred at rt for 16, then the mixture was diluted with CH_2Cl_2 (2 mL) and passed through an HM/HCl plug (hydro-matrix plug saturated with 0.1 M HCl aqueous solution) eluting with CH_2Cl_2. The filtrate was dried over sodium sulfate, and then the solvent was removed in vacuo. The residue was purified by flash chromatography to yield the desired amide **13**.

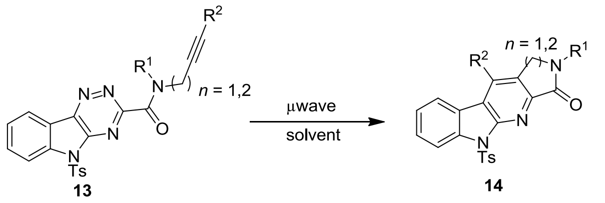

General procedure D, cycloaddition of 13 to 14: A solution of the amide **13** in diglyme (0.1 M) was placed in a thick-walled microwave tube, and then the reaction mixture was subjected to microwave irradiation at 160 °C for 20 min under stirring unless otherwise noted. After the irradiation the solvent was removed in vacuo and the residue was purified by flash chromatography to yield the cycloadducts **14**.

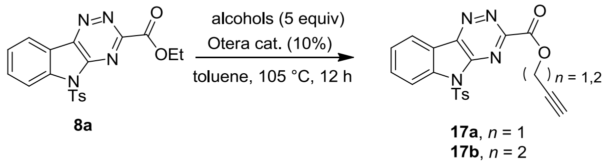

General procedure E, transesterification of 8 to 17 using Otera's catalyst. To a solution of triazine carboxylate (1.0 equiv) and the Otera's catalyst (0.1 equiv) [2] in toluene (0.1 M) was added the alkynyl alcohol (excess, greater than 5 equiv) at rt. The reaction mixture was heated and stirred at 105 °C for 12 hours. Then the reaction was filtered through Celite, and the solvent and excess alcohol were removed in vacuo. The residue was purified by chromatography to yield the desired esters.

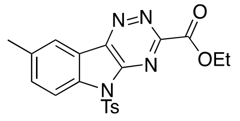

8b

Ethyl 8-methyl-5-[(4-methylbenzene)sulfonyl]-5*H*-[1,2,4]triazino[5,6-*b*]indole-3-carboxylate (8b). Prepared according to general procedures A and B, beginning with ethyl oxalamidrazonate (**11**, 243 mg, 1.86 mmol, 1.0 equiv), 5-methylisatin (300 mg, 1.86 mmol, 1.0 equiv) and 4-methylbenzenesulfonyl chloride (710 mg, 3.72 mmol, 2.0 equiv). Purification by flash chromatography (DCM/EtOAc, 10:1, R_f 0.70) gave **8b** (700 mg, 1.71 mmol, 92% yield) as a yellow solid: mp 164–165 °C; IR (neat) 2984, 2925, 1743, 1192, 1179, 579 cm^{-1}; ^{1}H NMR (400 MHz, $CDCl_3$) δ 1.53 (t, *J* = 7.2 Hz, 3H), 2.35 (s, 3H), 2.54 (s, 3H), 4.60 (q, *J* = 7.2 Hz, 2H), 7.27 (d, *J* = 8.4 Hz, 2H), 7.63 (dd, *J* = 8.6, 0.9 Hz, 1H), 8.16 (d, *J* = 8.4 Hz, 2H), 8.28 (d, *J* = 0.9 Hz, 1H), 8.38 (d, *J* = 8.6 Hz, 1H); ^{13}C NMR (100 MHz, $CDCl_3$) δ 14.4, 21.4, 21.9, 63.1, 114.8, 119.0, 123.2, 128.7 (2C), 130.1 (2C), 134.4, 135.0, 136.1, 138.7, 145.0, 146.8, 146.9, 152.5, 162.8; HRMS (ESI) *m*/*z* 433.0939 ([M + Na]$^+$, 100%), calcd for $C_{20}H_{18}N_4O_4SNa$ 433.0939.

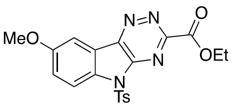

8c

Ethyl 8-methoxy-5-[(4-methylbenzene)sulfonyl]-5*H*-[1,2,4]triazino-[5,6-*b*]-indole-3-carboxylate (8c). Prepared according to general procedures A and B, beginning with ethyl oxalamidrazonate (**11**, 306 mg, 2.34 mmol, 1.0 equiv), 5-methoxyisatin (415 mg, 2.34 mmol, 1.0 equiv) and 4-methylbenzenesulfonyl chloride (894 mg, 4.69 mmol, 2.0 equiv). Purification by flash chromatography (DCM/EtOAc, 10:1, R_f 0.68) gave **8c** (890 mg, 2.09 mmol, 89% yield) as a yellow solid: mp 136–138 °C; IR (neat) 2893, 1741, 1493, 1180, 581 cm^{-1}; ^{1}H NMR (400 MHz, $CDCl_3$) δ 1.54 (t, *J* = 7.1 Hz, 3H), 2.35 (s, 3H), 3.94 (s, 3H), 4.61 (q, *J* = 7.1 Hz, 2H), 7.27 (d, *J* = 8.6 Hz, 2H), 7.40 (dd, *J* = 9.3, 2.2 Hz, 1H), 7.91 (d, *J* = 2.2 Hz, 1H), 8.15 (d, *J* = 8.6 Hz, 2H), 8.41 (d, *J* = 9.3 Hz, 1H); ^{13}C NMR (100 MHz, $CDCl_3$) δ 14.3, 21.8, 56.1, 63.0, 104.6, 116.2,

119.7, 122.9, 128.5 (2C), 130.0 (2C), 134.3, 134.8, 144.9, 146.7, 146.8, 152.4, 157.9, 162.6; HRMS (ESI) *m*/*z* 449.0885 ([M + Na]$^+$, 100%), calcd for $C_{20}H_{18}N_4O_5SNa$ 449.0896.

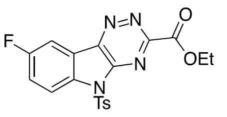
8d

Ethyl 8-fluoro-5-[(4-methylbenzene)sulfonyl]-5*H*-[1,2,4]triazino[5,6-*b*]indole-3-carboxylate (8d). Prepared according to general procedures A and B, beginning with ethyl oxalamidrazonate (**11**, 278 mg, 2.12 mmol, 1.0 equiv), 5-fluoroisatin (350 mg, 2.12 mmol, 1.0 equiv) and 4-methylbenzenesulfonyl chloride (809 mg, 4.24 mmol, 2.0 equiv). Purification by flash chromatography (hexanes/EtOAc, 1:1, R_f 0.80) gave **8d** (740 mg, 1.79 mmol, 85% yield) as a yellow solid: mp 155–157 °C; IR (neat) 2984, 1744, 1489, 1192, 1179, 579 cm^{-1}; ^{1}H NMR (400 MHz, $CDCl_3$) δ 1.54 (t, J = 7.2 Hz, 3H), 2.36 (s, 3H), 4.61 (q, J = 7.2 Hz, 2H), 7.30 (d, J = 8.2 Hz, 2H), 7.56 (ddd, J_{HF} = 9.0 Hz, J = 9.0, 2.8 Hz, 1H), 8.15 (dd, J_{HF} = 7.6 Hz, J = 2.8 Hz, 1H), 8.18 (d, J = 8.2 Hz, 2H), 8.51 (dd, J = 9.0 Hz, J_{HF} = 4.2 Hz, 1H); ^{13}C NMR (100 MHz, $CDCl_3$) δ 14.4, 21.9, 63.2, 109.4 (d, $^2J_{CF}$ = 24.4 Hz), 116.8 (d, $^3J_{CF}$ = 8.4 Hz), 120.2 (d, $^3J_{CF}$ = 9.9 Hz), 121.4 (d, $^2J_{CF}$ = 14.4 Hz), 128.7 (2C), 130.2 (2C), 134.1, 136.6, 144.6 (d, $^4J_{CF}$ = 3.8 Hz), 147.09, 147.13, 152.8, 160.4 (d, $^1J_{CF}$ = 245.4 Hz), 162.5; HRMS (ESI) *m*/*z* 437.0683 ([M + Na]$^+$, 100%), calcd for $C_{19}H_{15}FN_4O_4SNa$ 437.0696.

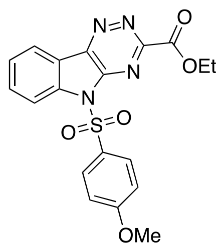
8e

Ethyl 5-[(4-methoxybenzene)sulfonyl]-5*H*-[1,2,4]triazino[5,6-*b*]indole-3-carboxylate (8e). Prepared according to general procedures A and B, beginning with ethyl oxalamidrazonate (**11**, 284 mg, 2.17 mmol, 1.0 equiv), isatin (320 mg, 2.17 mmol, 1.0 equiv) and 4-methoxybenzenesulfonyl chloride (899 mg, 4.35 mmol, 2.0 equiv). Purification by flash chromatography (hexanes/EtOAc, 1:1, R_f 0.62) gave **8e** (810 mg, 1.96

mmol, 90% yield) as a yellow solid: mp 185–187 °C; IR (neat) 2983, 1742, 1593, 1380, 1190, 1174, 579 cm^{-1}; ^{1}H NMR (400 MHz, $CDCl_3$) δ 1.53 (t, *J* = 7.1 Hz, 3H), 3.80 (s, 3H), 4.60 (q, *J* = 7.1 Hz, 2H), 6.94 (d, *J* = 8.8 Hz, 2H), 7.58 (dd, *J* = 7.8, 7.6 Hz, 1H), 7.83 (ddd, *J* = 8.4, 7.6, 1.2 Hz, 1H), 8.26 (d, *J* = 8.8 Hz, 2H), 8.49–8.53 (overlap, 2H); ^{13}C NMR (100 MHz, $CDCl_3$) δ 14.4, 55.9, 63.1, 114.7 (2C), 115.1, 118.9, 123.4, 125.7, 128.6, 131.3 (2C), 133.6, 140.6, 145.0, 146.7, 152.6, 162.7, 165.1; HRMS (ESI) *m*/*z* 435.0743 ($[M + Na]^+$, 100%), calcd for $C_{19}H_{16}N_4O_5SNa$ 435.0739.

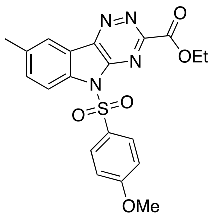

8f

Ethyl 5-[(4-methoxybenzene)sulfonyl]-8-methyl-5*H*-[1,2,4]triazino-[5,6-*b*]indole-3-carboxylate (8f). Prepared according to general procedures A and B, beginning with ethyl oxalamidrazonate (**11**, 261 mg, 1.99 mmol, 1.0 equiv), 5-methylisatin (320 mg, 1.99 mmol, 1.0 equiv) and 4-methoxybenzenesulfonyl chloride (821 mg, 3.97 mmol, 2.0 equiv). Purification by flash chromatography (DCM/EtOAc, 10:1, R_f 0.71) gave **8f** (730 mg, 1.71 mmol, 87% yield) as a yellow solid: mp 173–174 °C; IR (neat) 2892, 1741, 1192, 1167, 670 cm^{-1}; ^{1}H NMR (400 MHz, $CDCl_3$) δ 1.53 (t, *J* = 7.1 Hz, 3H), 2.55 (s, 3H), 3.80 (s, 3H), 4.61 (q, *J* = 7.1 Hz, 2H), 6.93 (d, *J* = 9.0 Hz, 2H), 7.64 (d, *J* = 8.6 Hz, 1H), 8.24 (d, *J* = 9.0 Hz, 2H), 8.30 (s, 1H), 8.39 (d, *J* = 8.6 Hz, 1H); ^{13}C NMR (100 MHz, $CDCl_3$) δ 14.4, 21.3, 55.9, 63.1, 114.6 (2C), 114.8, 118.9, 123.2, 128.6, 131.2 (2C), 134.9, 135.9, 138.7, 145.0, 146.8, 152.4, 162.8, 165.1; HRMS (ESI) *m*/*z* 449.0880 ($[M + Na]^+$, 100%), calcd for $C_{20}H_{18}N_4O_5SNa$ 449.0896.

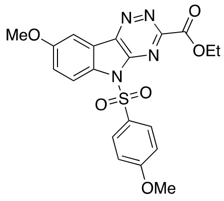

8g

Ethyl 8-methoxy-5-[(4-methoxybenzene)sulfonyl]-5*H*-[1,2,4]triazino-[5,6-*b*]indole-3-carboxylate (8g). Prepared according to general procedures A and B, beginning with ethyl oxalamidrazonate (**11**, 296 mg, 2.26 mmol, 1.0 equiv), 5-methoxyisatin (400 mg, 2.26 mmol, 1.0 equiv) and 4-methoxybenzenesulfonyl chloride (934 mg, 4.52 mmol, 2.0 equiv). Purification by flash chromatography (DCM/EtOAc, 10:1, R_f 0.65) gave **8g** (835 mg, 1.89 mmol, 84% yield) as a yellow solid: mp 163–165 °C; IR (neat) 2893, 1742, 1593, 1495, 1191, 1170, 579, 554 cm^{-1}; ^1H NMR (400 MHz, $CDCl_3$) δ 1.53 (t, *J* = 7.2 Hz, 3H), 3.80 (s, 3H), 3.94 (s, 3H), 4.60 (q, *J* = 7.2 Hz, 2H), 6.92 (d, *J* = 9.0 Hz, 2H), 7.39 (dd, *J* = 9.2, 2.8 Hz, 1H), 7.90 (d, *J* = 2.8 Hz, 1H), 8.22 (d, *J* = 9.0 Hz, 2H), 8.40 (d, *J* = 9.2 Hz, 1H); ^{13}C NMR (100 MHz, $CDCl_3$) δ 14.3, 55.8, 56.0, 63.0, 104.6, 114.5 (2C), 116.1, 119.6, 122.7, 128.4, 131.0 (2C), 134.7, 144.8, 146.6, 152.3, 157.7, 162.7, 164.9; HRMS (ESI) *m/z* 465.0848 ($[M + Na]^+$, 100%), calcd for $C_{20}H_{18}N_4O_6SNa$ 465.0845.

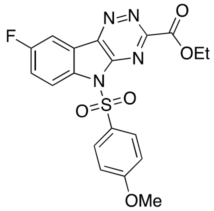

8h

Ethyl 8-fluoro-5-[(4-methoxybenzene)sulfonyl]-5*H*-[1,2,4]triazino[5,6-*b*]indole-3-carboxylate (8h). Prepared according to general procedures A and B, beginning with ethyl oxalamidrazonate (**11**, 278 mg, 2.12 mmol, 1.0 equiv), 5-fluoroisatin (350 mg, 2.12 mmol, 1.0 equiv) and 4-methoxybenzenesulfonyl chloride (876 mg, 4.24 mmol, 2.0 equiv). Purification by flash chromatography (hexanes/EtOAc, 1:1, R_f 0.72) gave **8h (**795 mg, 1.85 mmol, 88% yield) as a yellow solid: mp 165–167 °C; IR (neat) 2984, 1743, 1593, 1489, 1267, 1193, 1170, 1155, 579 cm^{-1}; ^1H NMR (400 MHz, $CDCl_3$) δ 1.52 (t, *J* = 7.2 Hz, 3H), 3.79 (s, 3H), 4.59 (q, *J* = 7.2 Hz, 2H), 6.92 (d, *J* = 8.8 Hz, 2H), 7.53 (ddd, J_{HF} = 9.1 Hz, J = 8.8, 2.4 Hz, 1H), 8.10 (dd, J_{HF} = 7.2

Hz, J = 2.4 Hz, 1H), 8.21 (d, J = 8.8 Hz, 1H), 8.48 (dd, J = 9.1 Hz, J_{HF} = 4.2 Hz, 1H); ^{13}C NMR (100 MHz, $CDCl_3$) δ 14.4, 55.9, 63.1, 109.3 (d, $^2J_{CF}$ = 24.4 Hz), 114.7 (2C), 116.7 (d, $^3J_{CF}$ = 8.4 Hz), 120.1 (d, $^3J_{CF}$ = 9.9 Hz), 121.3 (d, $^2J_{CF}$ = 24.4 Hz), 128.3, 131.2 (2C), 136.6 (d, $^4J_{CF}$ = 1.5 Hz), 144.5 (d, $^4J_{CF}$ = 3.8 Hz), 147.0, 152.7, 160.3 (d, $^1J_{CF}$ = 245.4 Hz), 162.5, 165.2; HRMS (ESI) m/z 453.0642 ([M + Na]$^+$, 100%), calcd for $C_{19}H_{15}FN_4O_5SNa$ 453.0645.

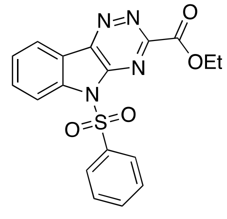

8i

Ethyl 5-(benzenesulfonyl)-5*H*-[1,2,4]triazino[5,6-*b*]indole-3-carboxylate (8i). Prepared according to general procedures A and B, beginning with ethyl oxalamidrazonate (**11**, 302 mg, 2.31 mmol, 1.0 equiv), isatin (340 mg, 2.31 mmol, 1.0 equiv) and benzenesulfonyl chloride (816 mg, 4.62 mmol, 2.0 equiv). Purification by flash chromatography (hexanes/EtOAc, 1:1, R_f 0.68) gave **8i** (795 mg, 2.08 mmol, 90% yield) as a yellow solid: mp 192–193 °C; IR (neat) 2985, 1742, 1449, 1379, 1190, 727, 588 cm^{-1}; ^{1}H NMR (400 MHz, $CDCl_3$) δ 1.53 (t, J = 7.1 Hz, 3H), 4.60 (q, J = 7.1 Hz, 2H), 7.51 (dd, J = 8.2, 7.8 Hz, 2H), 7.59 (dd, J = 7.8, 7.4 Hz, 1H), 7.63 (tt, J = 8.2, 1.2 Hz, 1H), 7.84 (ddd, J = 8.6, 7.4, 1.0 Hz, 1H), 8.32 (dd, J = 8.2, 1.2 Hz, 2H), 8.48 (dd, J = 7.8, 1.0 Hz, 2H), 8.52 (d, J = 8.6 Hz, 1H); ^{13}C NMR (100 MHz, $CDCl_3$) δ 14.4, 63.1, 115.1, 118.9, 123.5, 125.9, 128.7 (2C), 129.5 (2C), 133.7, 135.4, 137.4, 140.5, 145.1, 146.8, 152.6, 162.6; HRMS (ESI) m/z 405.0634 ([M + Na]$^+$, 100%), calcd for $C_{18}H_{14}N_4O_4SNa$ 405.0633.

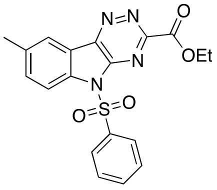

8j

Ethyl 5-(benzenesulfonyl)-8-methyl-5*H*-[1,2,4]triazino[5,6-*b*]indole-3-carboxylate (8j). Prepared according to general procedures A and B, beginning with ethyl oxalamidrazonate (**11**, 243 mg, 1.86 mmol, 1.0 equiv), 5-methylisatin (300 mg, 1.86 mmol, 1.0 equiv) and benzenesulfonyl chloride (658 mg, 3.72 mmol, 2.0 equiv). Purification by flash chromatography (DCM/EtOAc, 10:1, R_f 0.74) gave **8j** (645 mg, 1.63 mmol, 88% yield) as a yellow solid: mp 171–173 °C; IR (neat) 2985, 1743, 1193, 729, 591 cm^{-1}; ^{1}HNMR (400 MHz, $CDCl_3$) δ 1.53 (t, *J* = 7.1 Hz, 3H), 2.55 (s, 3H), 4.60 (q, *J* = 7.1 Hz, 2H), 7.50 (dd, *J* = 8.0, 7.6 Hz, 2H), 7.60–7.65 (overlap, 2H), 8.29–8.31 (overlap, 3H), 8.39 (d, *J* = 8.4 Hz, 1H); ^{13}C NMR (100 MHz, $CDCl_3$) δ 14.3, 21.2, 63.0, 114.7, 118.9, 123.1, 128.5 (2C), 129.4 (2C), 134.8, 135.2, 136.0, 137.3, 138.5, 144.9, 146.8, 152.4, 162.6; HRMS (ESI) *m*/*z* 419.0777 ([M + Na]$^+$, 100%), calcd for $C_{19}H_{16}N_4O_4SNa$ 419.0790.

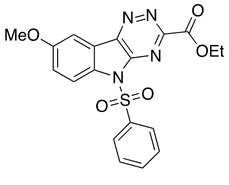

8k

Ethyl 5-(benzenesulfonyl)-8-methoxy-5*H*-[1,2,4]triazino[5,6-*b*]indole-3-carboxylate (8k). Prepared according to general procedures A and B, beginning with ethyl oxalamidrazonate (**11**, 296 mg, 2.26 mmol, 1.0 equiv), 5-methoxyisatin (400 mg, 2.26 mmol, 1.0 equiv) and benzenesulfonyl chloride (798 mg, 4.52 mmol, 2.0 equiv). Purification by flash chromatography (DCM/EtOAc, 10:1, R_f 0.65) gave **8k** (805 mg, 1.95 mmol, 87% yield) as a yellow solid: mp 154–155 °C; IR (neat) 2982, 1740, 1492, 1187, 1161, 727, 585 563 cm^{-1}; ^{1}H NMR (400 MHz, $CDCl_3$) δ 1.53 (t, *J* = 7.1 Hz, 3H), 3.93 (s, 3H), 4.60 (q, *J* = 7.1 Hz, 2H), 7.39 (dd, *J* = 9.2, 2.6 Hz, 1H), 7.49 (dd, *J* = 7.8, 7.2 Hz, 2H), 7.61 (t, *J* = 7.2 Hz 1H), 7.89 (d, *J* = 2.6 Hz, 1H), 8.28 (d, *J* = 7.8 Hz, 2H), 8.40 (d, *J* = 9.2

Hz, 1H); ^{13}C NMR (100 MHz, $CDCl_3$) δ 14.4, 56.2, 63.1, 104.8, 116.2, 119.9, 123.0, 128.6 (2C), 129.5 (2C), 134.8, 135.3, 137.4, 145.0. 146.9, 152.5, 158.0, 162.7; HRMS (ESI) *m*/*z* 435.0727 ($[M + Na]^+$, 100%), calcd for $C_{19}H_{16}N_4O_5SNa$ 435.0739.

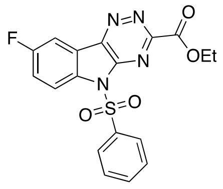

8l

Ethyl 5-(Benzenesulfonyl)-8-fluoro-5*H*-[1,2,4]triazino[5,6-*b*]indole-3-carboxylate (8l). Prepared according to general procedures A and B, beginning with ethyl oxalamidrazonate (**11**, 262 mg, 2.00 mmol, 1.0 equiv), 5-fluoroisatin (330 mg, 2.00 mmol, 1.0 equiv) and benzenesulfonyl chloride (706 mg, 4.00 mmol, 2.0 equiv). Purification by flash chromatography (hexanes/EtOAc, 1:1, R_f 0.76, 725 mg) gave **8l (**1.81 mmol, 91% yield) as a yellow solid: mp 190–192 °C; IR (neat) 2985, 1743, 1489, 1450, 1191, 1155, 585 cm^{-1}; ^{1}H NMR (400 MHz, $CDCl_3$) δ 1.53 (t, J = 7.1 Hz, 3H), 4.60 (q, J = 7.1 Hz, 2H), 7.52 (dd, J = 8.0, 7.9 Hz, 2H), 7.57 (dd, J = 9.2, 2.8 Hz, 1H), 7.64 (t, J = 7.9 Hz, 1H), 8.15 (dd, J_{HF} = 7.6, J = 2.8 Hz, 1H), 8.31 (d, J = 8.0 Hz, 2H), 8.52 (dd, J = 9.2, J_{HF} = 4.4 Hz, 1H); ^{13}C NMR (100 MHz, $CDCl_3$) δ 14.4, 63.3, 109.5 (d, $^2J_{CF}$ = 24.4 Hz), 116.7 (d, $^3J_{CF}$ = 8.4 Hz), 120.3 (d, $^3J_{CF}$ = 9.1 Hz), 121.5 (d, $^2J_{CF}$ = 25.2 Hz), 128.7 (2C), 129.6 (2C), 135.6, 136.5 (d, $^4J_{CF}$ = 1.5 Hz), 137.2, 144.7 (d, $^4J_{CF}$ = 3.9 Hz), 147.2, 152.8, 160.5 (d, $^1J_{CF}$ = 246.1 Hz), 162.5; HRMS (ESI) *m*/*z* 423.0534 ($[M + Na]^+$, 100%), calcd for $C_{18}H_{13}FN_4O_4SNa$ 423.0539.

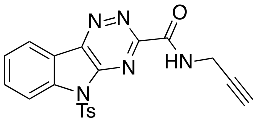

13b

5-[(4-Methylbenzene)sulfonyl]-*N*-(prop-2-yn-1-yl)-5*H*-[1,2,4]triazino[5,6-*b*]indole-3-carboxamide (13b). Prepared according to General Procedure C, beginning with **8a** (80 mg, 0.2 mmol, 1.0 equiv) and propargylamine (14 mg, 97% weight, 0.24 mmol, 1.2 equiv). Purification by flash chromatography (DCM/EtOAc, 10:1, R_f 0.43) gave **13b** (73 mg, 0.18 mmol, 90% yield) as a white-yellow solid: mp 244–245 °C; IR (neat) 3296, 2928, 1675, 1380, 1176 cm^{-1}; ^1H NMR (400 MHz, $CDCl_3$) δ 2.33 (t, *J* = 2.4 Hz, 1H), 2.36 (s, 3H), 4.39 (dd, *J* = 5.3, 2.4 Hz, 2H), 7.31 (d, *J* = 8.4 Hz, 2H), 7.60 (ddd, *J* = 7.8, 7.7, 0.8 Hz, 1H), 7.84 (ddd, *J* = 8.3, 7.7, 1.2 Hz, 1H), 8.19 (d, *J* = 8.4 Hz, 2H), 8.25 (br t, *J* = 5.3 Hz, NH), 8.49 (dd, *J* = 7.8, 1.2 Hz, 1H), 8.53 (dd, *J* = 8.3, 0.8 Hz, 1H); ^{13}C NMR (100 MHz, $CDCl_3$) δ 21.9, 29.9, 72.3, 79.0, 115.2, 118.9, 123.3, 125.9, 128.7 (2C), 130.3 (2C), 133.7, 134.5, 140.6, 145.0, 147.0, 147.2, 152.7, 160.8; HRMS (ESI) *m/z* 428.0778 ($[M + Na]^+$, 45%), calcd for $C_{20}H_{15}N_5O_3SNa$ 428.0793.

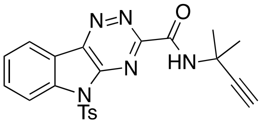

13c

5-[(4-Methylbenzene)sulfonyl]-*N*-(2-methylbut-3-yn-2-yl)-5*H*-[1,2,4]triazino[5,6-*b*]indole-3-carboxamide (13c). Prepared according to general procedure C, beginning with **8a** (80 mg, 0.2 mmol, 1.0 equiv) and 2-methyl-3-butyn-2-amine (22 mg, 95% weight, 0.24 mmol, 1.2 equiv). Purification by flash chromatography (DCM/EtOAc, 10:1, R_f 0.35) gave **13c** (77 mg, 0.18 mmol, 89% yield) as a yellow oil: IR (neat) 3387, 3293, 2981, 1700, 1513, 1376, 1193, 1179, 1087, 672 cm^{-1}; ^1H NMR (400 MHz, $CDCl_3$) δ 1.84 (s, 6H), 2.35 (s, 3H), 2.44 (s, 1H), 7.31 (d, *J* = 8.2 Hz, 2H), 7.58 (ddd, *J* = 8.0, 7.6, 0.8 Hz, 1H), 7.83 (ddd, *J* = 8.8, 7.6, 1.6 Hz, 1H), 8.22 (d, *J* = 8.2 Hz, 2H), 8.30 (br s, NH), 8.46 (br d, *J* = 8.0 Hz, 1H), 8.52 (d, *J* = 8.8 Hz, 1H); ^{13}C NMR (75 MHz,

$CDCl_3$) δ 21.8, 28.9 (2C), 48.1, 69.8, 86.6, 115.1, 118.7, 123.1, 125.6, 128.6 (2C), 130.1 (2C), 133.4, 134.4, 140.3, 144.6, 146.8, 147.3, 153.1, 159.5; HRMS (ESI) *m/z* 456.1095 ($[M + Na]^+$, 38%), calcd for $C_{22}H_{19}N_5O_3SNa$ 456.1106.

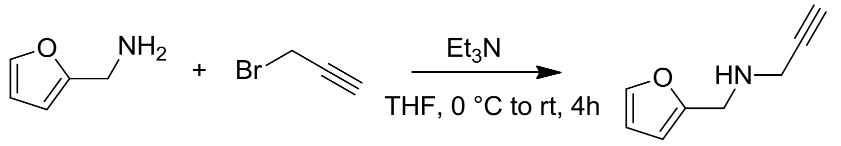

***N*-(Furan-2-ylmethyl)prop-2-yn-1-amine**. To a solution of 2-furfurylamine (1.0 g, 10.3 mmol, 1.2 equiv) and triethylamine (1.04 g, 10.30 mmol, 1.2 equiv) in THF (12 ml, 0.80 M) at 0 °C was added propargyl bromide (1.28 g, 8.58 mmol, 1.0 equiv, 80% wt in toluene) dropwise over 30 min. The reaction mixture was allowed to warm to rt and stirred for 4 h. The mixture was then filtered to remove the salt, and the solvent was then removed in vacuo. The crude residue was purified via flash chromatography to give *N*-(furan-2-ylmethyl)prop-2-yn-1-amine (DCM/EtOAc, 5:1, R_f 0.38, 0.82 g, 71% yield) as a yellow oil: IR (neat) 3293, 2921, 2836, 1148, 1102, 1010, 736 cm^{-1}; ^1H NMR (400 MHz, $CDCl_3$) δ 1.53 (br s, NH_2), *2*.21 (t, *J* = 2.4 Hz, 1H), 3.39 (d, *J* = 2.4 Hz, 2H), 3.84 (s, 2H), 6.18 (dd, *J* = 3.2, 0.8 Hz, 1H), 6.27 (dd, *J* = 3.2, 1.6 Hz, 1H), 7.33 (dd, *J* = 1.6, 0.8 Hz, 1H); ^{13}C NMR (100 MHz, $CDCl_3$) δ 37.2, 44.7, 71.8, 81.7, 107.6, 110.2, 142.1, 153.0; HRMS was not obtained.

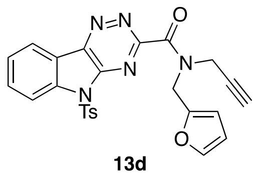

13d

***N*-(Furan-2-ylmethyl)-5-[(4-methylbenzene)sulfonyl]-*N*-(prop-2-yn-1-yl)-5*H*-[1,2,4]triazino[5,6-*b*]indole-3-carboxamide (13d).** Prepared according to general procedure C, starting with **8a** (80 mg, 0.20 mmol, 1.0 equiv) and (furan-2-ylmethyl)(prop-2-yn-1-yl)amine (41 mg, 0.30 mmol, 1.5 equiv). Purification by flash chromatography (DCM/EtOAc, 10:1, R_f 0.67) gave **13d** (94 mg, 0.19 mmol, 96% yield) as a brown-orange oil: IR (neat) 2952, 2833, 1661, 1450, 1373, 1179, 1035, 665 cm^{-1}; ^{1}H NMR (400 MHz, $CDCl_3$) δ 2.25 (t, *J* = 2.4 Hz, 0.42H, minor rotamer), 2.34 (s, 3H) 2.35 (overlap t, *J* = 2.4 Hz, 0.58H, major isomer), 4.21 (d, *J* = 2.4 Hz, 0.84H, minor rotamer), 4.48 (d, *J* = 2.4 Hz, 1.16H, major rotamer), 4.73 (s, 1.16H, major rotamer), 5.00 (s, 0.84H, minor rotamer), 6.27 (dd, *J* = 2.8, 1.2 Hz, 0.58H, major rotamer), 6.32 (d, *J* = 2.8 Hz, 0.58H, major rotamer), 6.39 (dd, *J* = 2.8, 1.2 Hz, 0.42H, minor rotamer), 6.48 (d, *J* = 2.8 Hz, 0.42H, minor rotamer), 7.27 (d, *J* = 8.2 Hz, 1.16H, major rotamer), 7.28 (d, *J* = 8.2 Hz, 0.84H, minor rotamer) 7.32 (d, *J* = 1.2 Hz, 0.58H, major rotamer), 7.45 (d, *J* = 1.2 Hz, 0.42H, minor rotamer), 7.58 (dd, *J* = 7.9, 7.8 Hz, 1H), 7.81 (dd, *J* = 8.3, 7.9 Hz, 1H), 8.11 (d, *J* = 8.2 Hz, 1.16H, major rotamer), 8.13 (d, *J* = 8.2 Hz, 0.84H, minor rotamer), 8.46 (d, *J* = 7.8 Hz, 1H), 8.50 (d, *J* = 8.3 Hz, 1H); ^{13}C NMR (125 MHz, $CDCl_3$, assumed rotamer pairs in parentheses) δ 21.9, (34.3, 38.3), (41.2, 44.6), (73.1, 73.8), (77.6, 77.8), (109.7, 109.9), 110.7, 115.1, 119.1, 123.1, 125.9, 128.4 (2C), 130.3 (2C), 133.3, 134.4, 140.0, 143.0, 144.3, 146.7, 146.9, (148.8, 149.4), (156.3, 156.6), (164.3, 164.5); HRMS (ESI) *m*/*z* 508.1034 ($[M + Na]^+$, 100%), calcd for $C_{25}H_{19}N_5O_4SNa$ 508.1055.

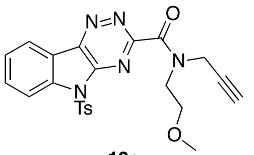

13e

***N*-(2-Methoxyethyl)-5-[(4-methylbenzene)sulfonyl]-*N*-(prop-2-yn-1-yl)-5*H*-[1,2,4]triazino[5,6-*b*]indole-3-carboxamide (13e).** Prepared according to general procedure C, beginning with **8a** (80 mg, 0.20 mmol, 1.0 equiv) and (2-methoxyethyl)(prop-2-yn-1-yl)amine [3] (35 mg, 0.30 mmol, 1.5 equiv). Purification by flash chromatography (DCM/EtOAc, 20:1, R_f = 0.32) gave **13e** (89 mg, 0.19 mmol, 96% yield) as a brown-orange oil: IR (neat) 2946, 2834, 1652, 1372, 1191, 1178, 1034, 666 cm^{-1}; ^{1}H NMR (400 MHz, $CDCl_3$) δ 2.22 (t, *J* = 2.4 Hz, 0.37H, minor rotamer), 2.33 (s, 3H), 2.34 (overlap t, *J* = 2.4 Hz, 0.63H, major rotamer), 3.27 (s, 1.89H, major rotamer), 3.42 (s, 1.11H, minor rotamer), 3.65–3.67 (overlap, 2.52H), 3.78 (t, *J* = 5.2 Hz, 0.74H, minor rotamer), 3.96 (t, *J* = 5.2 Hz, 0.74H, minor rotamer), 4.35 (d, *J* = 2.4 Hz, 0.74H, minor rotamer), 4.60 (d, *J* = 2.4 Hz, 1.26H, major rotamer), 7.277 (overlap d, *J* = 8.4 Hz, 0.74H, minor rotamer), 7.281 (overlap d, *J* = 8.4 Hz, 1.36H, major rotamer), 7.56 (dd, *J* = 7.4, 7.4 Hz, 1H), 7.78 (ddd, *J* = 8.4, 7.4, 1.2 Hz 1H), 8.10 (d, *J* = 8.4 Hz, 1.26H, major rotamer), 8.12 (d, *J* = 8.4 Hz, 0.74H, minor rotamer), 8.43 (br d, *J* = 7.4 Hz, 0.63H, major rotamer), 8.44 (br d, *J* = 7.4 Hz, 0.37H, minor rotamer), 8.47 (d, *J* = 8.4 Hz, 0.63H, major rotamer), 8.49 (d, *J* = 8.4 Hz, minor rotamer); ^{13}C NMR (75 MHz, $CDCl_3$, assumed rotamer pairs in parentheses) δ 21.8, (35.9, 40.4), (45.7, 47.9), 59.2, (71.1, 71.3), (72.8, 73.4), (78.3, 78.5), 115.0, 119.1, 123.0, 125.8, 128.3 (2C), 130.3 (2C), 133.2, 134.4, 139.9, 144.2, 146.7, 146.9, (156.7, 157.0), (164.6, 165.1); HRMS (ESI) *m*/*z* 486.1205 ([M + Na]$^+$, 100%), calcd for $C_{23}H_{21}N_5O_4SNa$ 486.1212.

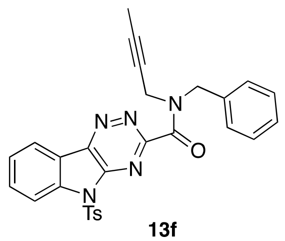

13f

***N*-Benzyl-*N*-(but-2-yn-1-yl)-5-[(4-methylbenzene)sulfonyl]-5*H*-[1,2,4]triazino[5,6-*b*]indole-3-carboxamide (13f).** Prepared according to general procedure C, beginning with **8a** (80 mg, 0.20 mmol, 1.0 equiv) and benzyl(but-2-yn-1-yl)amine [4] (49 mg, 0.30 mmol, 1.5 equiv). Purification by flash chromatography (hexane/EtOAc, 3:1, R_f = 0.19) gave **13f** (98 mg, 0.19 mmol, 95% yield) as a brown-orange oil: IR (neat) 3031, 2920, 1656, 1372, 1191, 1179, 730 cm^{-1}; ^1H NMR (400 MHz, $CDCl_3$) δ 1.74 (br s, 1.23H, minor rotamer), 1.88 (br s, 1.77H, major rotamer), 2.33 (s, 3H), 3.98 (s, 0.82H, minor rotamer), 4.32 (s, 1.18H, major rotamer), 4.60 (s, 1.18H, major rotamer), 5.01 (s, 0.82H, minor rotamer), 7.20–7.28 (overlap, 2H), 7.30–7.38 (overlap, 2H), 7.38–7.44 (overlap, major and minor rotamer, 1H), 7.45–7.50 (overlap, 2H), 7.56–7.62 (overlap, major and minor rotamer, 1H), 7.79 (br dd, *J* = 7.2, 7.2 Hz, 0.59H, major rotamer), 7.81 (br dd, *J* = 7.2, 7.2 Hz, 0.41H, minor rotamer), 8.08 (d, *J* = 7.2 Hz, 1.18H, major rotamer), 8.12 (d, *J* = 7.2 Hz, 0.82H, minor rotamer), 8.40–8.52 (overlap, 2H); ^{13}C NMR (100 MHz, $CDCl_3$, assumed rotamer pairs in parentheses) δ (3.7, 3.8), 21.9, (33.9, 38.4), (48.0, 51.4), (72.9, 73.0), (80.9, 81.9), (115.06, 115.10), (119.19, 119.21), (123.0, 123.1), (125.79, 125.81), 127.9, (128.21, 128.23, 2C), (128.4, 128.6, 2C), (128.88, 128.90, 2C), (130.2, 130.3, 2C), (133.19, 133.21), (134.48, 134.51), (135.4, 136.2), (139.9, 140.0), 144.2, (146.7, 146.8), 146.9, (157.0, 157.4), (164.8, 164.9); HRMS (ESI) *m*/*z* 510.1581 ([M + H]$^+$, 100%), calcd for $C_{28}H_{23}N_5O_3S$ 510.1600.

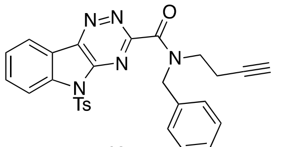

13g

***N*-Benzyl-*N*-(but-3-yn-1-yl)-5-[(4-methylbenzene)sulfonyl]-5*H*-[1,2,4]triazino[5,6-*b*]indole-3-carboxamide (13g).** Prepared according to general procedure C, beginning with **8a** (80 mg, 0.2 mmol, 1.0 equiv) and benzyl(but-3-yn-1-yl)amine [5] (48 mg, 0.3 mmol, 1.5 equiv). Purification by flash chromatography (DCM/EtOAc, 20:1, R_f 0.38) gave **13g** (96 mg, 0.19 mmol, 93% yield) as a brown-orange oil: IR (neat) 2947, 2833, 1655, 1450, 1373, 1191, 1179, 1036, 667 cm^{-1}; ^{1}H NMR (400 MHz, $CDCl_3$) δ 1.97 (t, *J* = 2.7 Hz, 0.48H, minor rotamer), 2.07 (t, *J* = 2.7 Hz, 0.52H, major rotamer), 2.33 (s, 3H), 2.51 (td, *J* = 3.2, 2.7 Hz, 0.96H, minor rotamer), 2.64 (td, *J* = 3.2, 2.7 Hz, 1.04H, major rotamer), 3.41 (t, *J* = 3.2 Hz, 0.96H, minor rotamer), 3.70 (t, *J* = 3.2 Hz, 1.04H, major rotamer), 4.61 (s, 1.04H, major rotamer), 4.97 (s, 0.96H, minor rotamer), 7.21–7.30 (overlap, 2H), 7.34 (m, 2H), 7.38–7.48 (overlap, 3H), 7.55 (br dd, *J* = 8.2, 7.8 Hz, 0.52H, major rotamer), 7.57 (br dd, *J* = 8.2, 7.8 Hz, 0.48H, minor rotamer), 7.78 (ddd, *J* = 8.2, 7.8, 1.2 Hz, 0.48H, minor rotamer), 7.81 (ddd, *J* = 8.2, 7.8, 1.8 Hz, 0.52H, major rotamer), 8.06 (d, *J* = 8.4 Hz, 1.04H, major rotamer), 8.10 (d, *J* = 8.4 Hz, 0.96H, minor rotamer), 8.41 (br d, *J* = 8.2 Hz, 0.52H, major rotamer), 8.44 (br d, *J* = 8.2 Hz, 0.48H, minor rotamer), 8.46 (d, *J* = 8.2 Hz, 0.48H, minor rotamer), 8.50 (d, *J* = 8.2 Hz, 0.52H, major rotamer); ^{13}C NMR (75 MHz, $CDCl_3$, assumed rotamer pairs in parenthesis) δ (17.4, 18.8), 21.8, (44.2, 46.9), (48.6, 53.5), (70.2, 71.2), (80.6, 81.6), (115.0, 115.1), 119.1, (122.96, 123.01), 125.8, 127.9, (128.18, 128.20, 2C), (128.26, 128.30, 2C), 129.0 (2C), 130.3 (2C), (133.17, 133.23), (134.45, 134.47), (135.7, 136.4), (139.82, 139.94), (144.12, 144.22), 146.6, (146.85, 146.92), (157.27, 157.41), (165.4, 165.7); HRMS (ESI) *m*/*z* 532.1429 ([M + Na]$^+$, 100%), calcd for $C_{28}H_{23}N_5O_3SNa$ 532.1419.

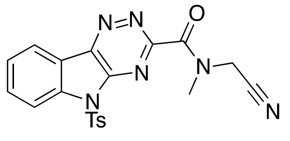

13h

***N*-(Cyanomethyl)-*N*-methyl-5-[(4-methylbenzene)sulfonyl]-5*H*-[1,2,4]triazino[5,6-*b*]indole-3-carboxamide (13h).** Prepared according to general procedure C, starting with **8a** (80 mg, 0.20 mmol, 1.0 equiv) and methylaminoacetonitrile hydrochloride (25.8 mg, 0.24 mmol, 1.2 equiv). Purification by flash chromatography (DCM/EtOAc, 10:1, R_f = 0.41) gave **13h (**80 mg, 0.19 mmol, 95% yield) as a brown-orange oil: IR (neat) 2924, 2251, 1666, 1374, 1193, 1179, 737, 667 cm^{-1}; ^1H NMR (300 MHz, $CDCl_3$) δ 2.34 (s, 2.25H, minor rotamer), 2.35 (s, 2.25, major rotamer), 3.23 (s, 2.25H, major rotamer), 3.37 (s, 0.75H, minor rotamer), 4.58 (s, 0.5H, minor rotamer), 4.65 (s, 1.5H, major rotamer), 7.29 (d, *J* = 8.4 Hz, 2H), 7.575 (dd, *J* = 7.8, 7.5 Hz, 0.25H, minor rotamer), 7.583 (dd, *J* = 7.8, 7.5 Hz, 0.75H, major rotamer), 7.82 (dd, *J* = 8.1, 7.8 Hz, 1H), 8.09 (d, *J* = 8.4 Hz, 1.5H, major rotamer), 8.11 (d, *J* = 8.4 Hz, 0.5H, minor rotamer), 8.41 (br d, *J* = 7.5 Hz, 0.25H, minor rotamer), 8.44 (br d, *J* = 7.5 Hz, 0.75H, major rotamer), 8.48 (d, *J* = 8.1 Hz, 1H); ^{13}C NMR (75 MHz, $CDCl_3$, assumed rotamer pairs in parentheses) δ 21.9, (34.4, 35.6), (37.1, 40.0), 114.7, 115.1, 118.8, 123.1, 126.0, 128.3 (2C), 130.4 (2C), 133.6, 134.2, 140.1, 144.5, 146.6, 147.1, (155.2, 155.6), (164.0, 164.9); HRMS (ESI) *m*/*z* 443.0902 ([M + Na]$^+$, 100%), calcd for $C_{20}H_{16}N_6O_3SNa$ 443.0902.

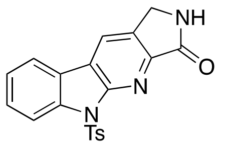

14b

8-[(4-Methylbenzene)sulfonyl]-8,10,13-triazatetracyclo[7.7.0.0^{2,7}.0^{11,15}] hexadeca-1(16),2,4,6,9,11(15)-hexaen-12-one (14b). Prepared according to general procedure D, starting with **13b** (30 mg, 0.074 mmol), in DMF (0.1 M), at 160 °C for 30 min. Purification by flash chromatography (DCM/MeOH, 10:1, R_f = 0.34) gave **14b** (23 mg, 0.061 mmol, 83% yield) as a light-yellow solid: m.p. 304 °C; IR (neat) 3206, 2926, 2857, 1681, 1376, 1175, 1091 cm^{-1}; ^{1}H NMR (400 MHz, DMSO-d_6) δ 2.28 (s, 3H), 4.49 (s, 2H), 7.34 (d, J = 8.4 Hz, 2H), 7.52 (dd, J = 7.6, 7.2 Hz, 1H), 7.70 (dd, J = 8.2 Hz, 7.6 Hz, 1H), 8.00 (d, J = 8.4 Hz, 2H), 8.30 (d, J = 7.2 Hz, 1H), 8.39 (d, J = 8.2 Hz, 1H), 8.78 (s, 1H), 9.10 (br s, NH); ^{13}C NMR (100 MHz, DMSO) δ 21.1, 42.5, 114.5, 120.2, 122.0, 122.2, 124.4, 125.2, 127.2 (2C), 129.5, 130.0 (2C), 134.2, 134.6, 137.9, 145.8, 148.0, 150.9, 167.5; HRMS (ESI) m/z 378.0920 ([M + H]$^+$, 100%), calcd for $C_{20}H_{15}N_3O_3S$ 378.0912.

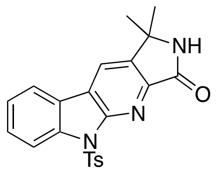

14c

14,14-Dimethyl-8-[(4-methylbenzene)sulfonyl]-8,10,13-triazatetracyclo-[7.7.0.0^{2,7}.0^{11,15}]hexadeca-1(16),2(7),3,5,9,11(15)-hexaen-12-one (14c). Prepared according to general procedure D, starting with **14c** (30 mg, 0.069 mmol). Purification by flash chromatography (100% EtOAc, R_f = 0.26) gave **14c (**25 mg, 0.069 mmol, 91% yield) as an off-white paste: IR (neat) 3218, 3068, 2977, 1704, 1375, 1190, 1177, 910, 738 cm^{-1}; ^{1}H NMR (400 MHz, $CDCl_3$) δ 1.63 (s, 6H), 2.31 (s, 3H), 7.23 (d, J = 8.2 Hz, 2H), 7.41 (dd, J = 8.4, 7.6 Hz, 1H), 7.45 (br s, N*H*), 7.62 (dd, J = 8.0, 8.0 Hz, 1H), 7.99 (br d, J = 7.6 Hz, 1H), 8.21 (s, 1H), 8.23 (d, J = 8.2 Hz, 2H), 8.52 (d, J = 8.4 Hz, 1H); ^{13}C NMR (100 MHz, $CDCl_3$) δ 21.8, 28.2 (2C), 57.1, 115.3, 121.20, 121.24, 121.3, 121.9, 124.0, 128.6 (2C), 129.67, 129.74 (2C), 135.7, 139.2, 142.7, 145.4, 146.2, 151.7, 167.2; HRMS (ESI) m/z 428.1035 ([M + Na]$^+$, 100%), calcd for $C_{22}H_{19}N_3O_3SNa$ 428.1045.

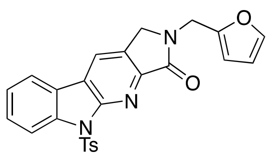

14d

13-(Furan-2-ylmethyl)-8-[(4-methylbenzene)sulfonyl]-8,10,13-triazatetracyclo[7.7.0.0^{2,7}.0^{11,15}]hexadeca-1(16),2(7),3,5,9,11(15)-hexaen-12-one (14d). Prepared according to general procedure D, starting with **13d** (30 mg, 0.062 mmol). Purification by flash chromatography (DCM/EtOAc, 10:1, R_f = 0.38) gave **14d** (27 mg, 0.060 mmol, 98% yield) as a white solid: mp 251–252 °C; IR (neat) 2939, 2833, 1687, 1374, 1174, 1033, 751, 540 cm^{-1}; ^{1}H NMR (400 MHz, $CDCl_3$) δ 2.30 (s, 3H), 4.40 (s, 2H), 4.85 (s, 2H), 6.33 (br s, 1H), 6.36 (d, J = 3.2 Hz, 1H), 7.22 (d, J = 8.4 Hz, 2H), 7.33 (overlap dd, J = 8.0, 7.6 Hz, 1H), 7.34 (overlap br s, 1H) 7.52 (dd, J = 7.9, 7.6 Hz, 1H), 7.87 (d, J = 7.9 Hz, 1H), 8.13 (s, 1H), 8.21 (d, J = 8.4 Hz, 2H), 8.44 (d, J = 8.0 Hz, 1H); ^{13}C NMR (125 MHz, $CDCl_3$) δ 21.8, 39.7, 47.4, 105.1, 109.1, 110.7, 115.1, 120.6, 121.2, 121.7, 122.8, 123.9, 128.7 (2C), 129.4, 129.7 (2C), 130.9, 135.6, 139.0, 142.9, 145.4, 147.6, 150.1, 151.4, 165.8; HRMS (ESI) m/z 480.0988 ([M + Na]$^+$, 100%), calcd for $C_{25}H_{19}N_3O_4SNa$ 480.0994.

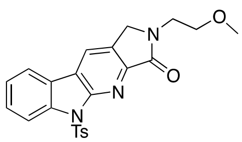

14e

13-(2-Methoxyethyl)-8-[(4-methylbenzene)sulfonyl]-8,10,13-triazatetracyclo[7.7.0.0^{2,7}.0^{11,15}]hexadeca-1(16),2(7),3,5,9,11(15)-hexaen-12-one (14e). Prepared according to general procedure D, starting with **13e** (30 mg, 0.065 mmol). Purification by flash chromatography (DCM/EtOAc, 1:1, R_f = 0.14) gave **14e** (27 mg, 0.062 mmol, 97% yield) as a white-yellow solid: mp 247–248 °C; IR (neat) 3052, 2922, 1703, 1376, 1192, 1176, 742, 664 cm^{-1}; ^{1}H NMR (400 MHz, $CDCl_3$) δ 2.27 (s, 3H), 3.43 (s, 3H), 3.67 (t, J = 4.7 Hz, 2H), 3.86 (t, J = 4.7 Hz, 2H), 4.57 (s, 2H), 7.19 (d, J = 8.4 Hz, 2H), 7.29 (dd, J = 8.2, 8.0 Hz, 1H), 7.48 (dd, J = 8.0, 7.8 Hz, 1H), 7.81 (d, J = 7.8 Hz, 1H), 8.09 (s, 1H), 8.17 (d, J = 8.4 Hz, 2H), 8.37 (d, J = 8.2 Hz, 1H); ^{13}C NMR (75 MHz, $CDCl_3$) δ 21.7, 43.1, 49.2, 58.9, 71.5, 114.9, 120.4, 121.2, 121.7, 122.7, 123.8, 128.5 (2C), 129.2, 129.7 (2C),

131.4, 135.6, 138.7, 145.4, 147.6, 151.2, 166.2; HRMS (ESI) *m*/*z* 458.1158 ($[M + Na]^+$, 100%), calcd for $C_{23}H_{21}N_3O_4SNa$ 458.1150.

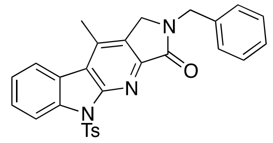

14f

13-Benzyl-16-methyl-8-[(4-methylbenzene)sulfonyl]-8,10,13-triaza-tetracyclo[7.7.0.$0^{2,7}$.$0^{11,15}$]hexadeca-1(16),2(7),3,5,9,11(15)-hexaen-12-one (14f). Prepared according to general procedure D, starting with **13f** (35 mg, 0.069 mmol). Purification by flash chromatography (hexane/EtOAc, 3:1, R_f = 0.19) gave **14f** (31 mg, 0.064 mmol, 93% yield) as a white solid: Decomposition at 238 °C; IR (neat) 2952, 2854, 1707, 1596, 1177, 1090 cm^{-1}; ^1H NMR (400 MHz, $CDCl_3$) δ 2.30 (s, 3H), 2.64 (s, 3H), 4.23 (s, 2H), 4.85 (s, 2H), 7.20–7.40 (overlap, 9H), 7.55 (br dd, *J* = 7.6, 7.6 Hz, 1H), 7.97 (br d, *J* = 7.2 Hz, 1H), 8.21 (d, *J* = 6.4 Hz, 2H), 8.54 (d, *J* = 7.6 Hz, 1H); ^{13}C NMR (100 MHz, $CDCl_3$) δ 16.3, 21.8, 46.4, 47.3, 115.0, 119.1, 122.7, 123.2, 123.9, 128.0, 128.6 (2C), 128.7, 128.8 (2C), 129.0 (2C), 129.6 (2C), 130.9, 135.6, 136.9, 137.2, 138.8, 145.3, 147.1, 151.7, 166.5; HRMS (ESI) *m*/*z* 482.1532 ($[M + H]^+$, 100%), calcd for $C_{28}H_{23}N_3O_3S$ 482.1538.

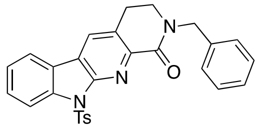

14g

2-Benzyl-10-[(4-methylbenzene)sulfonyl]-1*H*,2*H*,3*H*,4*H*,10*H*-indole[2,3-*b*]1,7-naphthyridin-1-one (14g). Prepared according to general procedure D, starting with **13g** (30 mg, 0.058 mmol, 1.0 equiv), at 160 °C. Purification by flash chromatography (100% DCM, R_f = 0.20) gave **14g** (27 mg, 0.056 mmol, 95% yield) as a brown solid: mp 232–233 °C; IR (neat) 2922, 2852, 1659, 1374, 1176, 669, 580 cm^{-1}; ^1H NMR (300 MHz, $CDCl_3$) δ 2.22 (s, 3H), 2.94 (t, *J* = 6.5 Hz, 2H), 3.43 (t, *J* = 6.5 Hz, 2H), 4.77 (s, 2H), 7.15–7.28 (m, 8H), 7.48 (ddd, *J* = 8.4, 8.4, 1.5 Hz, 1H), 7.78 (d, *J* = 7.5 Hz, 1H), 7.86 (s, 1H), 8.30 (d, *J* = 8.1 Hz, 2H), 8.40 (d, *J* = 8.4 Hz, 1H); ^{13}C NMR (125 MHz, $CDCl_3$) δ 21.8, 27.8, 45.0, 50.7, 115.1, 120.5, 121.4, 121.8, 123.7, 127.1,

127.7, 128.4 (2C), 128.8 (2C), 129.2 (2C), 129.5, 129.6 (2C), 129.8, 135.7, 137.3, 139.2, 143.5, 145.3, 150.0, 163.0; HRMS (ESI) *m/z* 504.1376 ($[M + Na]^+$, 100%), calcd for $C_{28}H_{23}N_3O_3SNa$ 504.1358.

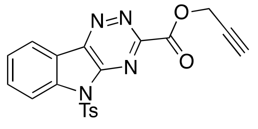

17a

Prop-2-yn-1-yl 5-[(4-methylbenzene)sulfonyl]-5*H*-[1,2,4]triazino-[5,6-*b*]indole-3-carboxylate (17a). Prepared according to general procedure E, starting with **8a** (40 mg, 1.0 equiv, 0.094 mmol), Otera's catalyst (11 mg, 0.1 equiv, 0.01 mmol) and propargyl alcohol (0.1 mL). Purification by flash chromatography (DCM/EtOAc, 10:1, R_f = 0.80) gave **17a** (34 mg, 0.057 mmol, 84% yield) as an orange oil: IR (neat) 3200, 2924, 1753, 1381, 1179, 1153, 737, 579 cm^{-1}; ^1H NMR (400 MHz, $CDCl_3$) δ 2.37 (s, 3H), 2.62 (t, *J* = 2.4 Hz, 1H), 5.13 (d, *J* = 2.4 Hz, 2H), 7.31 (d, *J* = 8.4 Hz, 2H), 7.60 (dd, *J* = 7.8, 7.6 Hz, 1H), 7.85 (dd, *J* = 8.6, 7.8 Hz, 1H), 8.21 (d, *J* = 8.4 Hz, 2H), 8.51 (d, *J* = 7.6 Hz, 1H), 8.54 (d, *J* = 8.6 Hz, 1H); ^{13}C NMR (100 MHz, $CDCl_3$) δ 21.9, 54.3, 76.2, 77.0, 115.2, 118.8, 123.6, 125.9, 128.9 (2C), 130.2 (2C), 133.9, 134.3, 140.7, 145.2, 146.6, 147.0, 151.9, 161.9; HRMS (ESI) *m/z* 407.0804 ($[M + 1]^+$, 100%), calcd for $C_{20}H_{14}N_4O_4S$ 407.0814.

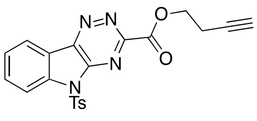

17b

But-3-yn-1-yl 5-[(4-methylbenzene)sulfonyl]-5*H*-[1,2,4]triazino[5,6-*b*]indole-3-carboxylate (17b). Prepared according to general procedure E, starting with **8a** (40 mg, 1.0 equiv, 0.094 mmol), Otera's catalyst (11 mg, 0.1 equiv, 0.01 mmol) and propargyl alcohol (0.1 ml). Purification by flash chromatography (DCM/EtOAc, 20:1, R_f = 0.40) gave **17b** (37 mg, 0.057 mmol, 87% yield) as a yellow-orange oil: IR (neat) 3294, 2961, 1745, 1378, 1178, 1154, 735, 579 cm^{-1}; ^1H NMR (400 MHz, $CDCl_3$) δ 2.08 (t, *J* = 2.8 Hz, 1H), 2.36 (s, 3H), 2.82 (dt, *J* = 6.8, 2.8 Hz, 2H), 4.65 (t, *J* = 6.8 Hz, 2H), 7.30 (d, *J* = 8.6 Hz, 2H), 7.60 (dd, *J* = 8.0, 8.0 Hz, 1H), 7.85 (dd, *J* = 8.0, 8.0 Hz, 1H), 8.21 (d, *J* = 8.6

Hz, 2H), 8.51 (d, J = 8.0 Hz, 1H), 8.54 (d, J = 8.0 Hz, 1H); ^{13}C NMR (100 MHz, $CDCl_3$) δ 19.2 21.9, 64.5, 70.6, 79.7, 115.2, 118.9, 123.5, 125.9, 128.8 (2C), 130.2 (2C), 133.8, 134.4, 140.7, 145.1, 146.7, 147.0, 152.3, 162.4; HRMS (ESI) *m*/*z* 443.0800 ([M + Na]$^+$, 100%), calcd for $C_{21}H_{16}N_4O_4SNa$ 443.0790.

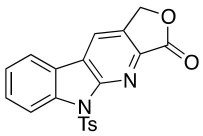

18a

8-[(4-Methylbenzene)sulfonyl]-13-oxa-8,10-diazatetracyclo-[7.7.0.0^{2,7}.0^{11,15}]hexadeca-1(9),2(7),3,5,10,15-hexaen-12-one. Prepared according to general procedure D, starting with **17a** (30 mg, 1.0 equiv, 0.074 mmol), at 160 °C. Purification by flash chromatography (DCM/EtOAc, 5:1, R_f = 0.67, 22 mg) gave **18a** (0.061 mmol, 80% yield) as a yellow oil: IR (neat) 2925, 2360, 2340, 1786, 1379, 1173, 910, 732, 574 cm^{-1}; ^{1}H NMR (400 MHz, $CDCl_3$) δ 2.32 (s, 3H), 5.41 (s, 2H), 7.24 (d, J = 8.4 Hz, 2H), 7.44 (dd, J = 8.0, 7.8 Hz, 1H), 7.67 (dd, J = 8.4, 7.8 Hz, 1H), 8.00 (d, J = 8.4 Hz, 2H), 8.20 (d, J = 8.4 Hz, 2H), 8.28 (s, 1H), 8.54 (d, J = 8.0 Hz, 1H); ^{13}C NMR (100 MHz, $CDCl_3$) δ 21.8, 67.6, 115.4, 121.1, 121.8, 122.0, 122.9, 124.3, 128.7 (2C), 129.7 (2C), 130.7, 135.3, 135.9, 140.0, 140.9, 145.8, 152.5, 168.1; HRMS (ESI) *m*/*z* 401.0584 ([M + Na]$^+$, 100%), calcd for $C_{20}H_{14}N_2O_4SNa$ 401.0572.

References

1. Boger, D. L.; Panek, J. S. Yasuda, M. *Org. Syn.* **1988**, *66*, 142-150.

2. Otera, J.; Dan-oh, N.; Nozaki, H. *J. Org. Chem.* **1991**, *56*, 5307-5311.

3. Luesse, S. B.; Wells, G.; Nayek, A.; Smith, A. E.; Kusche, B. R.; Bergmeier, B. R.; McMills, M. C.; Priestley, N. D.; Wright, D. L. *Bioorg. Med. Chem. Lett.* **2008**, *18*, 3946-3949.

4. Yoshida, S.; Fukui, K.; Kikuchi, S.; Yamada, T. *Chem. Lett.* **2009**, *38*, 786-787.

5. Hess, W.; Burton, J. W. *Chem. Eur. J.* **2010**, *16*, 12303-12306.

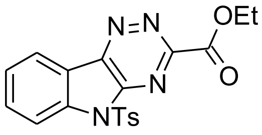
N=N
OEt
O
N
NTs

8a

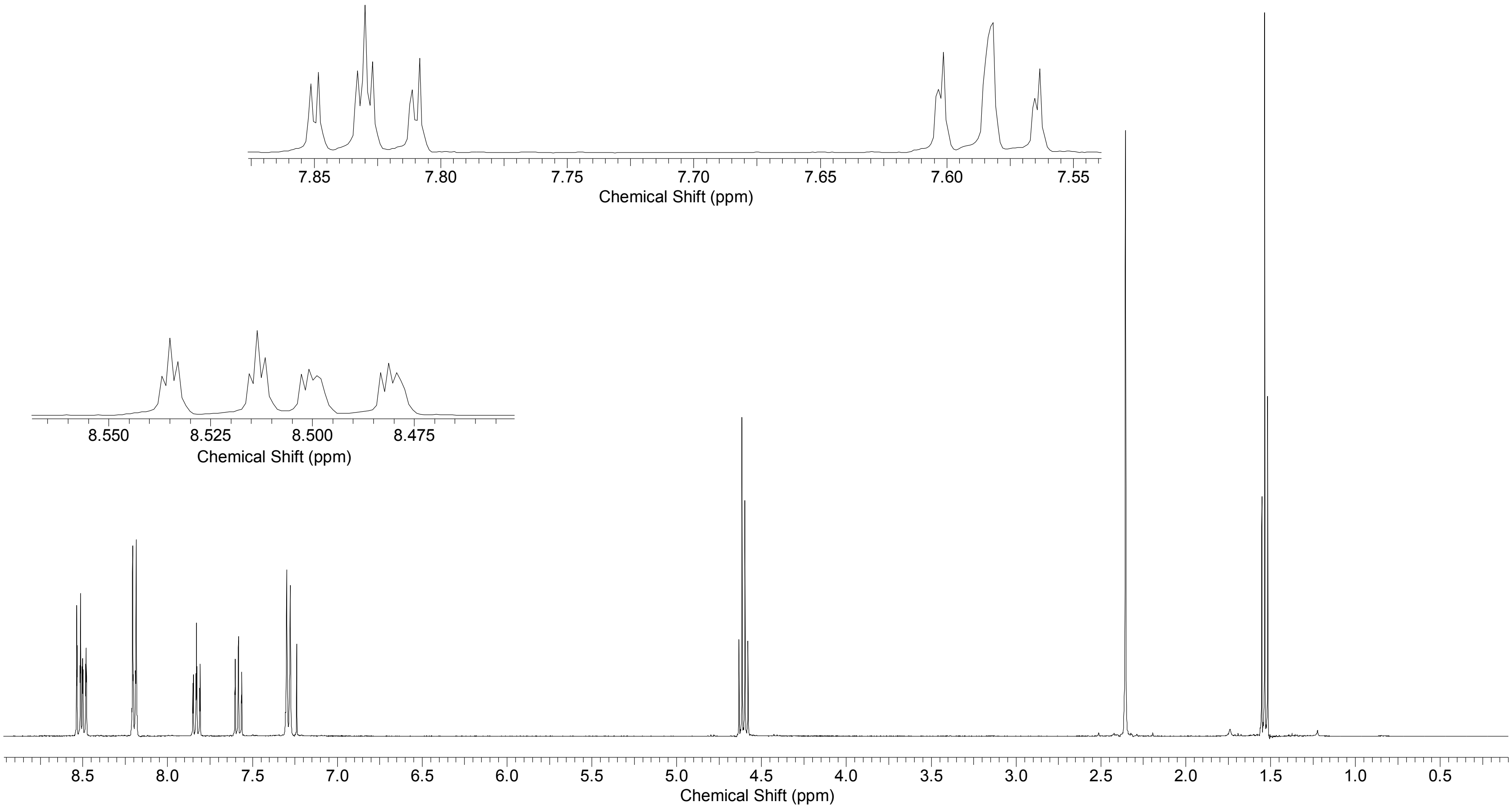
Chemical Shift (ppm)
Chemical Shift (ppm)
Chemical Shift (ppm)

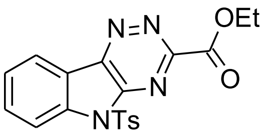

8a

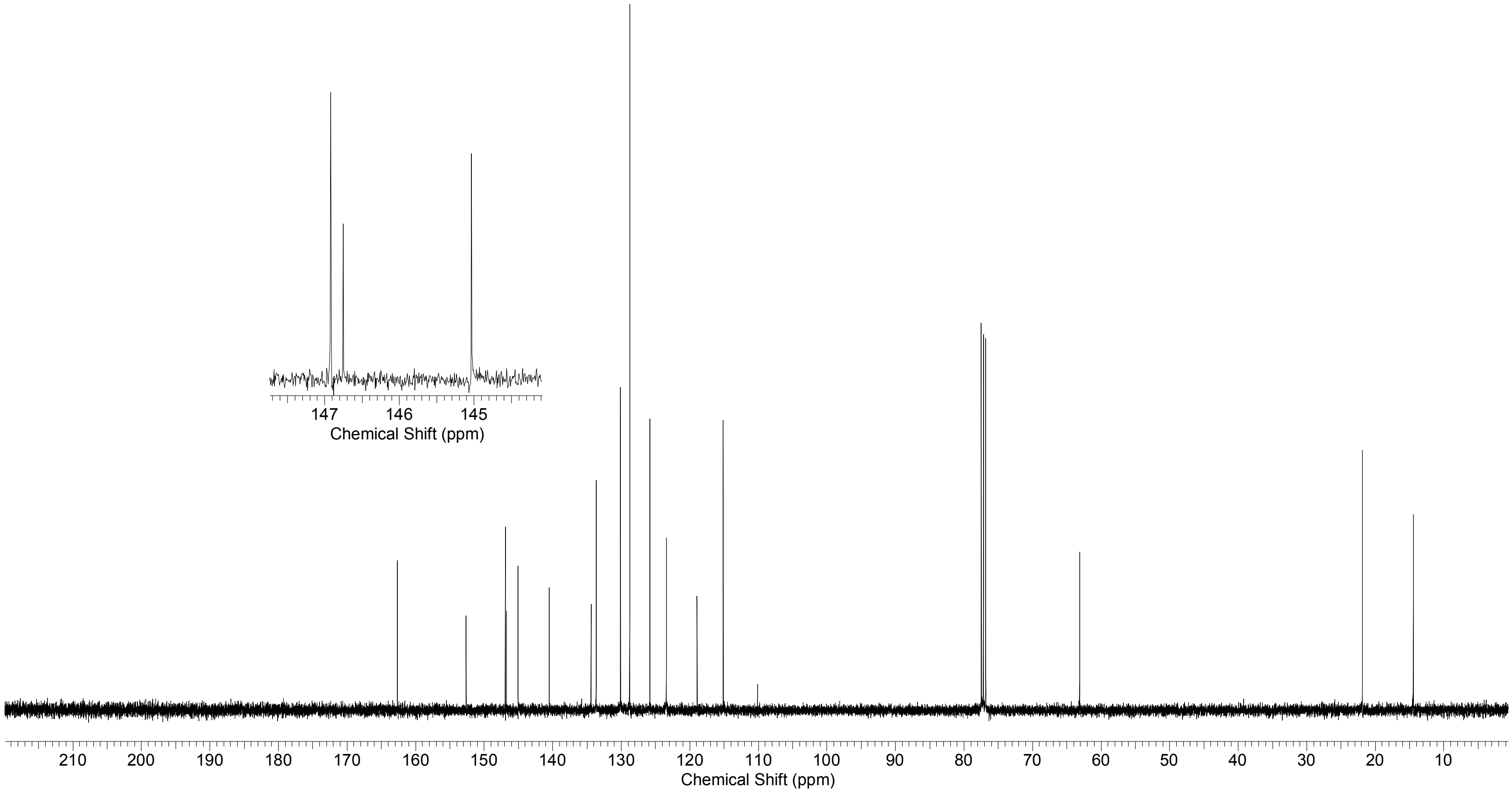
147
146
145
Chemical Shift (ppm)
210
200
190
180
170
160
150
140
130
120
110
100
90
80
70
60
50
40
30
20
10
Chemical Shift (ppm)

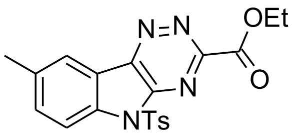

8b

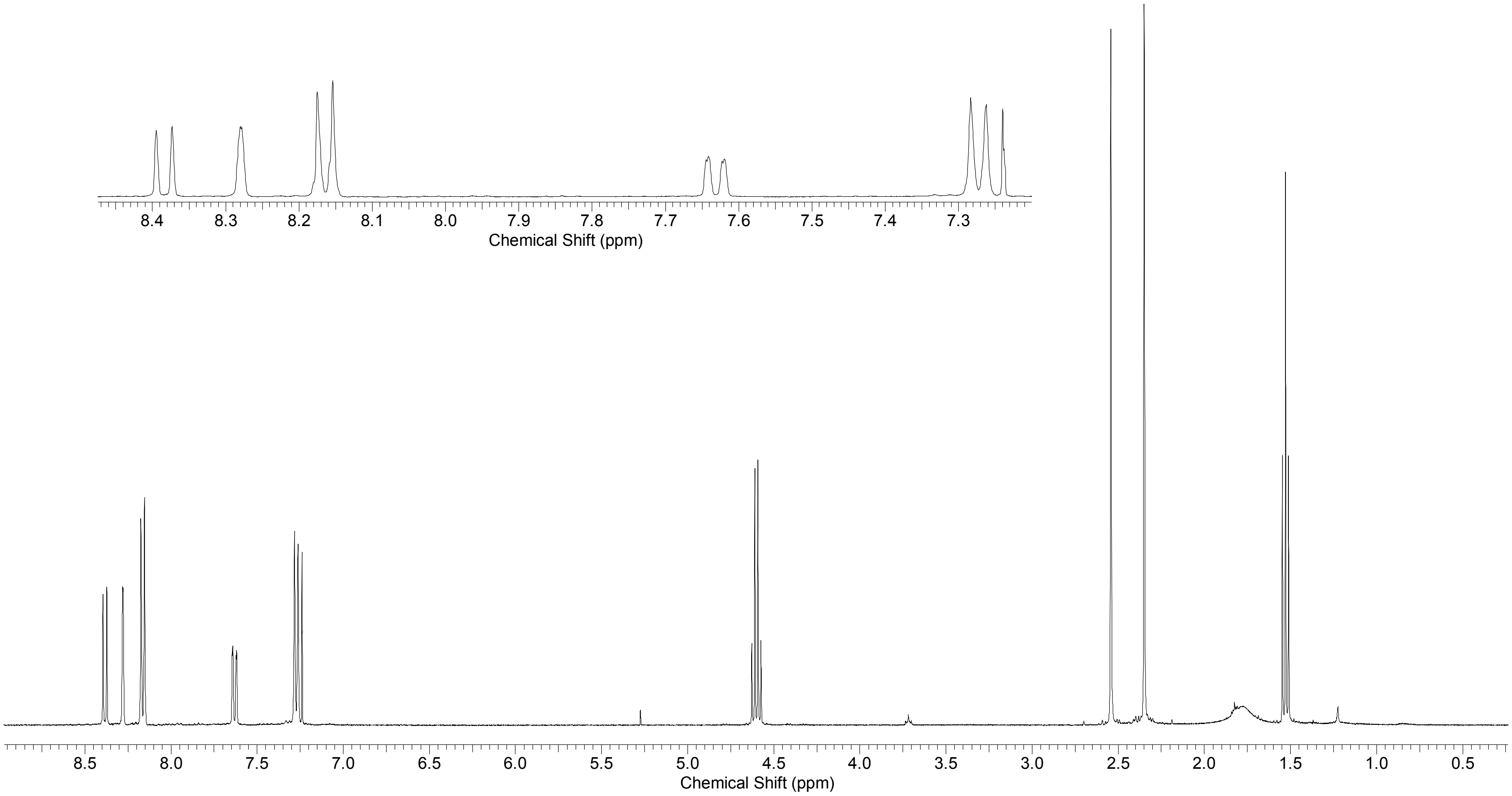
8.4
8.3
8.2
8.1
8.0
7.9
7.8
7.7
7.6
7.5
7.4
7.3
Chemical Shift (ppm)
8.5
8.0
7.5
7.0
6.5
6.0
5.5
5.0
4.5
4.0
3.5
3.0
2.5
2.0
1.5
1.0
0.5
Chemical Shift (ppm)

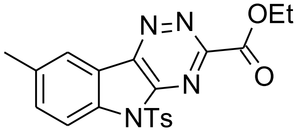
N=N
OEt
O
N
NTs

8b

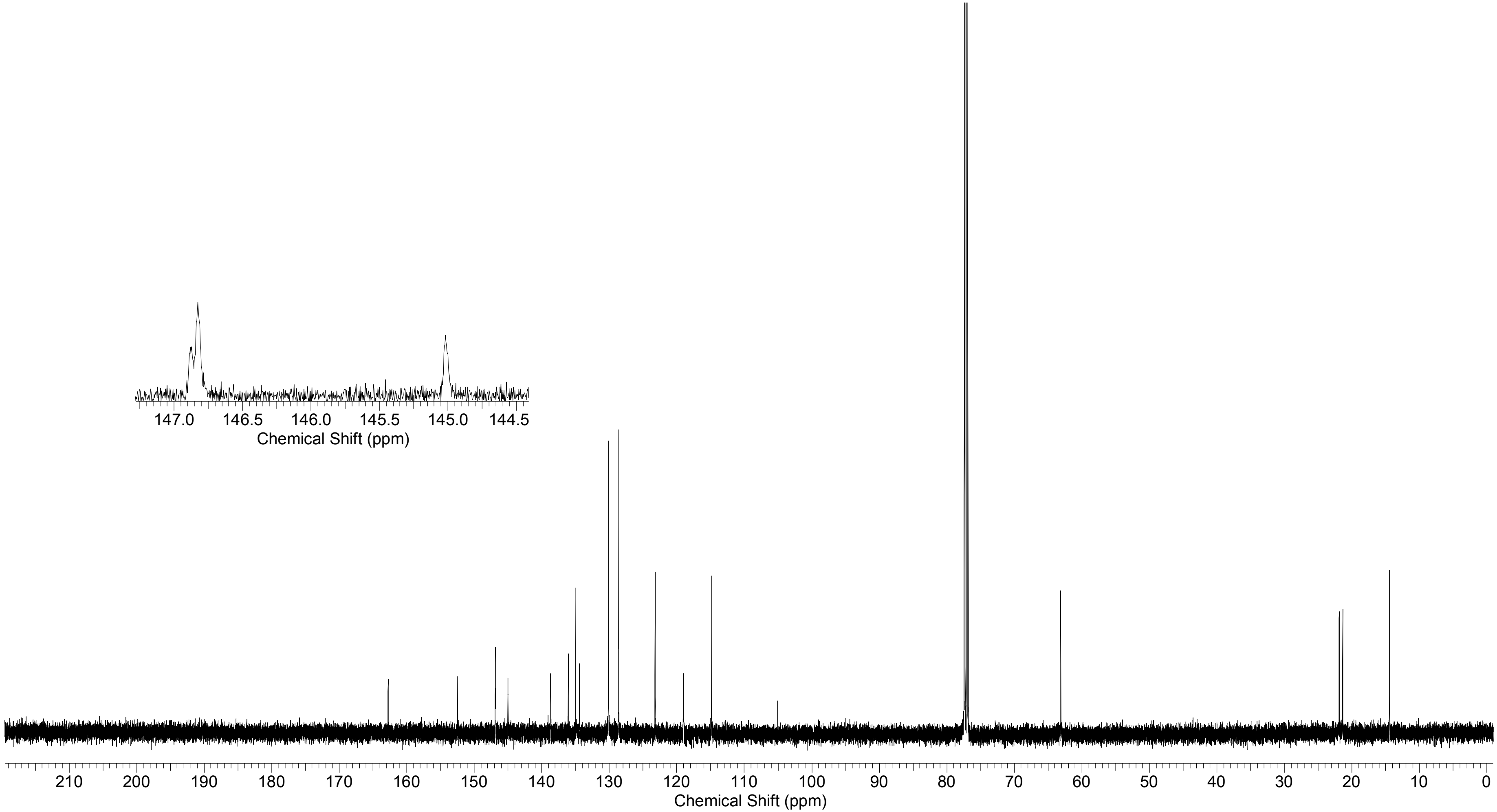
147.0 146.5 146.0 145.5 145.0 144.5
Chemical Shift (ppm)
210 200 190 180 170 160 150 140 130 120 110 100 90 80 70 60 50 40 30 20 10 0
Chemical Shift (ppm)

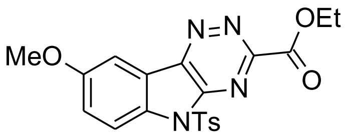
MeO
N=N
OEt
O
N
NTs

8c

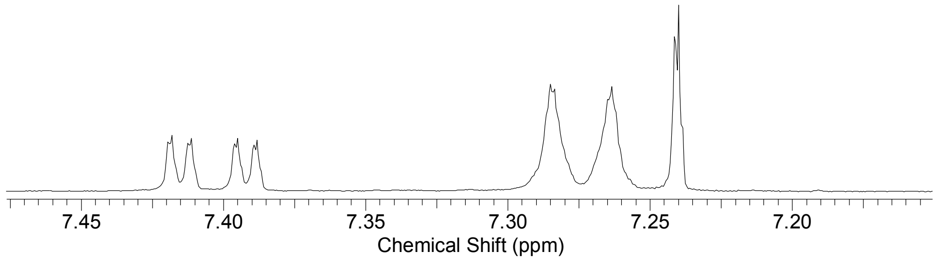
7.45
7.40
7.35
7.30
7.25
7.20
Chemical Shift (ppm)

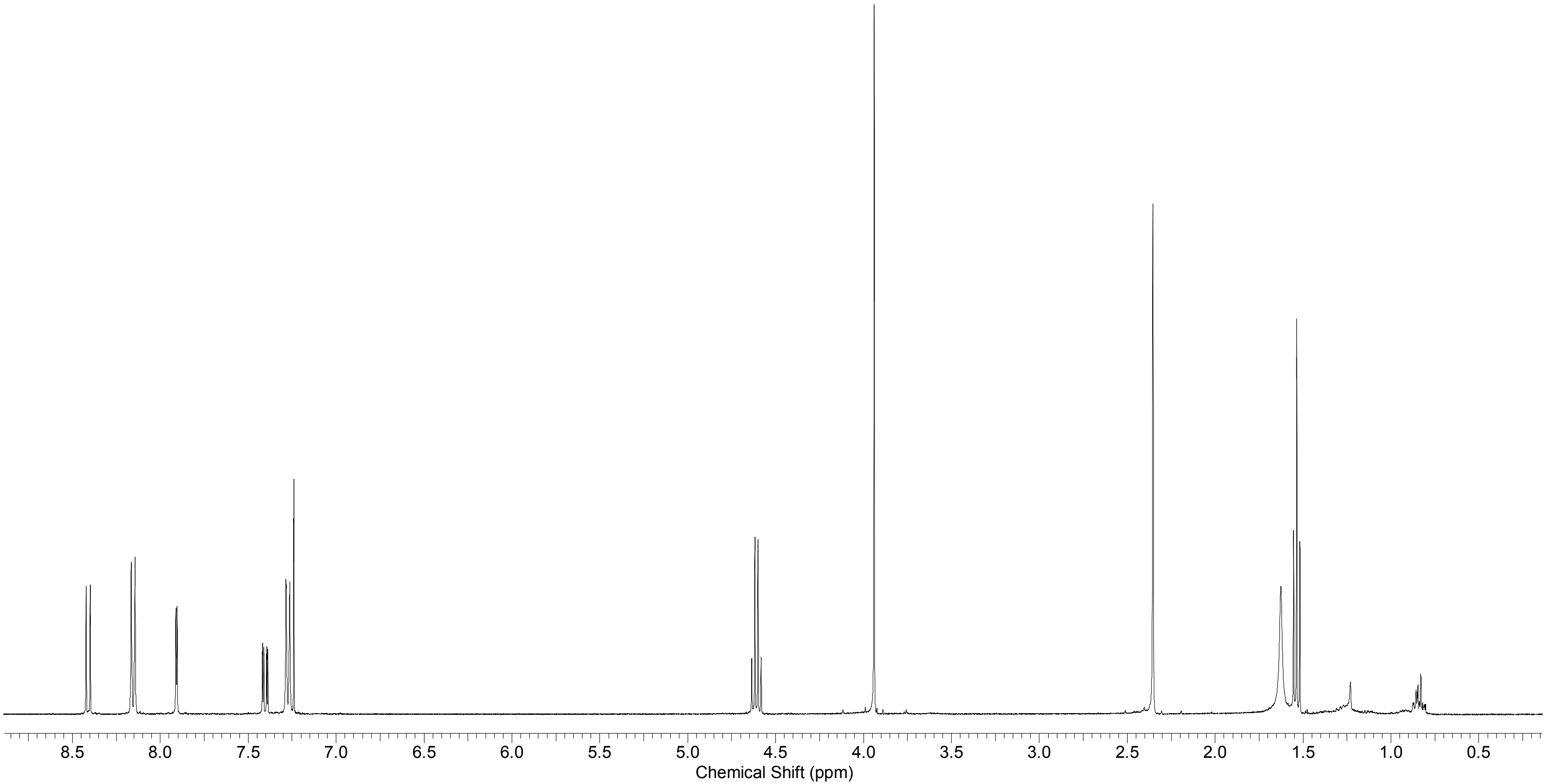
8.5
8.0
7.5
7.0
6.5
6.0
5.5
5.0
4.5
4.0
3.5
3.0
2.5
2.0
1.5
1.0
0.5
Chemical Shift (ppm)

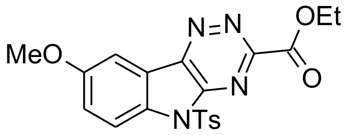
MeO
N=N
OEt
O
N
NTs

8c

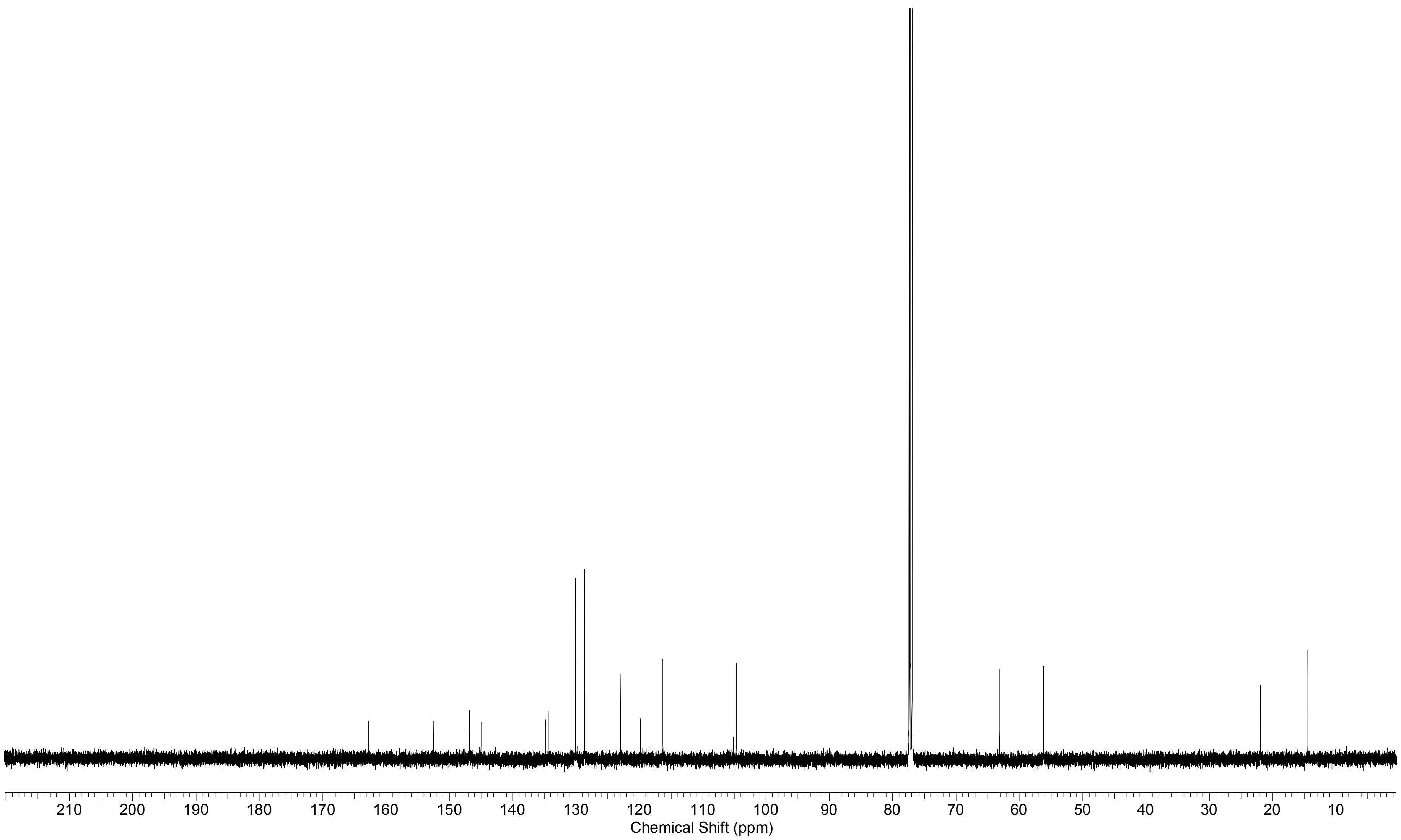
210
200
190
180
170
160
150
140
130
120
110
100
90
80
70
60
50
40
30
20
10
Chemical Shift (ppm)

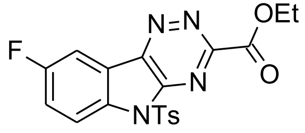
F
N=N
OEt
O
N
NTs

8d

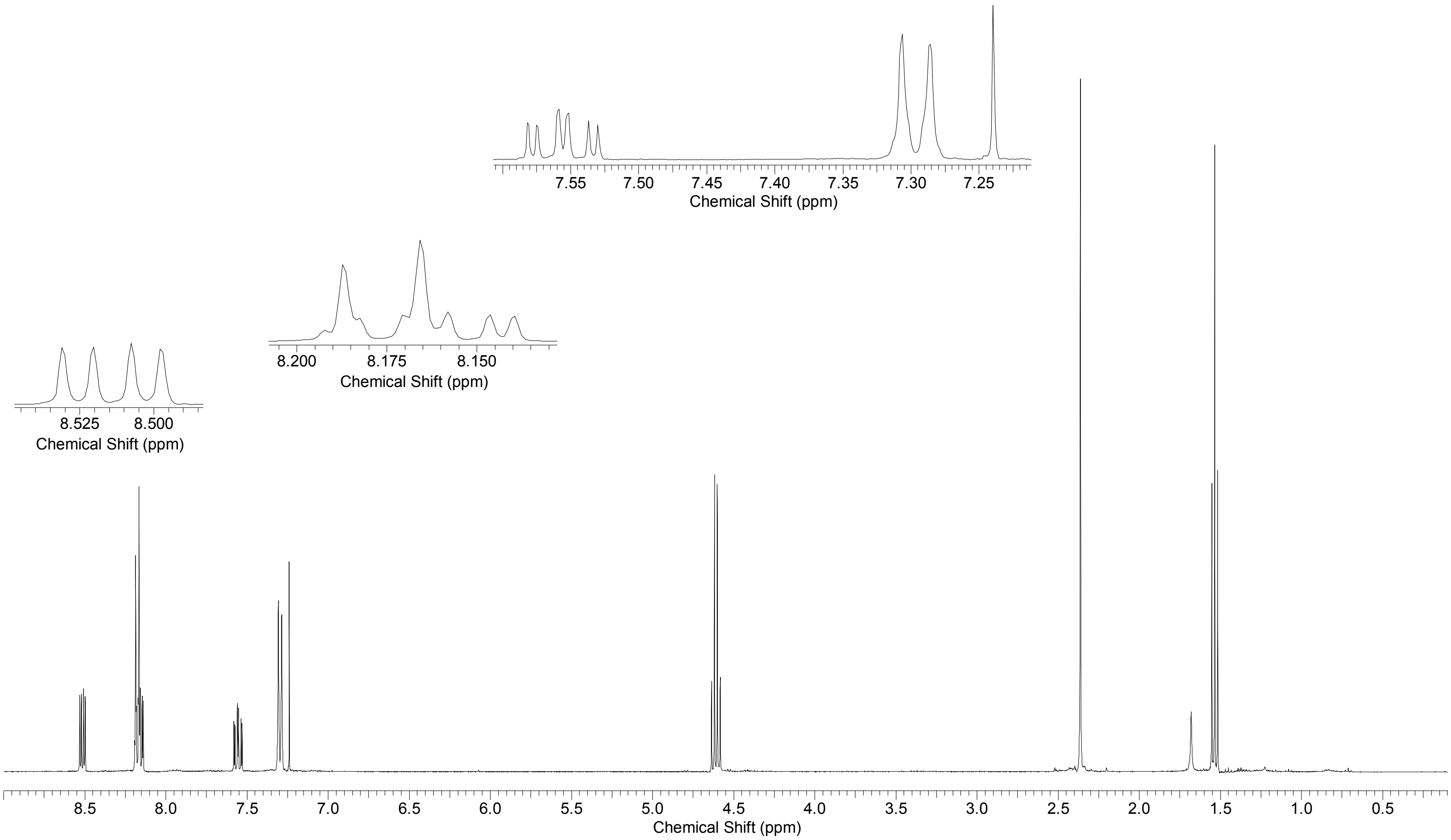
8.525
8.500
Chemical Shift (ppm)
8.200
8.175
8.150
Chemical Shift (ppm)
7.55
7.50
7.45
7.40
7.35
7.30
7.25
Chemical Shift (ppm)
8.5
8.0
7.5
7.0
6.5
6.0
5.5
5.0
4.5
4.0
3.5
3.0
2.5
2.0
1.5
1.0
0.5
Chemical Shift (ppm)

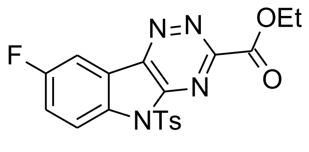
F
N=N
OEt
O
N
NTs

8d

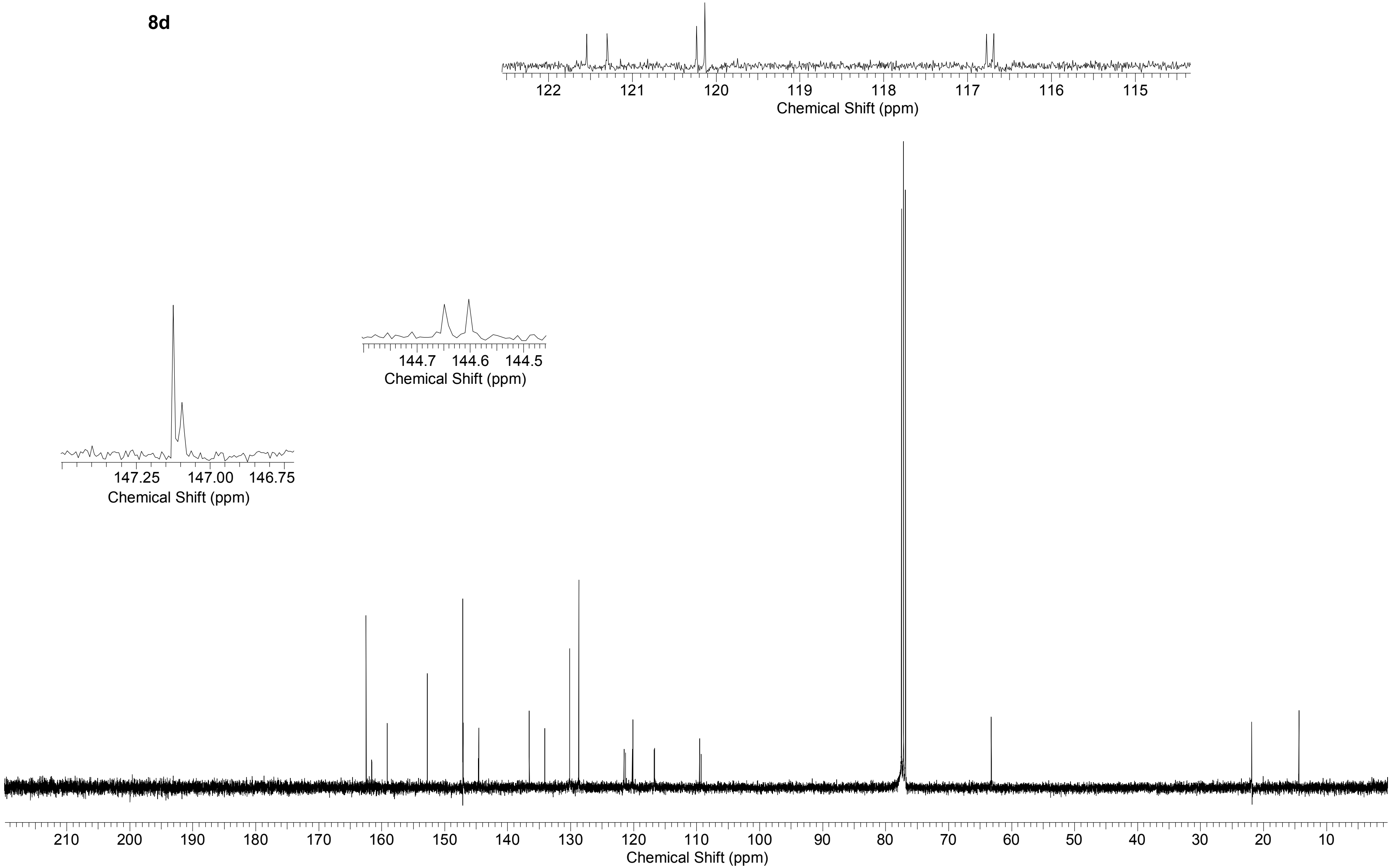
122
121
120
119
118
117
116
115
Chemical Shift (ppm)
144.7
144.6
144.5
Chemical Shift (ppm)
147.25
147.00
146.75
Chemical Shift (ppm)
210
200
190
180
170
160
150
140
130
120
110
100
90
80
70
60
50
40
30
20
10
Chemical Shift (ppm)

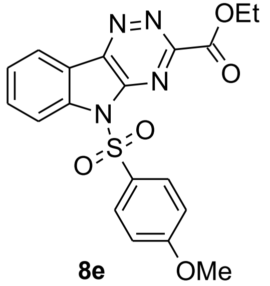

8e

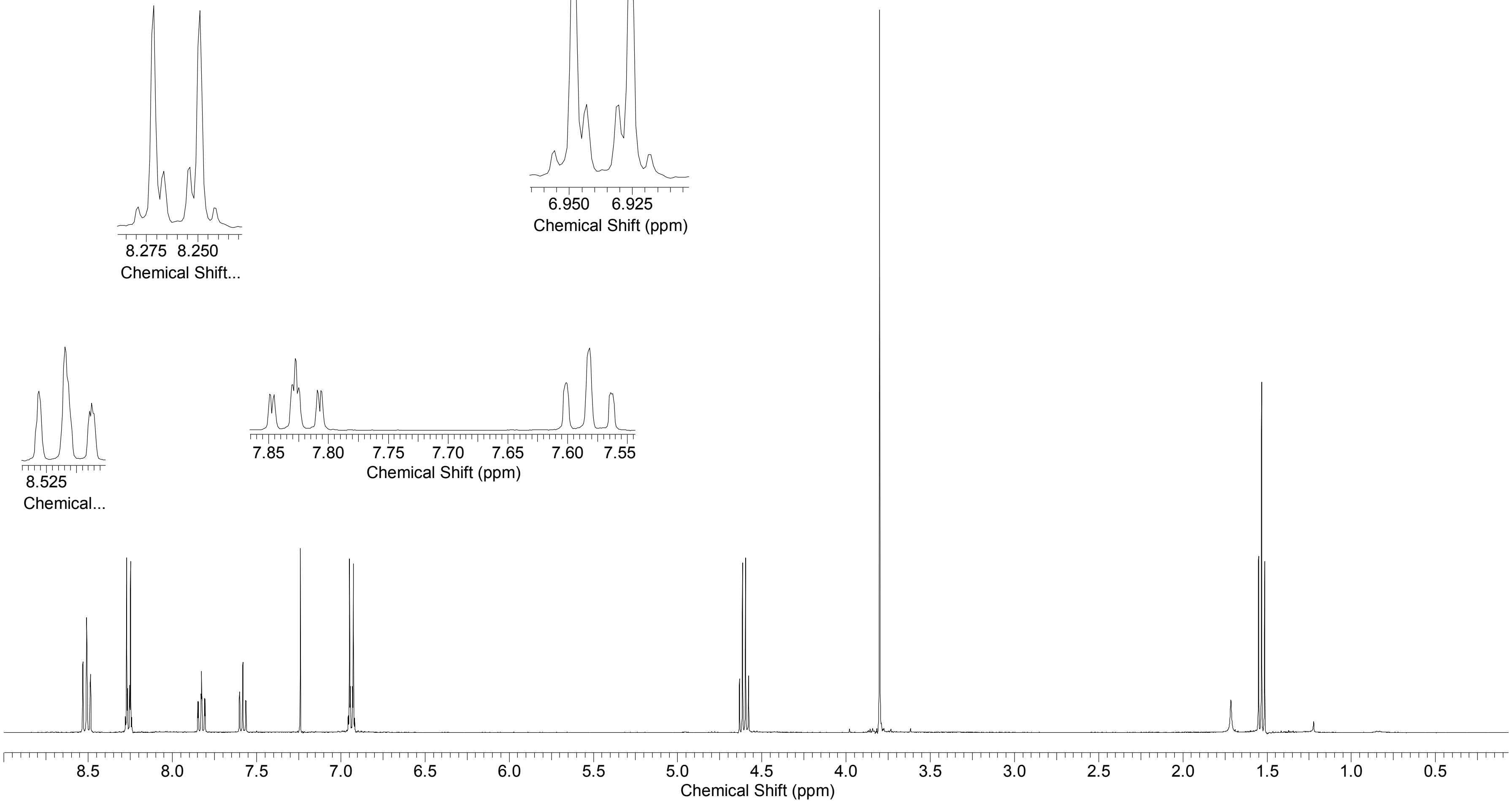

8.525
Chemical...
8.275 8.250
Chemical Shift...
7.85 7.80 7.75 7.70 7.65 7.60 7.55
Chemical Shift (ppm)
6.950 6.925
Chemical Shift (ppm)
8.5 8.0 7.5 7.0 6.5 6.0 5.5 5.0 4.5 4.0 3.5 3.0 2.5 2.0 1.5 1.0 0.5
Chemical Shift (ppm)

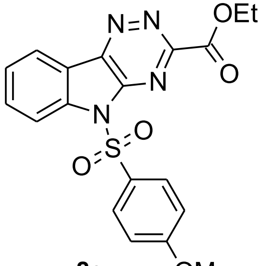
N=N
OEt
O
N
N
S
O
O
OMe

8e

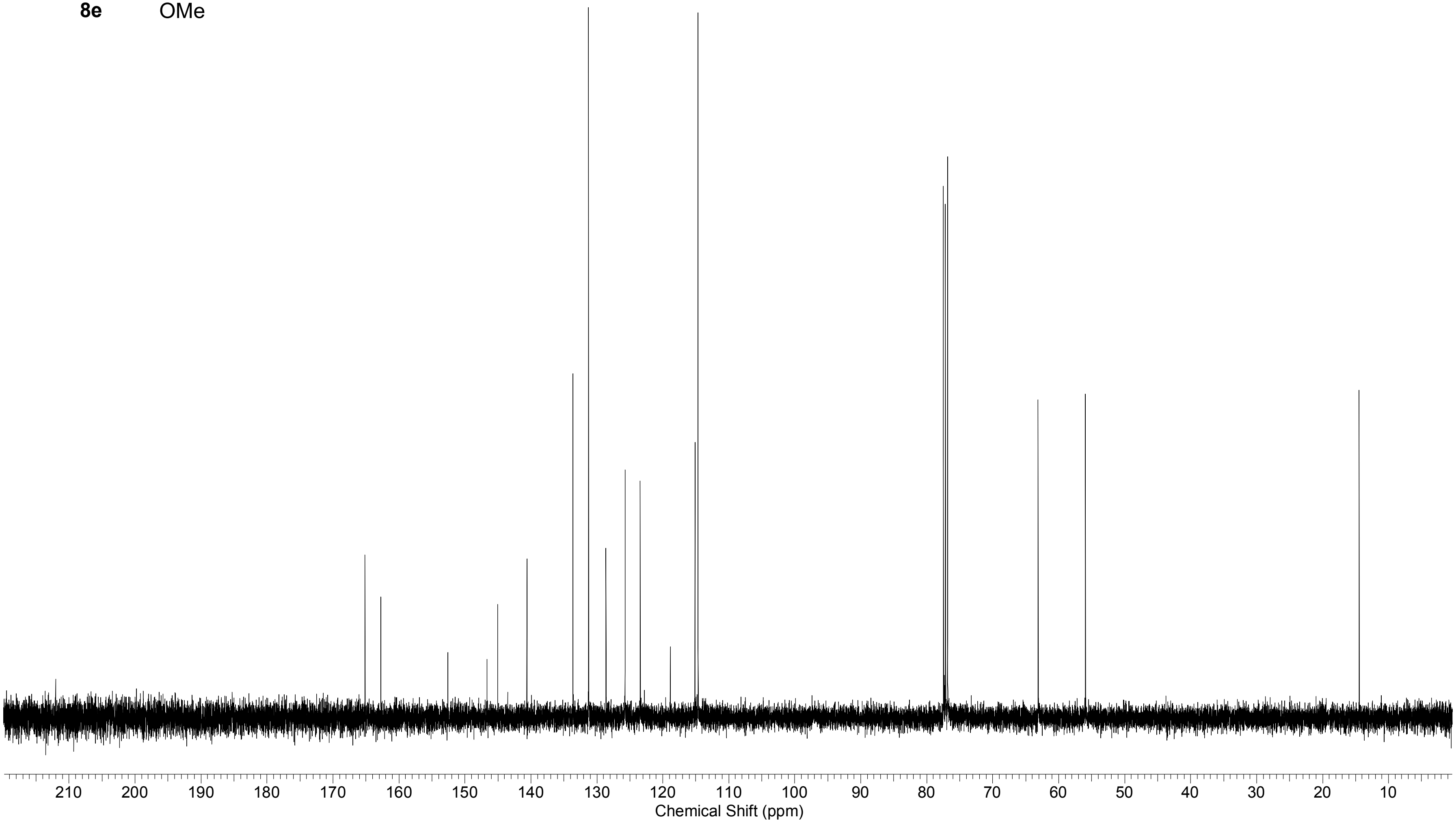
210 200 190 180 170 160 150 140 130 120 110 100 90 80 70 60 50 40 30 20 10
Chemical Shift (ppm)

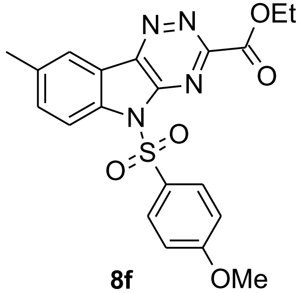
N=N
OEt
O
N
N
O
S
O
OMe

8f

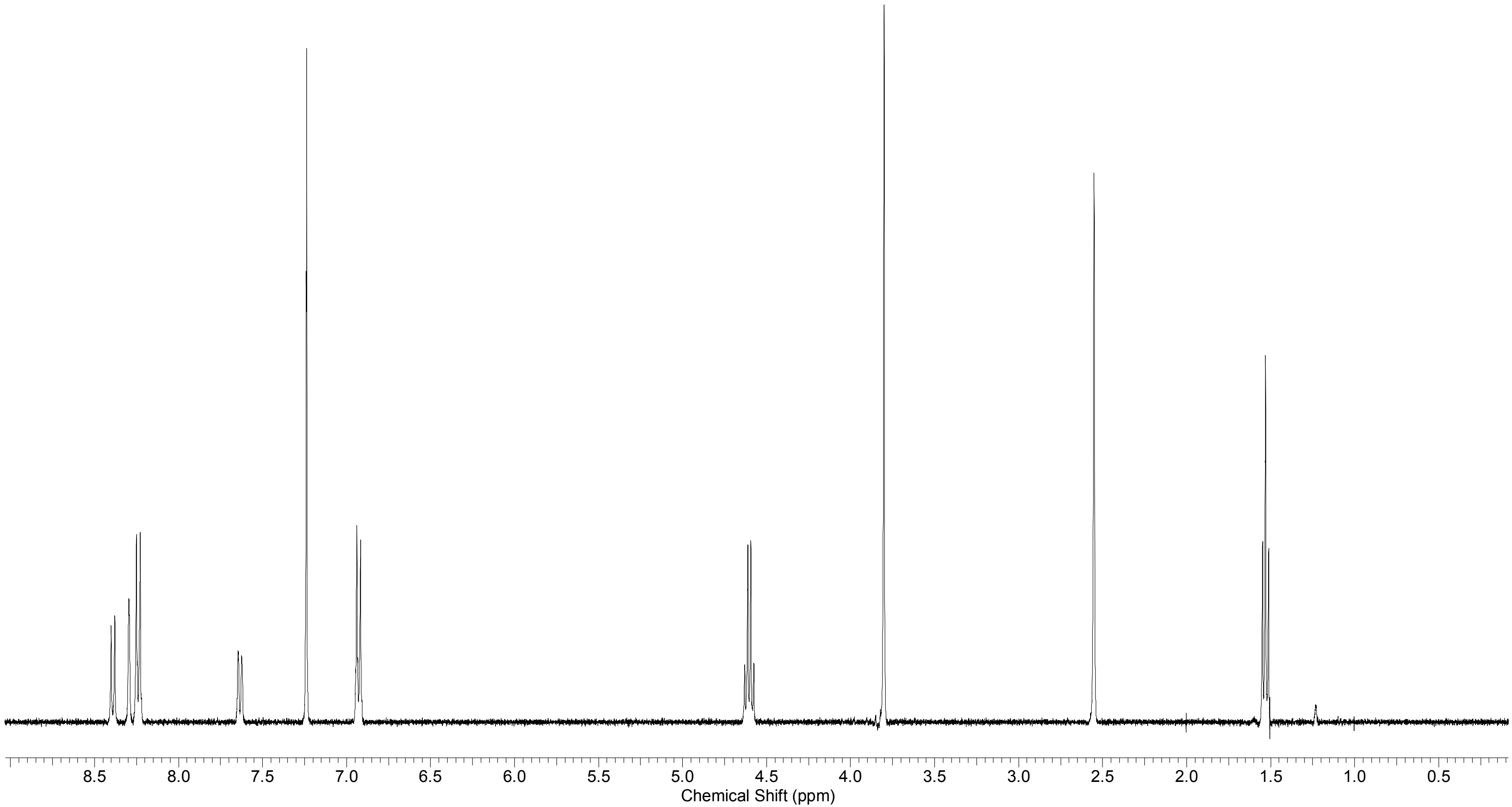
8.5
8.0
7.5
7.0
6.5
6.0
5.5
5.0
4.5
4.0
3.5
3.0
2.5
2.0
1.5
1.0
0.5
Chemical Shift (ppm)

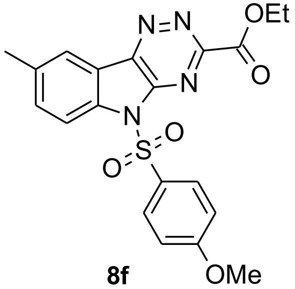
N=N
OEt
O
N
N
O
O=S
OMe

8f

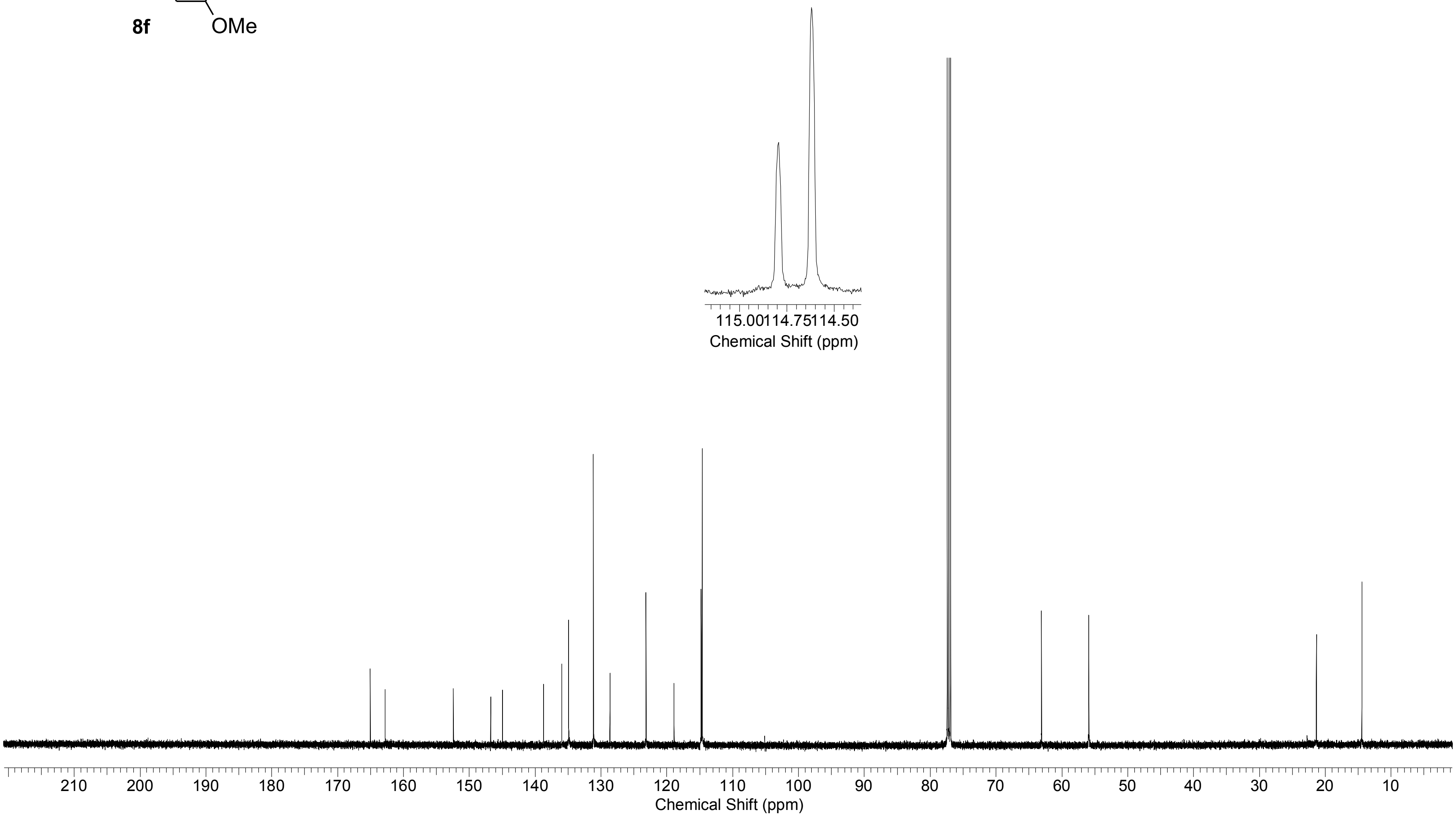
115.00
114.75
114.50
Chemical Shift (ppm)
210 200 190 180 170 160 150 140 130 120 110 100 90 80 70 60 50 40 30 20 10
Chemical Shift (ppm)

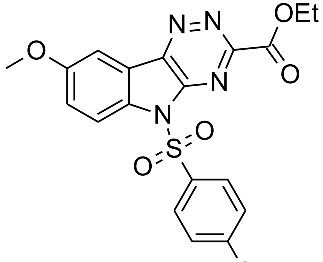
OEt
N=N
O
N
N
S
O
O
OMe

8g

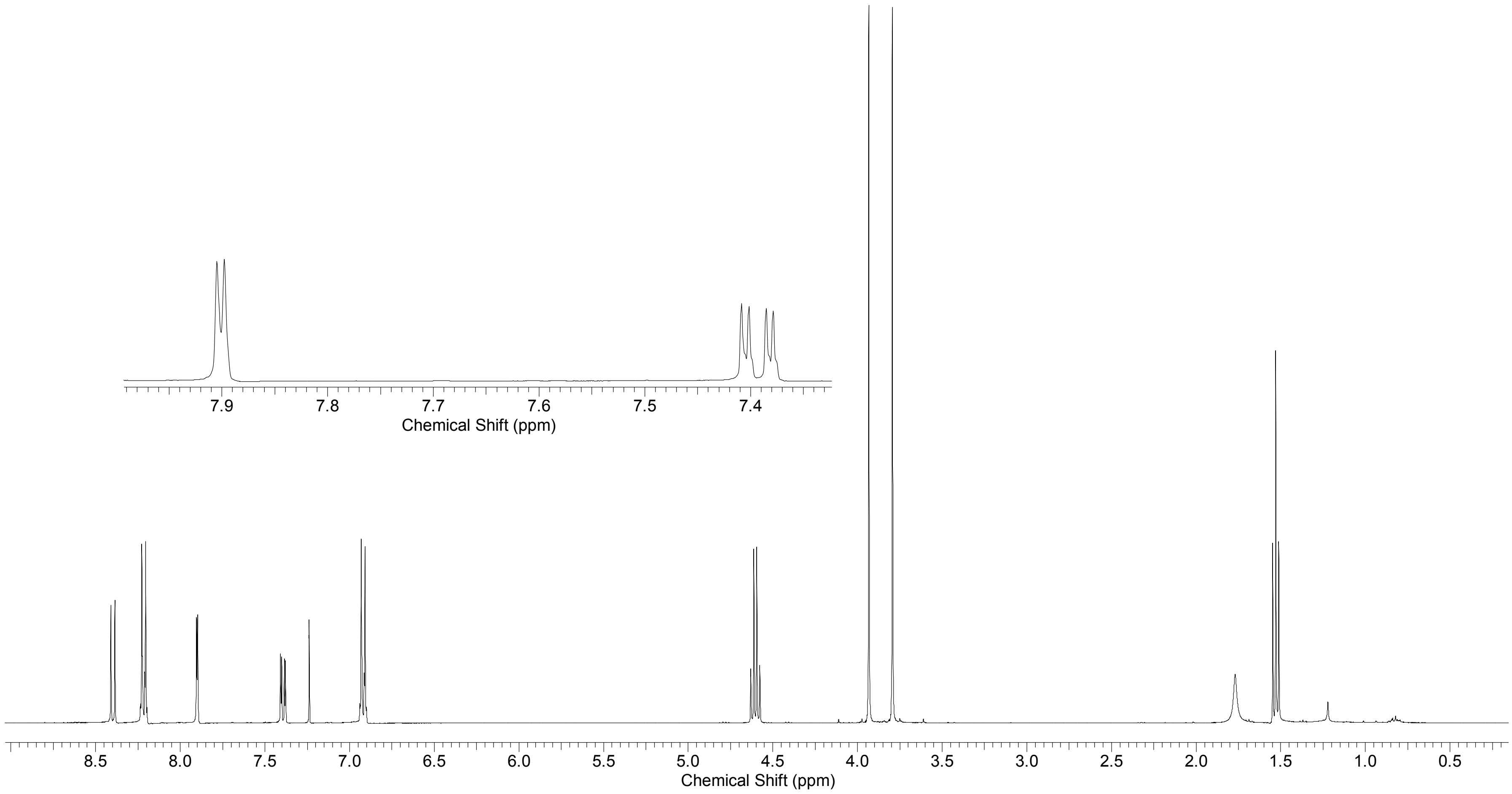
7.9
7.8
7.7
7.6
7.5
7.4
Chemical Shift (ppm)
8.5
8.0
7.5
7.0
6.5
6.0
5.5
5.0
4.5
4.0
3.5
3.0
2.5
2.0
1.5
1.0
0.5
Chemical Shift (ppm)

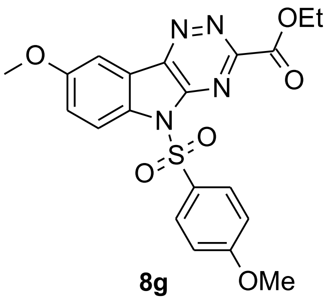
N=N
OEt
O
N
N
O
S
O
O
OMe

8g

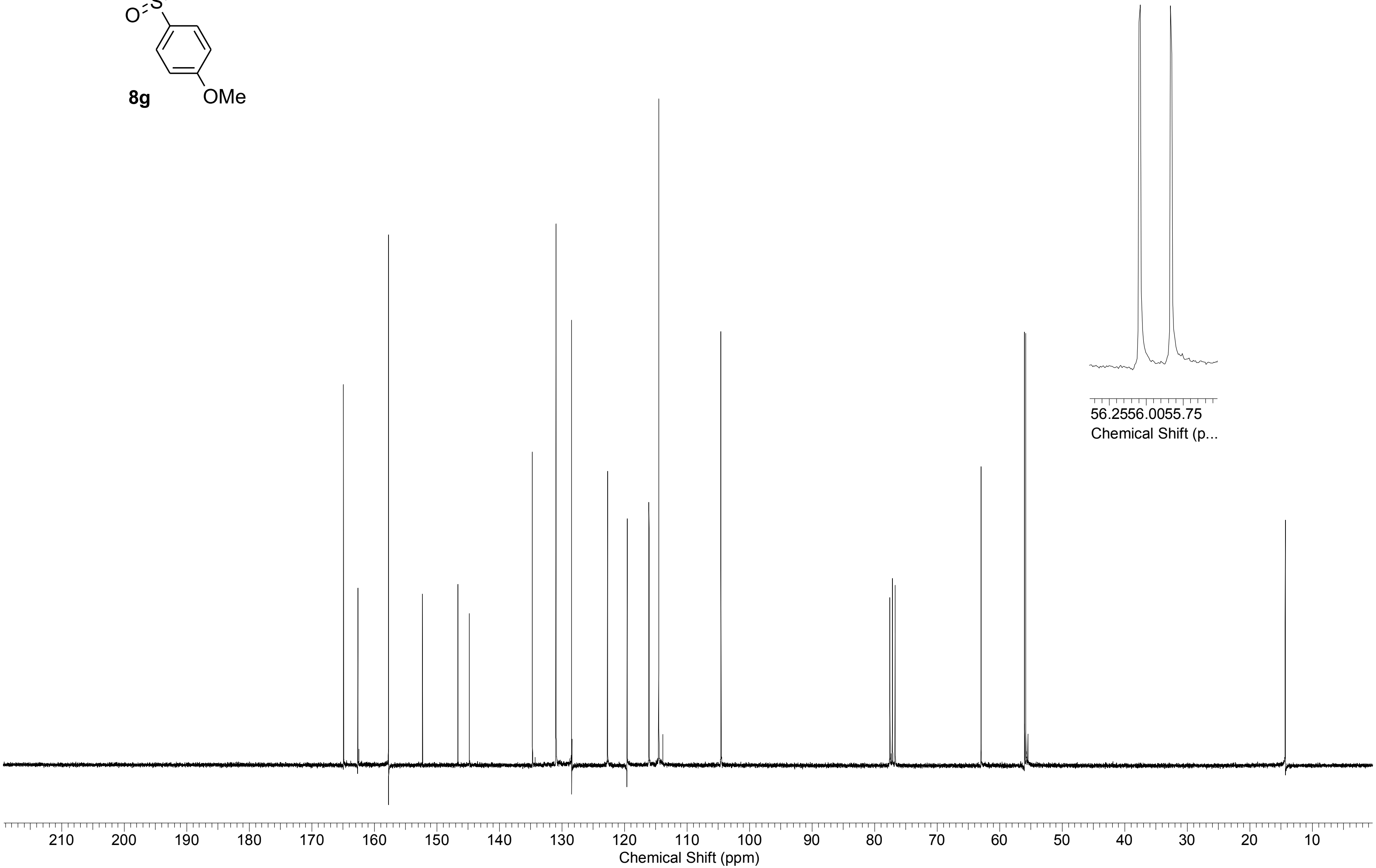
56.2556.0055.75
Chemical Shift (p...
210 200 190 180 170 160 150 140 130 120 110 100 90 80 70 60 50 40 30 20 10
Chemical Shift (ppm)

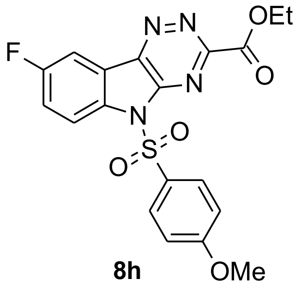

N=N
OEt
F
O
N
N
S
O
O
OMe

8h

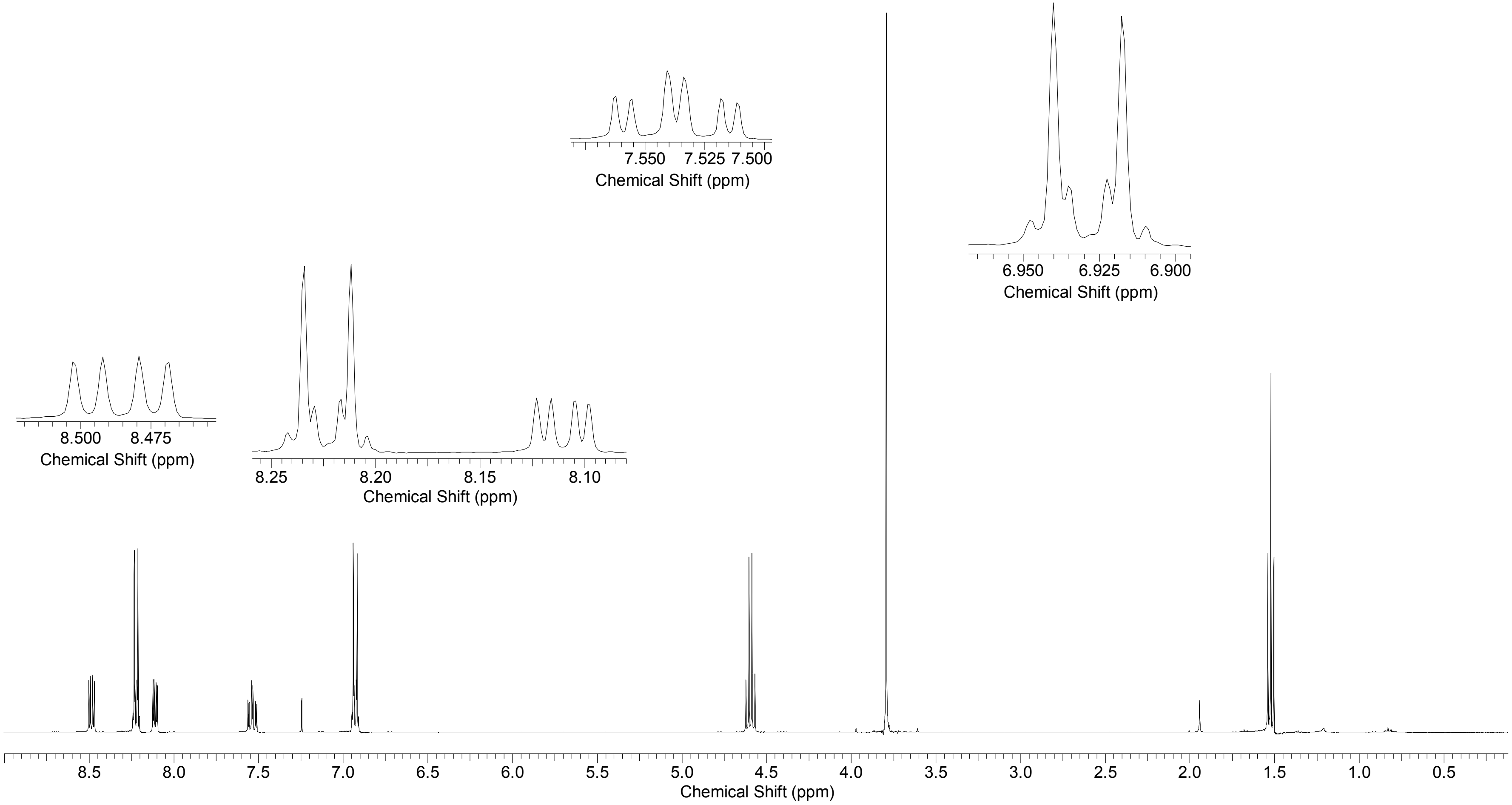

7.550 7.525 7.500
Chemical Shift (ppm)
6.950 6.925 6.900
Chemical Shift (ppm)
8.500 8.475
Chemical Shift (ppm)
8.25 8.20 8.15 8.10
Chemical Shift (ppm)
8.5 8.0 7.5 7.0 6.5 6.0 5.5 5.0 4.5 4.0 3.5 3.0 2.5 2.0 1.5 1.0 0.5
Chemical Shift (ppm)

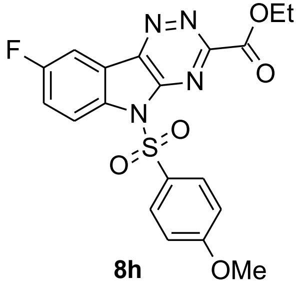

8h

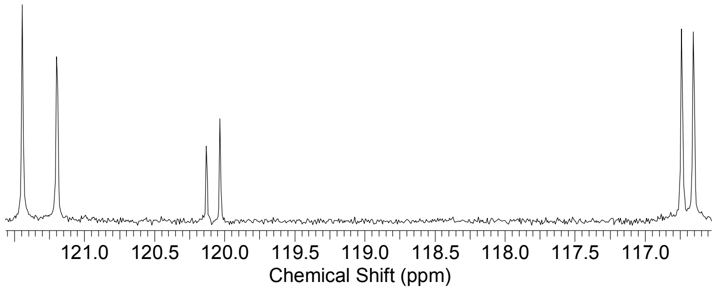

121.0 120.5 120.0 119.5 119.0 118.5 118.0 117.5 117.0
Chemical Shift (ppm)

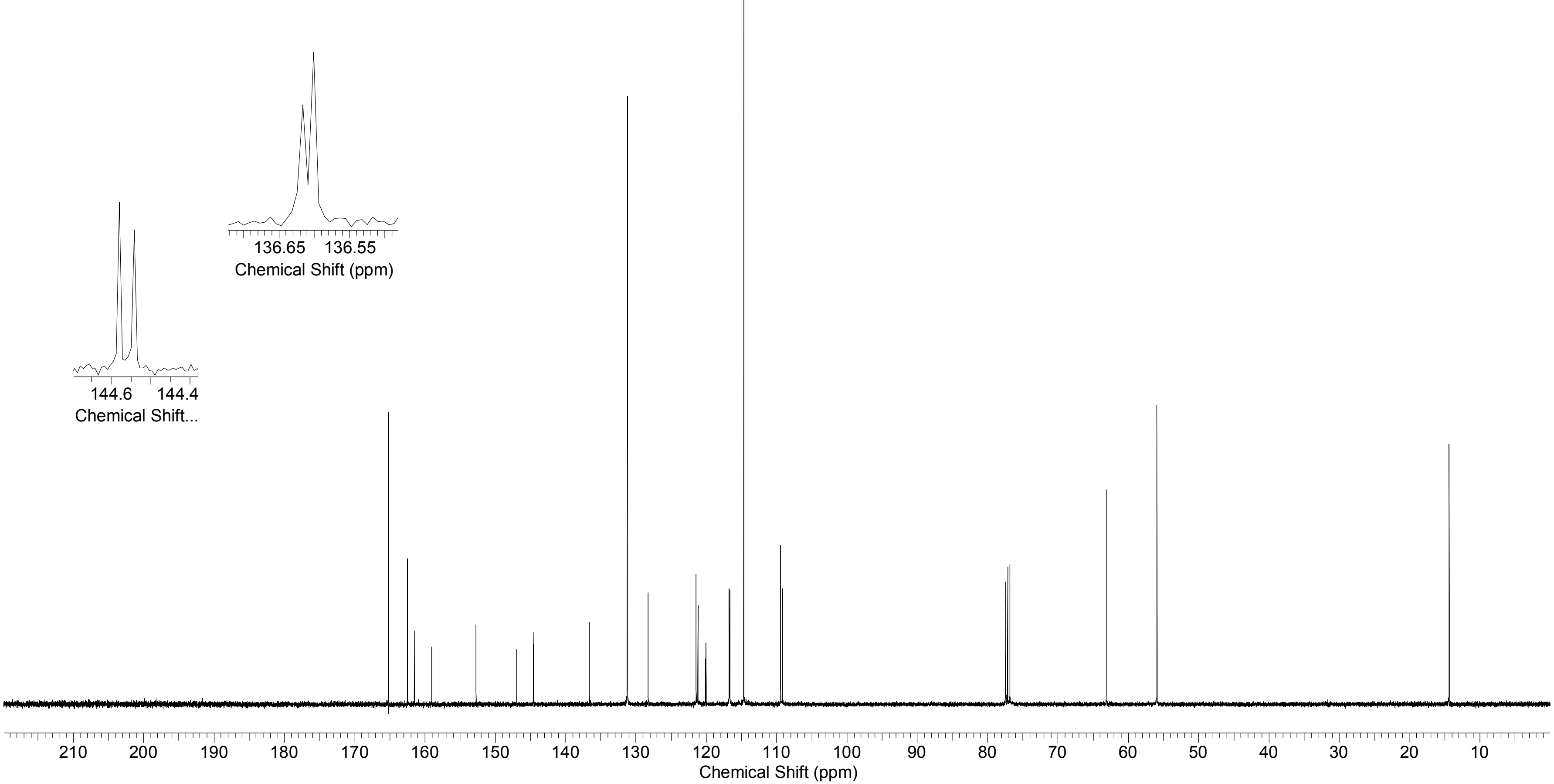

136.65 136.55
Chemical Shift (ppm)
144.6 144.4
Chemical Shift...
210 200 190 180 170 160 150 140 130 120 110 100 90 80 70 60 50 40 30 20 10
Chemical Shift (ppm)

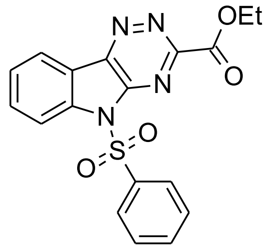

N=N
OEt
O
N
N
O
S
O

8i

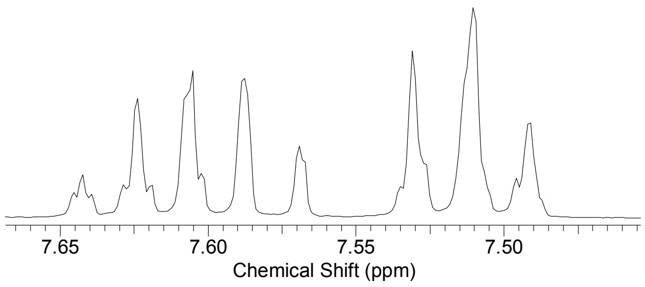

7.65 7.60 7.55 7.50
Chemical Shift (ppm)

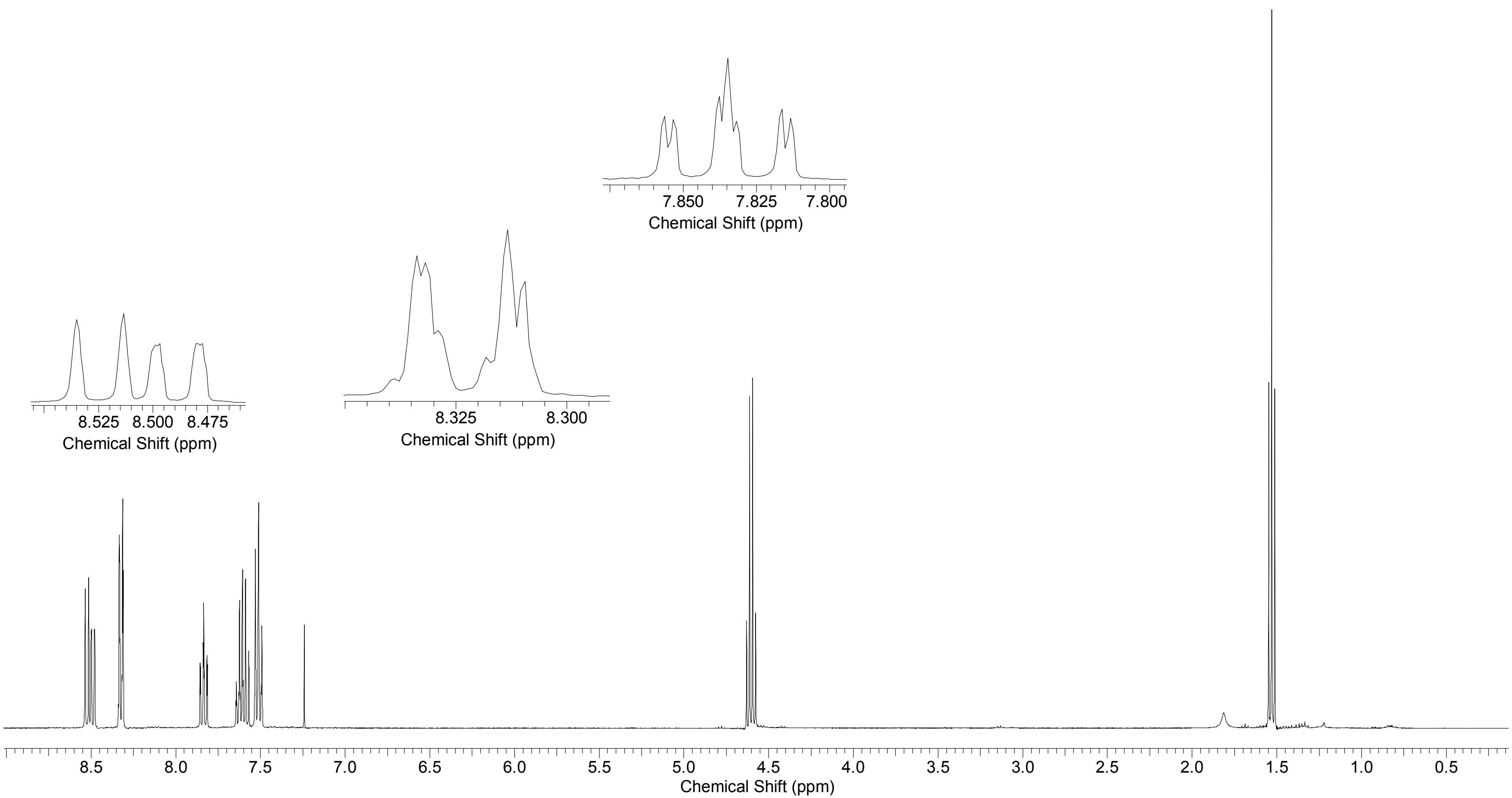

7.850 7.825 7.800
Chemical Shift (ppm)
8.525 8.500 8.475
Chemical Shift (ppm)
8.325 8.300
Chemical Shift (ppm)
8.5 8.0 7.5 7.0 6.5 6.0 5.5 5.0 4.5 4.0 3.5 3.0 2.5 2.0 1.5 1.0 0.5
Chemical Shift (ppm)

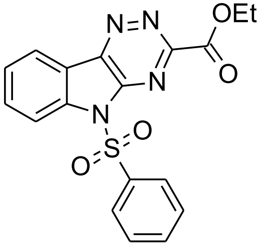

8i

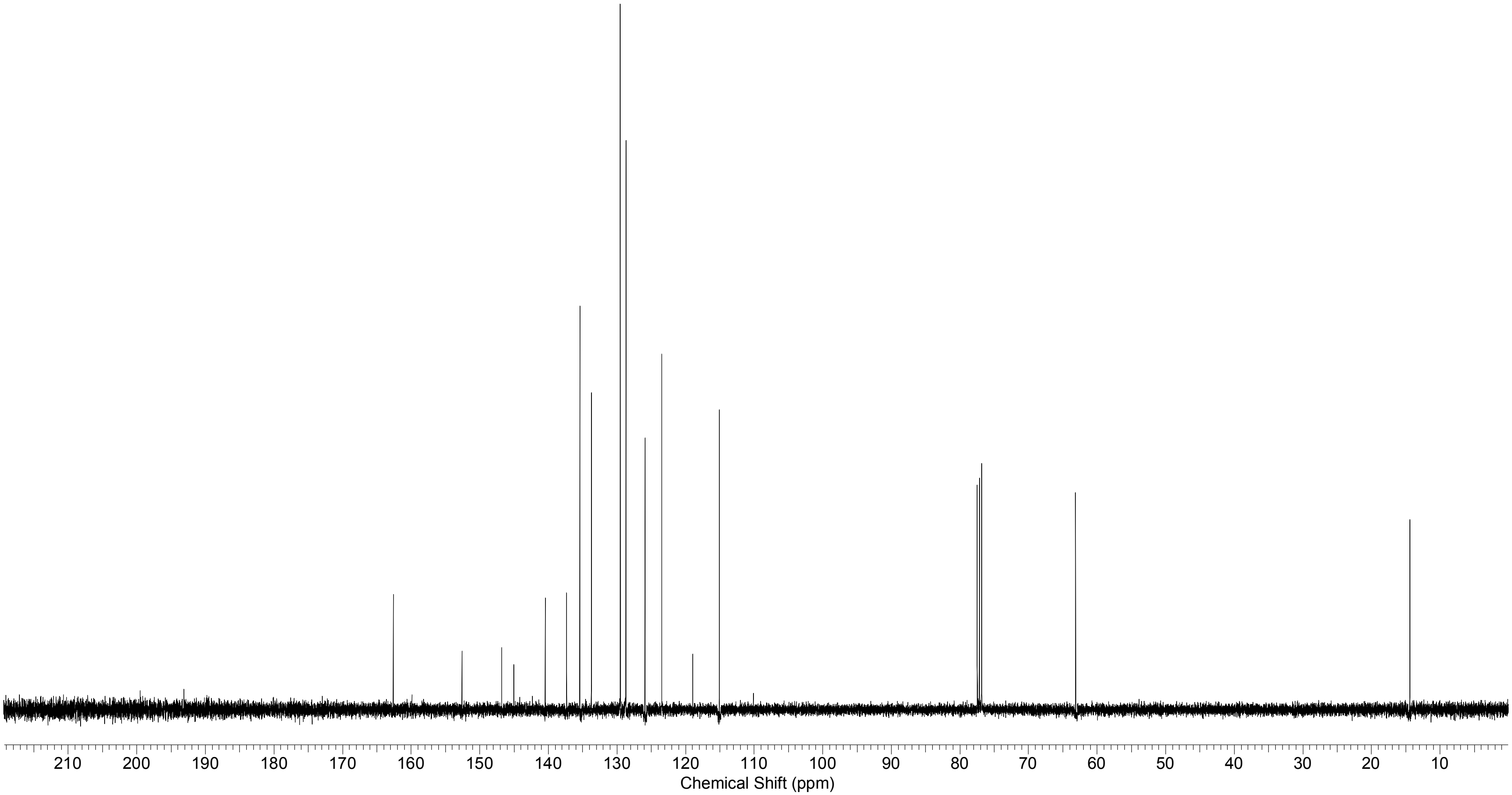

210
200
190
180
170
160
150
140
130
120
110
100
90
80
70
60
50
40
30
20
10
Chemical Shift (ppm)

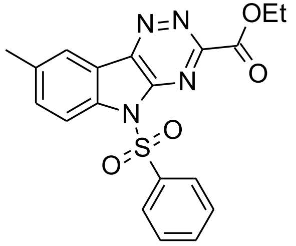
N=N
OEt
O
N
N
N
S
O
O

8j

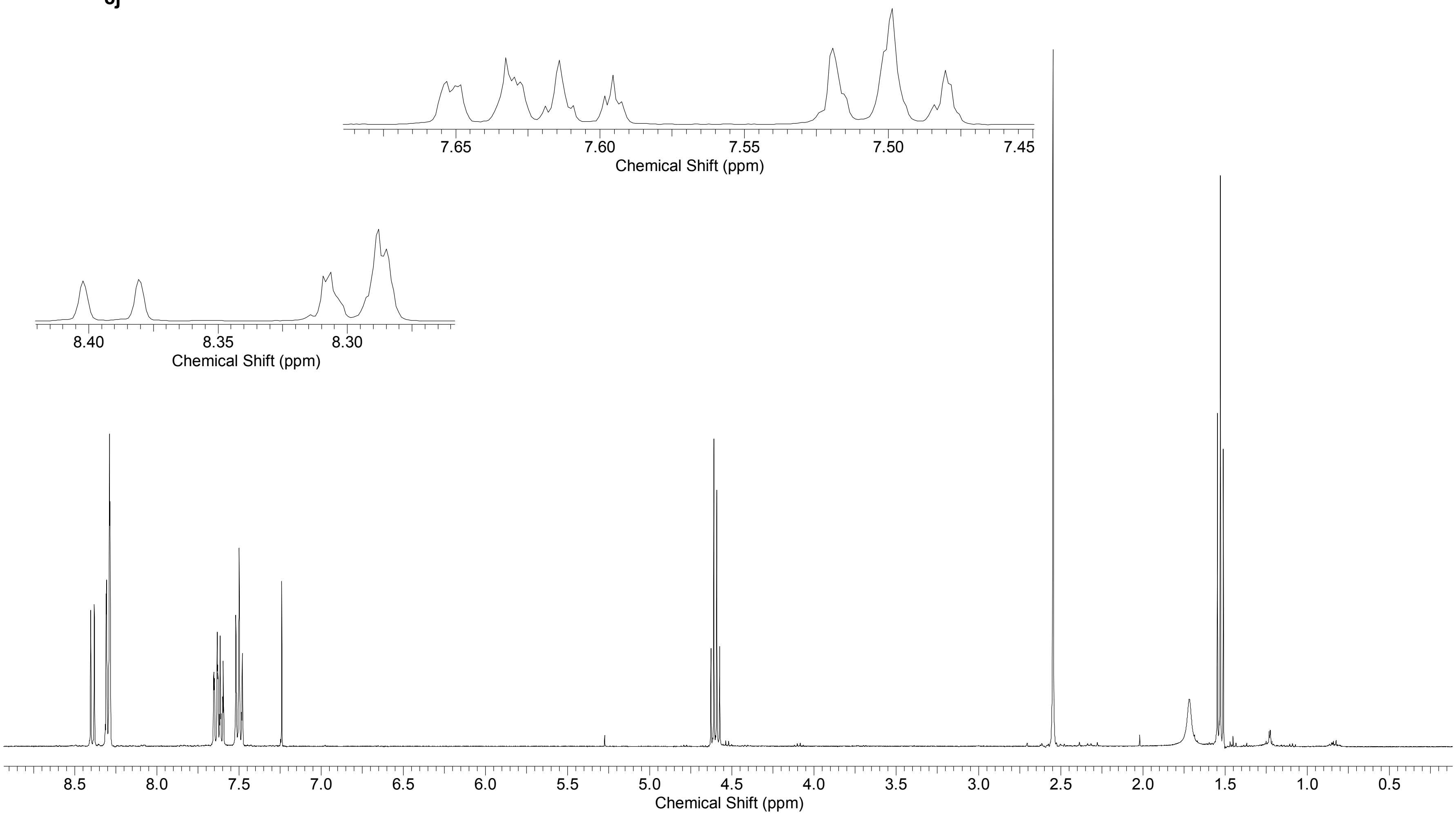
7.65
7.60
7.55
7.50
7.45
Chemical Shift (ppm)
8.40
8.35
8.30
Chemical Shift (ppm)
8.5
8.0
7.5
7.0
6.5
6.0
5.5
5.0
4.5
4.0
3.5
3.0
2.5
2.0
1.5
1.0
0.5
Chemical Shift (ppm)

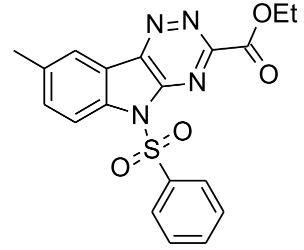
N=N
OEt
O
N
N
S
O
O

8j

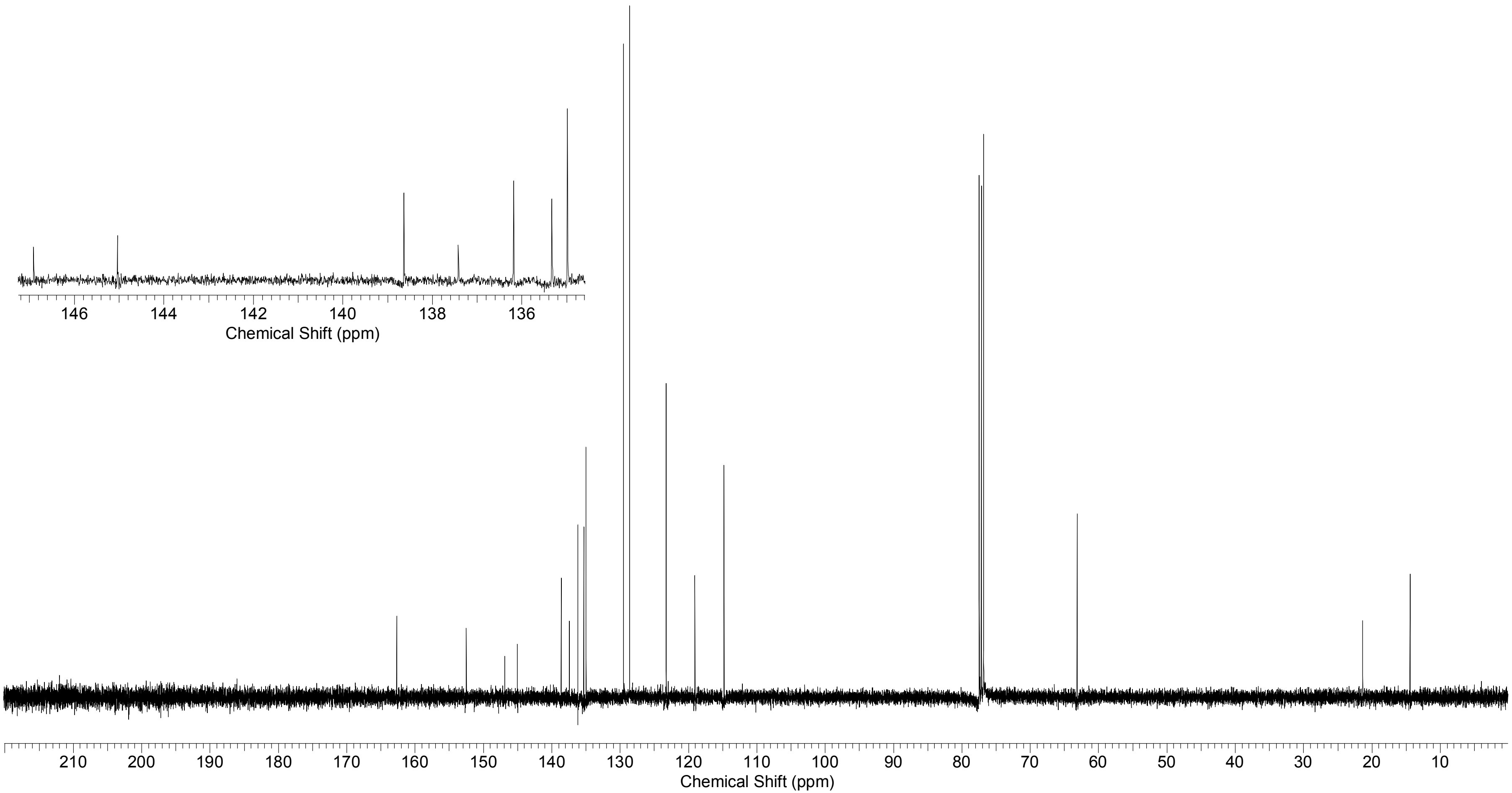
146
144
142
140
138
136
Chemical Shift (ppm)
210
200
190
180
170
160
150
140
130
120
110
100
90
80
70
60
50
40
30
20
10
Chemical Shift (ppm)

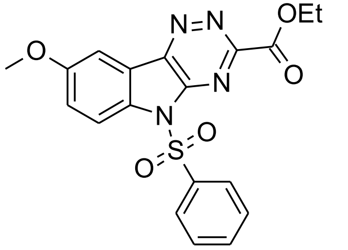

8k

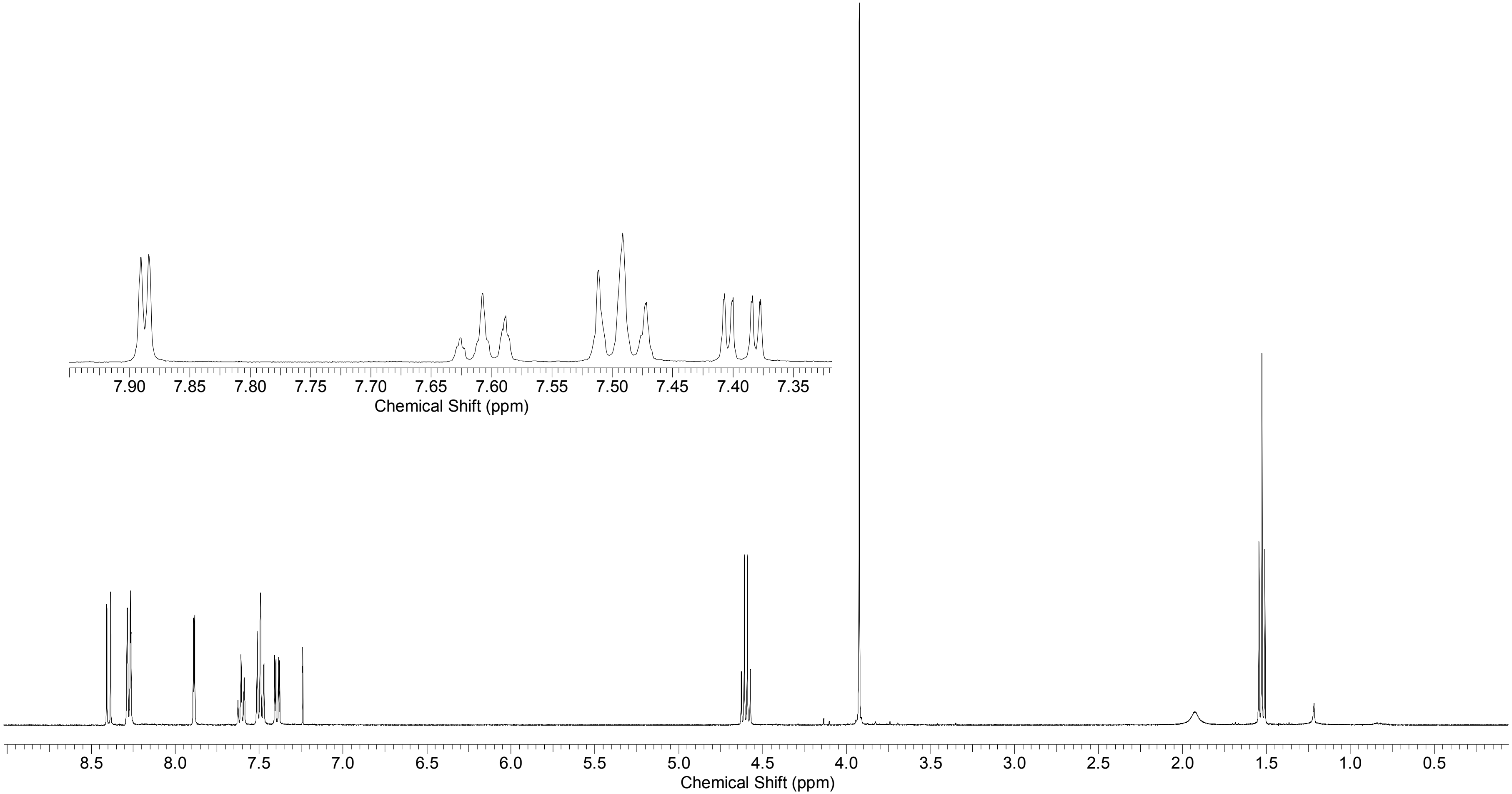

7.90 7.85 7.80 7.75 7.70 7.65 7.60 7.55 7.50 7.45 7.40 7.35
Chemical Shift (ppm)
8.5 8.0 7.5 7.0 6.5 6.0 5.5 5.0 4.5 4.0 3.5 3.0 2.5 2.0 1.5 1.0 0.5
Chemical Shift (ppm)

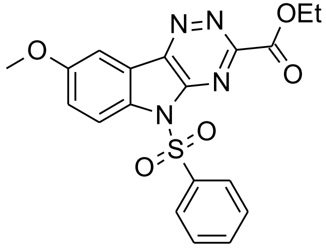

8k

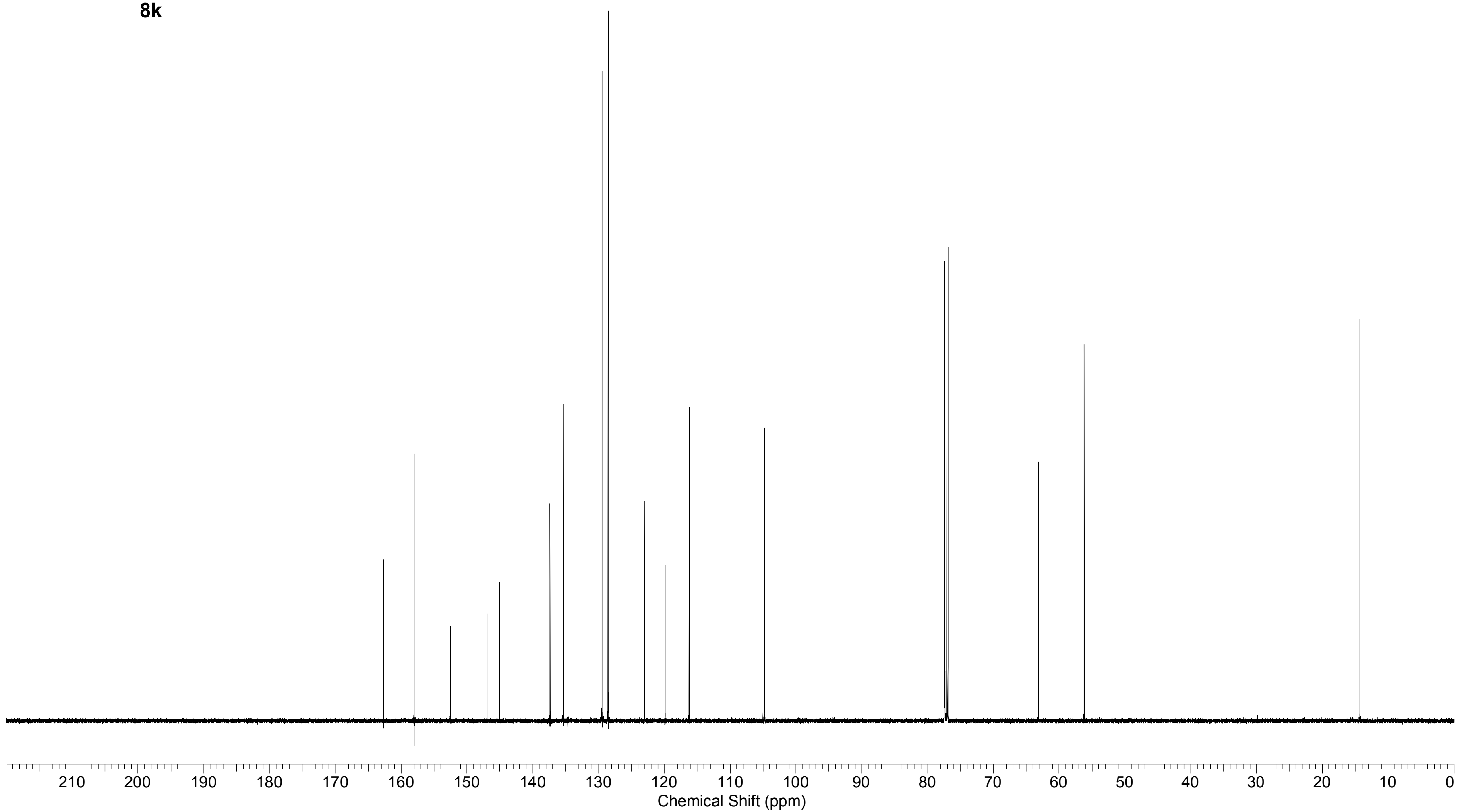

210 200 190 180 170 160 150 140 130 120 110 100 90 80 70 60 50 40 30 20 10 0
Chemical Shift (ppm)

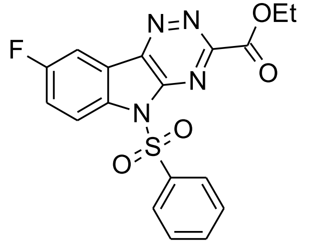
F
N=N
OEt
O
N
N
O
S
O

8l

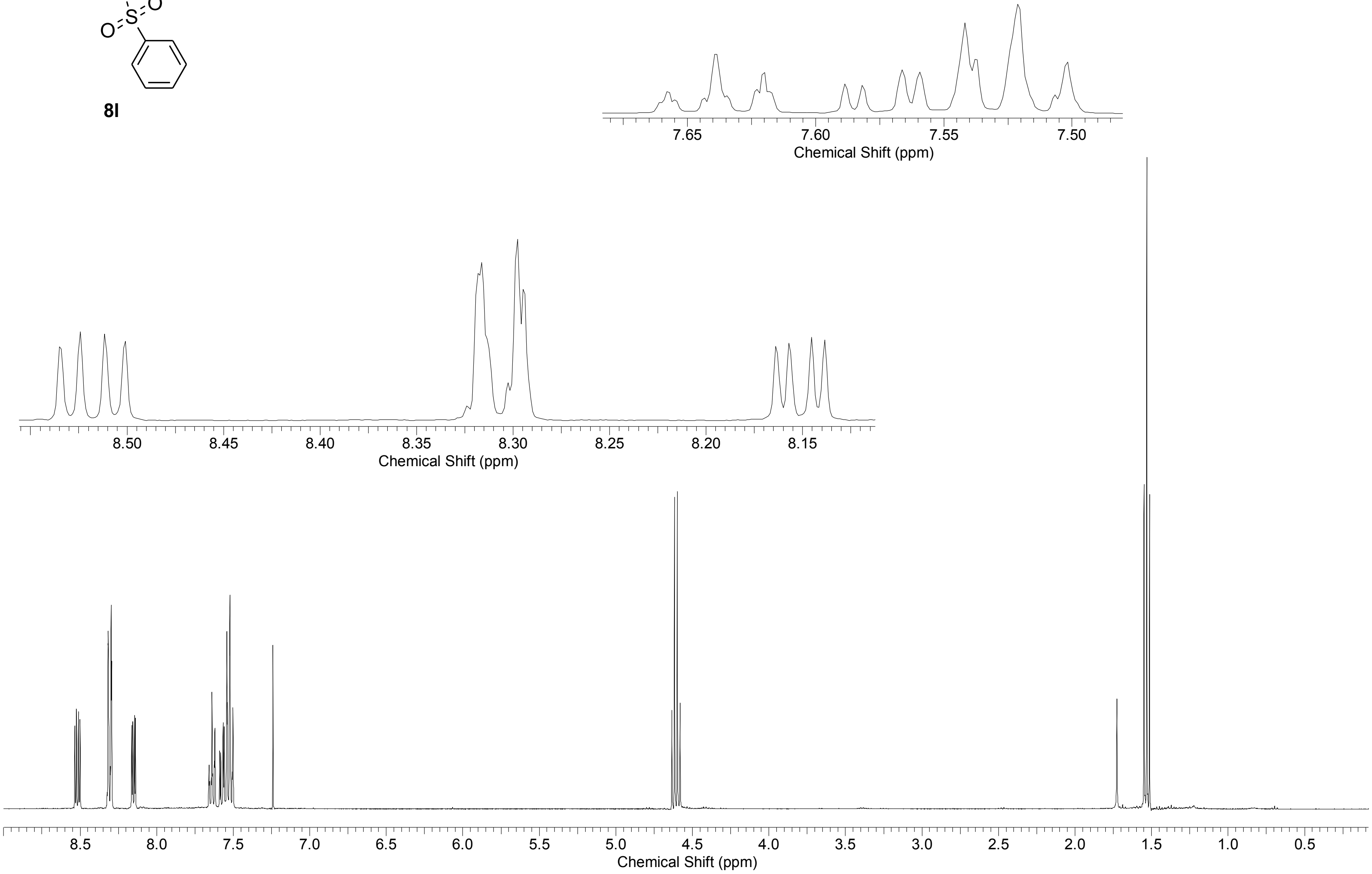
7.65
7.60
7.55
7.50
Chemical Shift (ppm)
8.50
8.45
8.40
8.35
8.30
8.25
8.20
8.15
Chemical Shift (ppm)
8.5
8.0
7.5
7.0
6.5
6.0
5.5
5.0
4.5
4.0
3.5
3.0
2.5
2.0
1.5
1.0
0.5
Chemical Shift (ppm)

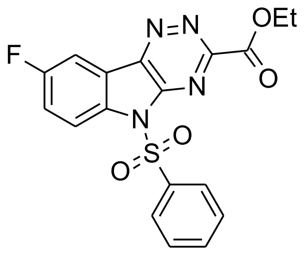

8l

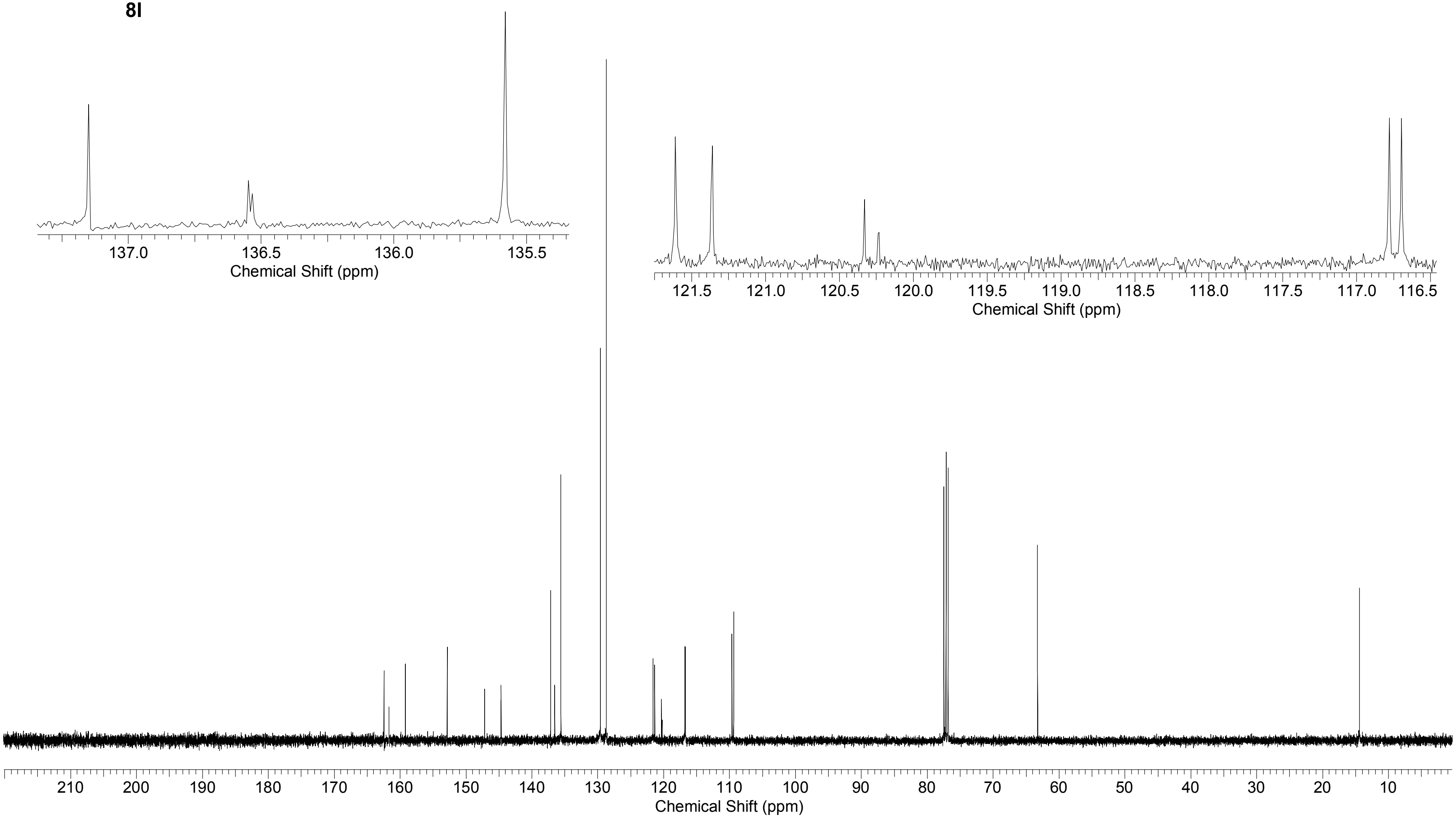

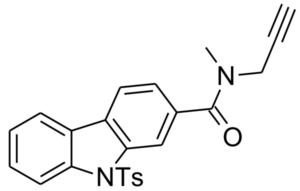
NTs
O
N

13a

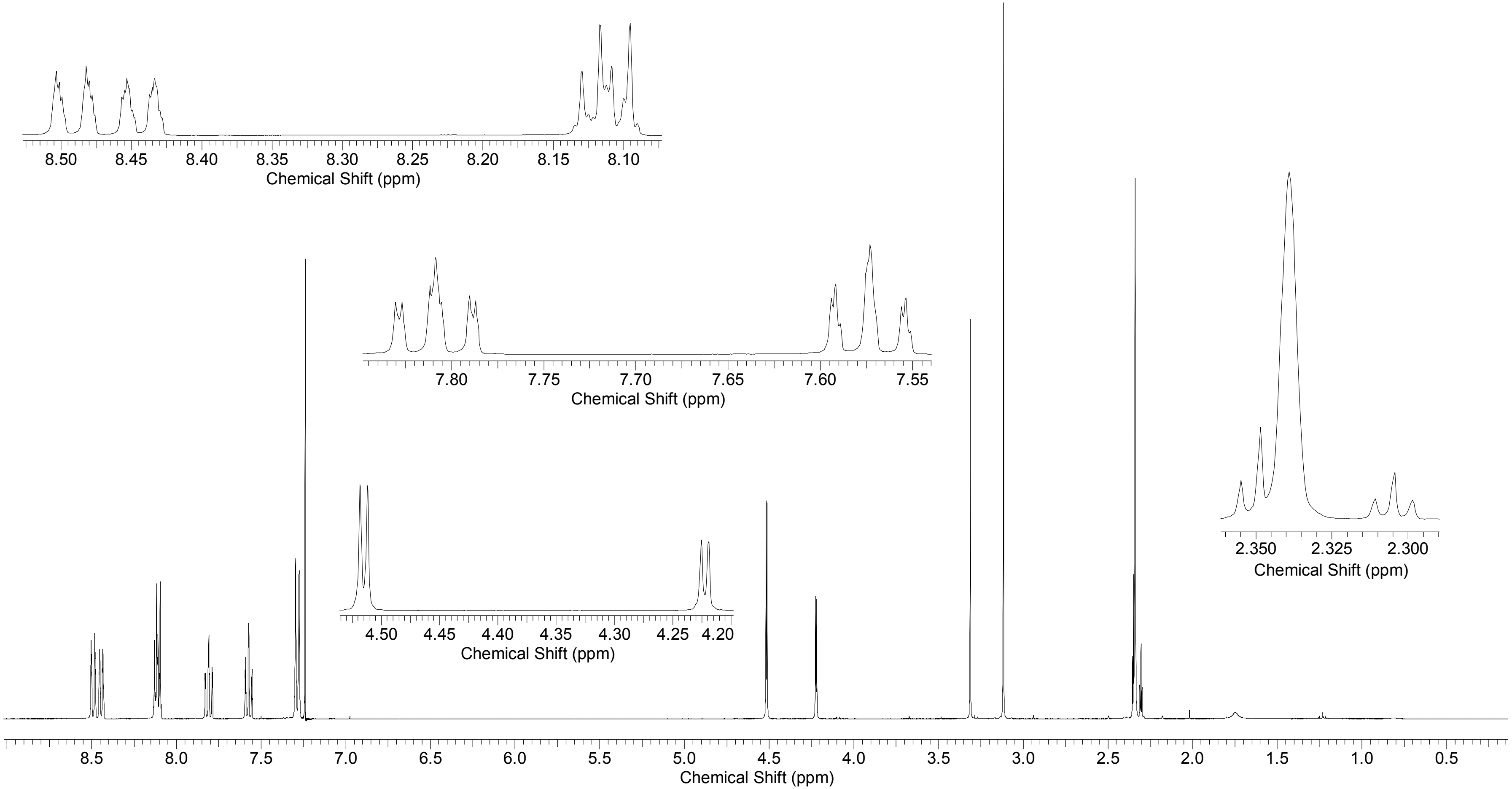
8.50 8.45 8.40 8.35 8.30 8.25 8.20 8.15 8.10
Chemical Shift (ppm)
7.80 7.75 7.70 7.65 7.60 7.55
Chemical Shift (ppm)
2.350 2.325 2.300
Chemical Shift (ppm)
4.50 4.45 4.40 4.35 4.30 4.25 4.20
Chemical Shift (ppm)
8.5 8.0 7.5 7.0 6.5 6.0 5.5 5.0 4.5 4.0 3.5 3.0 2.5 2.0 1.5 1.0 0.5
Chemical Shift (ppm)

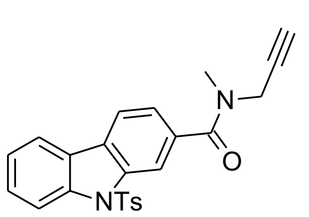
N
O
NTs

13a

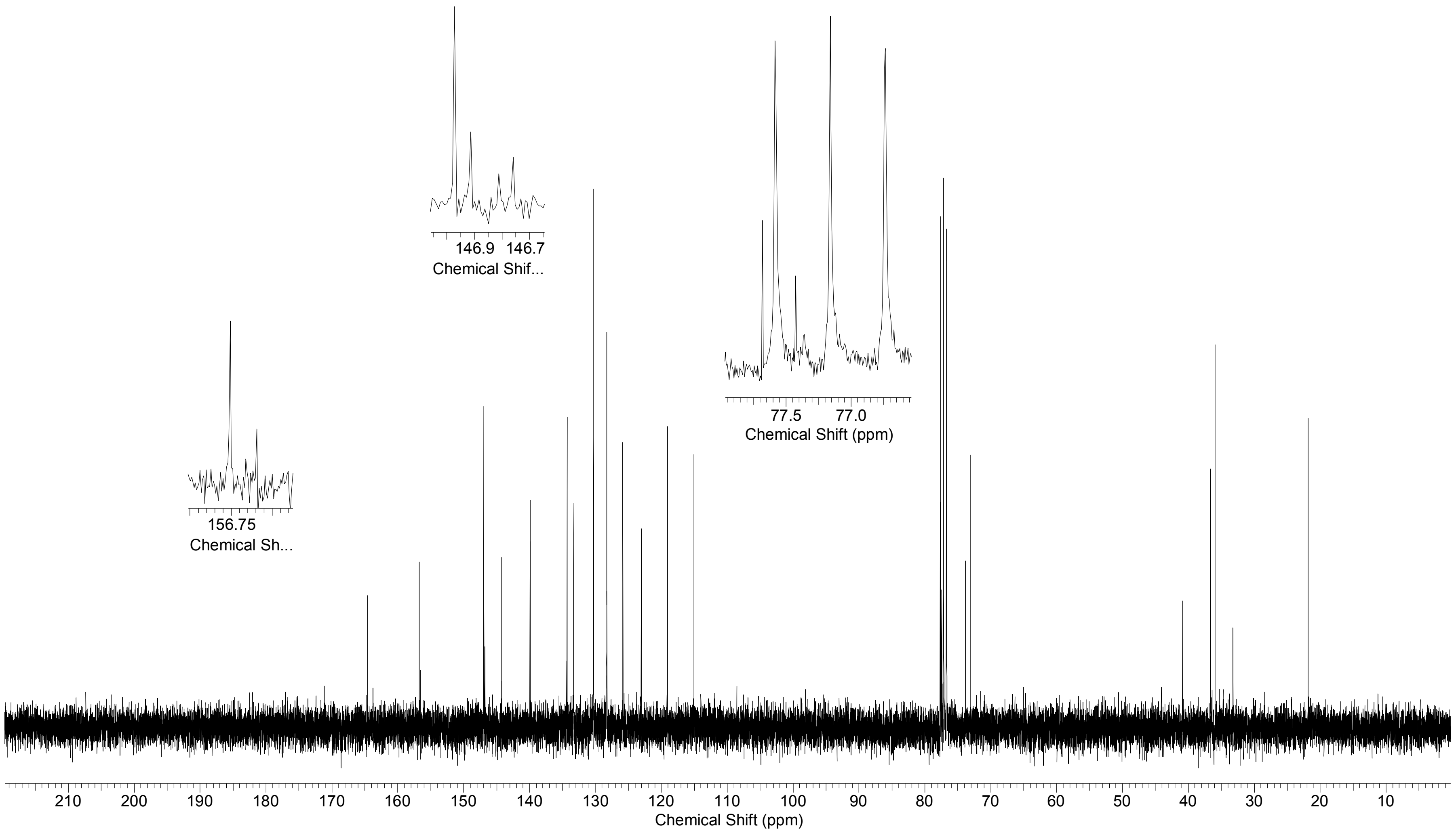
146.9 146.7
Chemical Shif...
77.5 77.0
Chemical Shift (ppm)
156.75
Chemical Sh...
210 200 190 180 170 160 150 140 130 120 110 100 90 80 70 60 50 40 30 20 10
Chemical Shift (ppm)

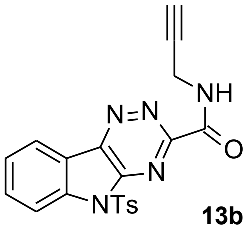

NH
N=N
O
N
NTs

13b

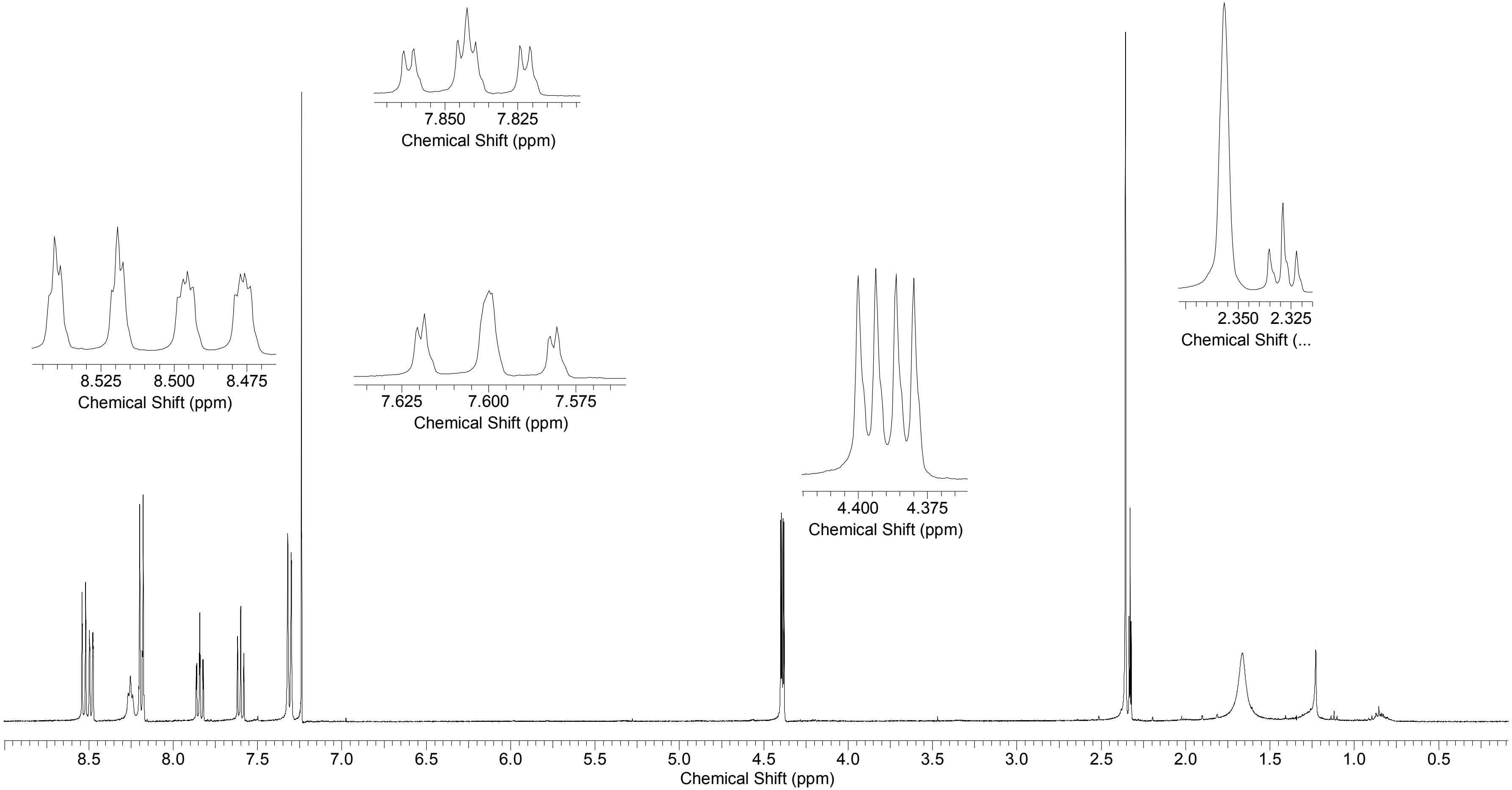

7.850
7.825
Chemical Shift (ppm)
8.525
8.500
8.475
Chemical Shift (ppm)
7.625
7.600
7.575
Chemical Shift (ppm)
4.400
4.375
Chemical Shift (ppm)
2.350
2.325
Chemical Shift (...
8.5
8.0
7.5
7.0
6.5
6.0
5.5
5.0
4.5
4.0
3.5
3.0
2.5
2.0
1.5
1.0
0.5
Chemical Shift (ppm)

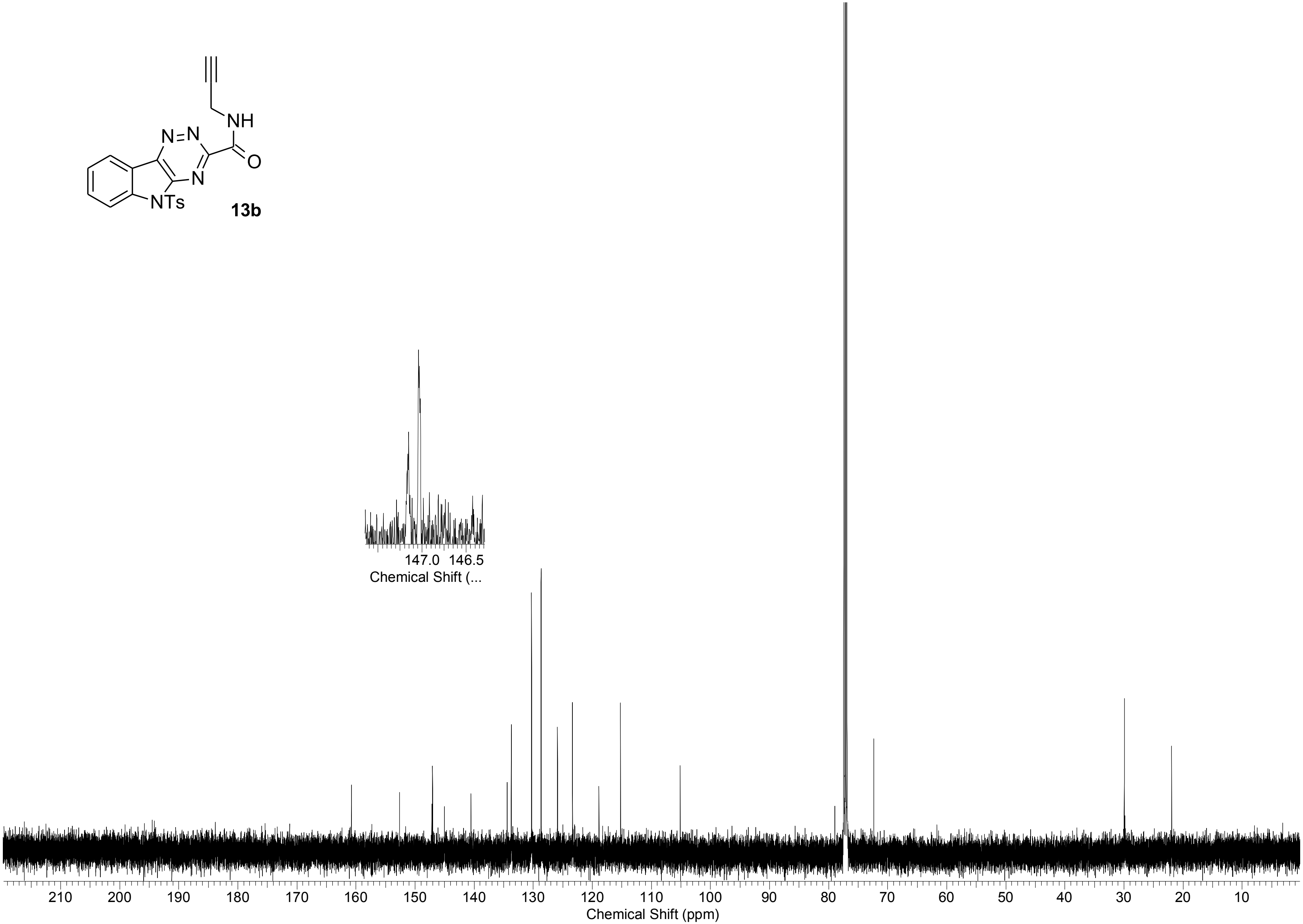
NH
N=N
N
O
NTs
13b
147.0 146.5
Chemical Shift (...
210 200 190 180 170 160 150 140 130 120 110 100 90 80 70 60 50 40 30 20 10
Chemical Shift (ppm)

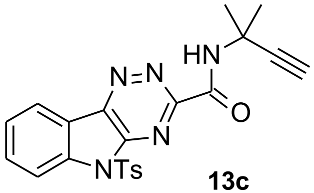
HN
N=N
N
O
NTs

13c

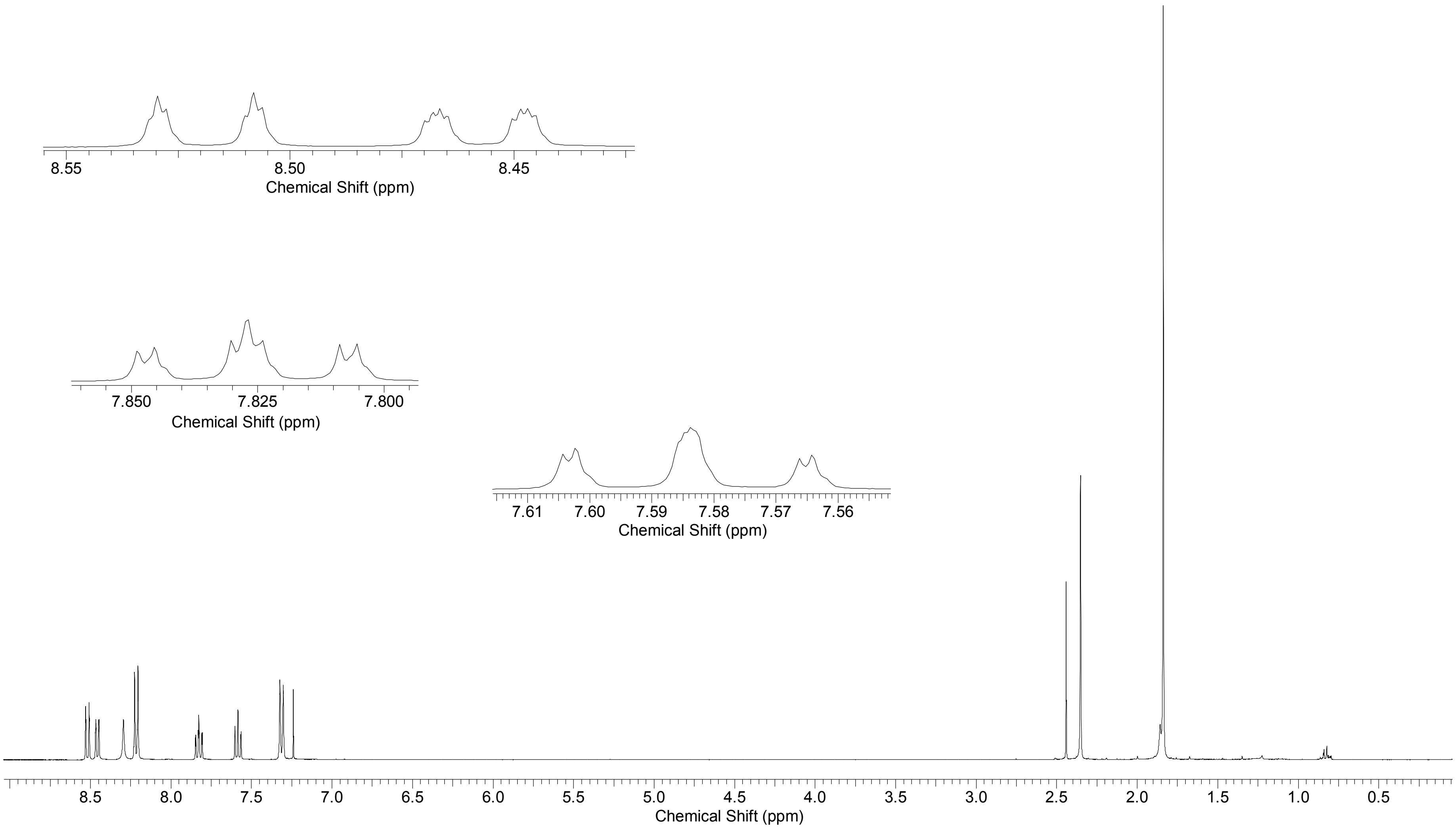
8.55
8.50
8.45
Chemical Shift (ppm)
7.850
7.825
7.800
Chemical Shift (ppm)
7.61
7.60
7.59
7.58
7.57
7.56
Chemical Shift (ppm)
8.5
8.0
7.5
7.0
6.5
6.0
5.5
5.0
4.5
4.0
3.5
3.0
2.5
2.0
1.5
1.0
0.5
Chemical Shift (ppm)

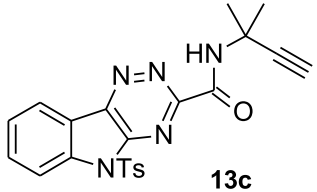
HN
N=N
O
N
NTs

13c

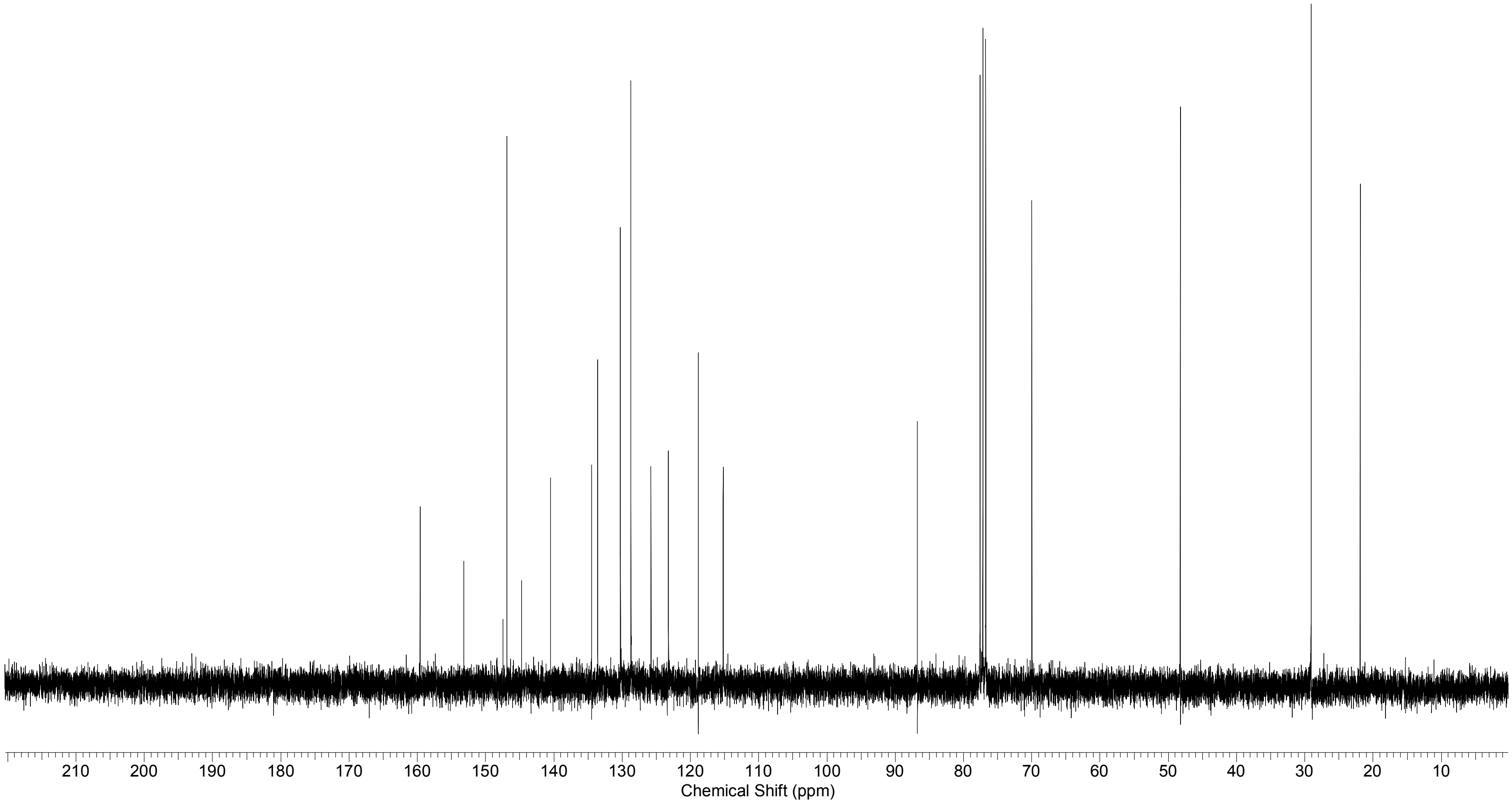
210
200
190
180
170
160
150
140
130
120
110
100
90
80
70
60
50
40
30
20
10
Chemical Shift (ppm)

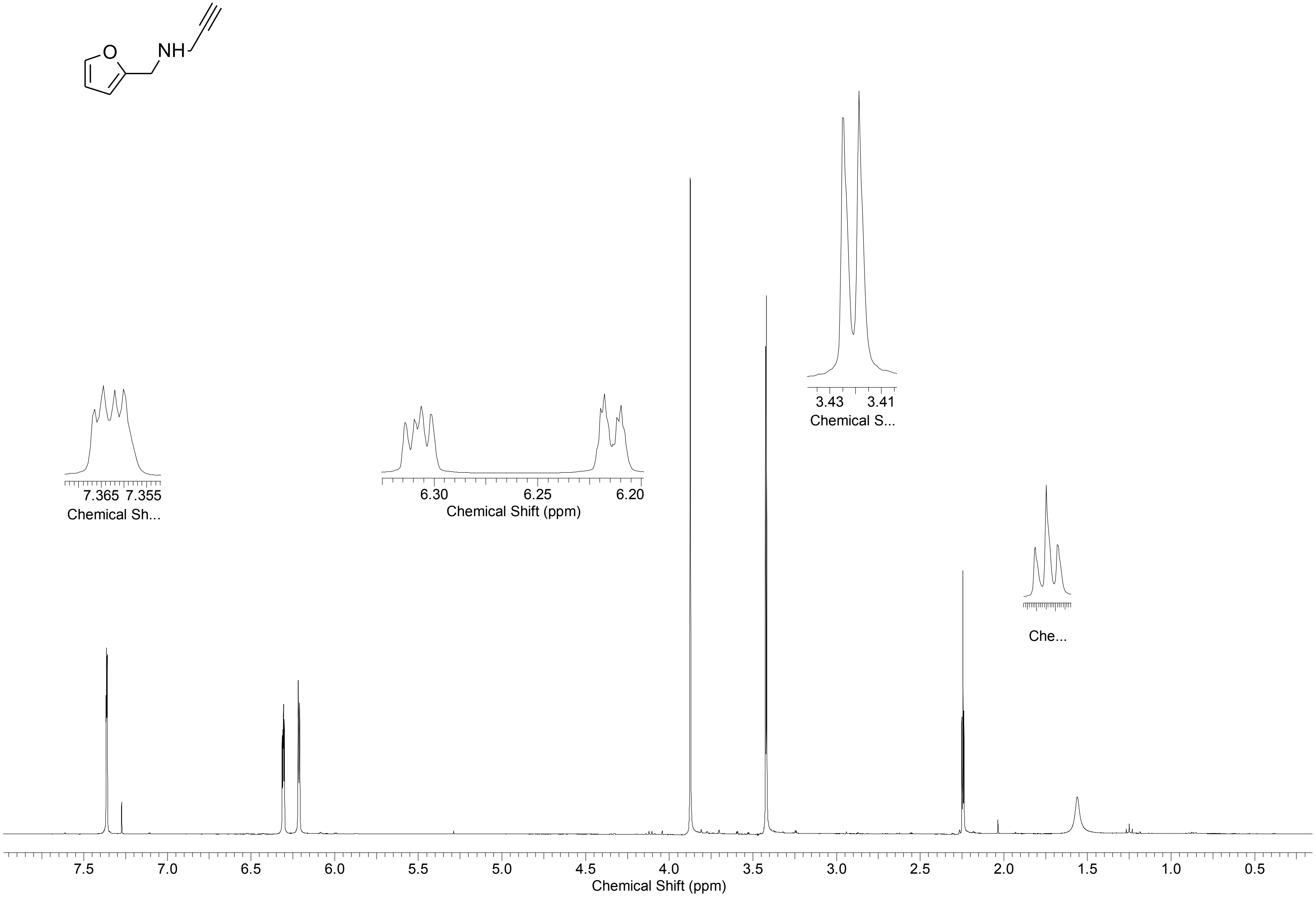
O
NH
7.365 7.355
Chemical Sh...
6.30
6.25
6.20
Chemical Shift (ppm)
3.43
3.41
Chemical S...
Che...
7.5
7.0
6.5
6.0
5.5
5.0
4.5
4.0
3.5
3.0
2.5
2.0
1.5
1.0
0.5
Chemical Shift (ppm)

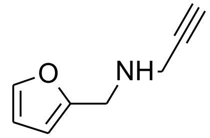
O
NH

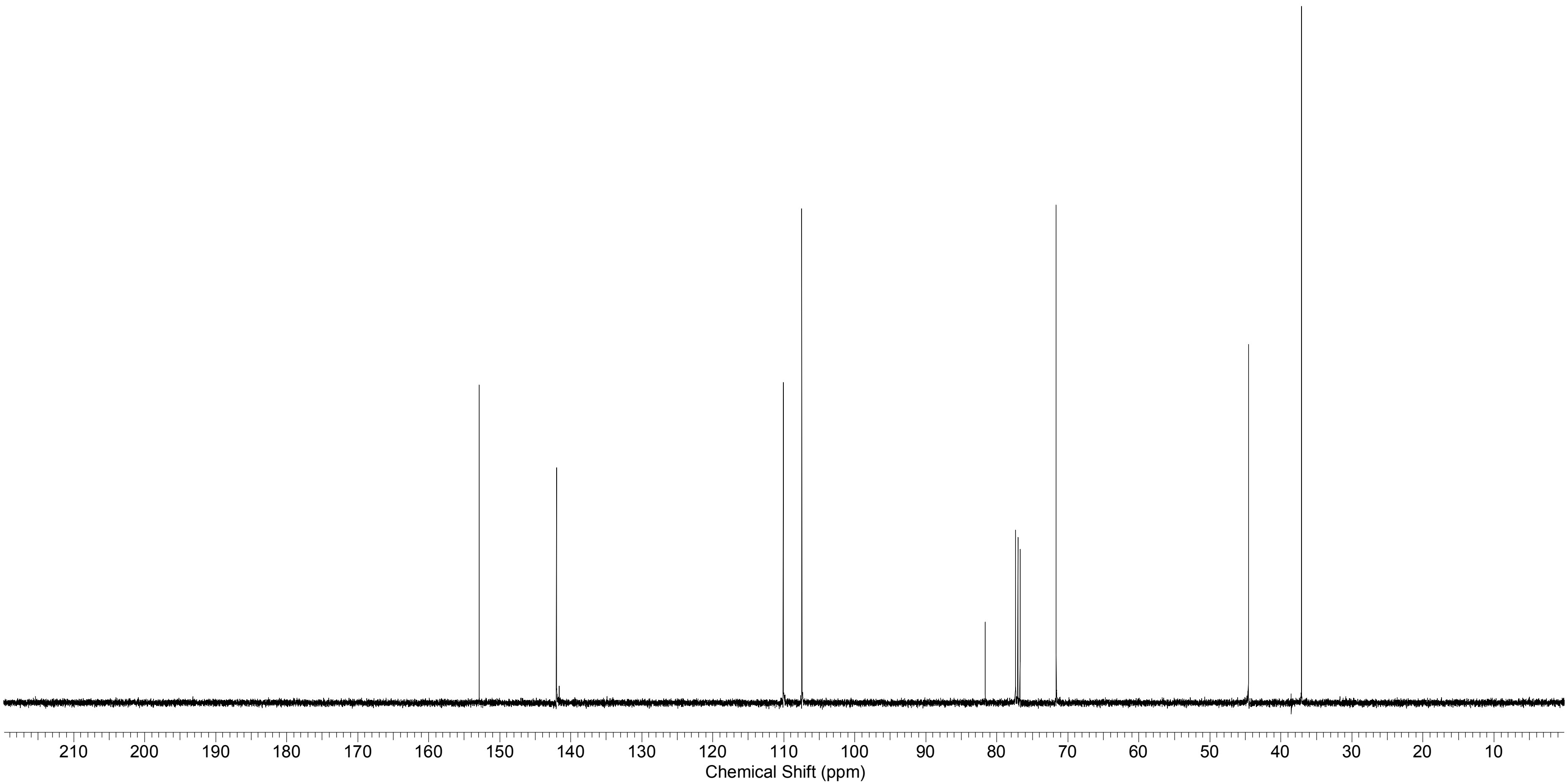
210 200 190 180 170 160 150 140 130 120 110 100 90 80 70 60 50 40 30 20 10
Chemical Shift (ppm)

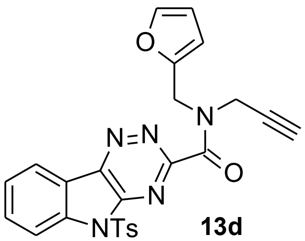
N=N
N
O
NTs
O

13d

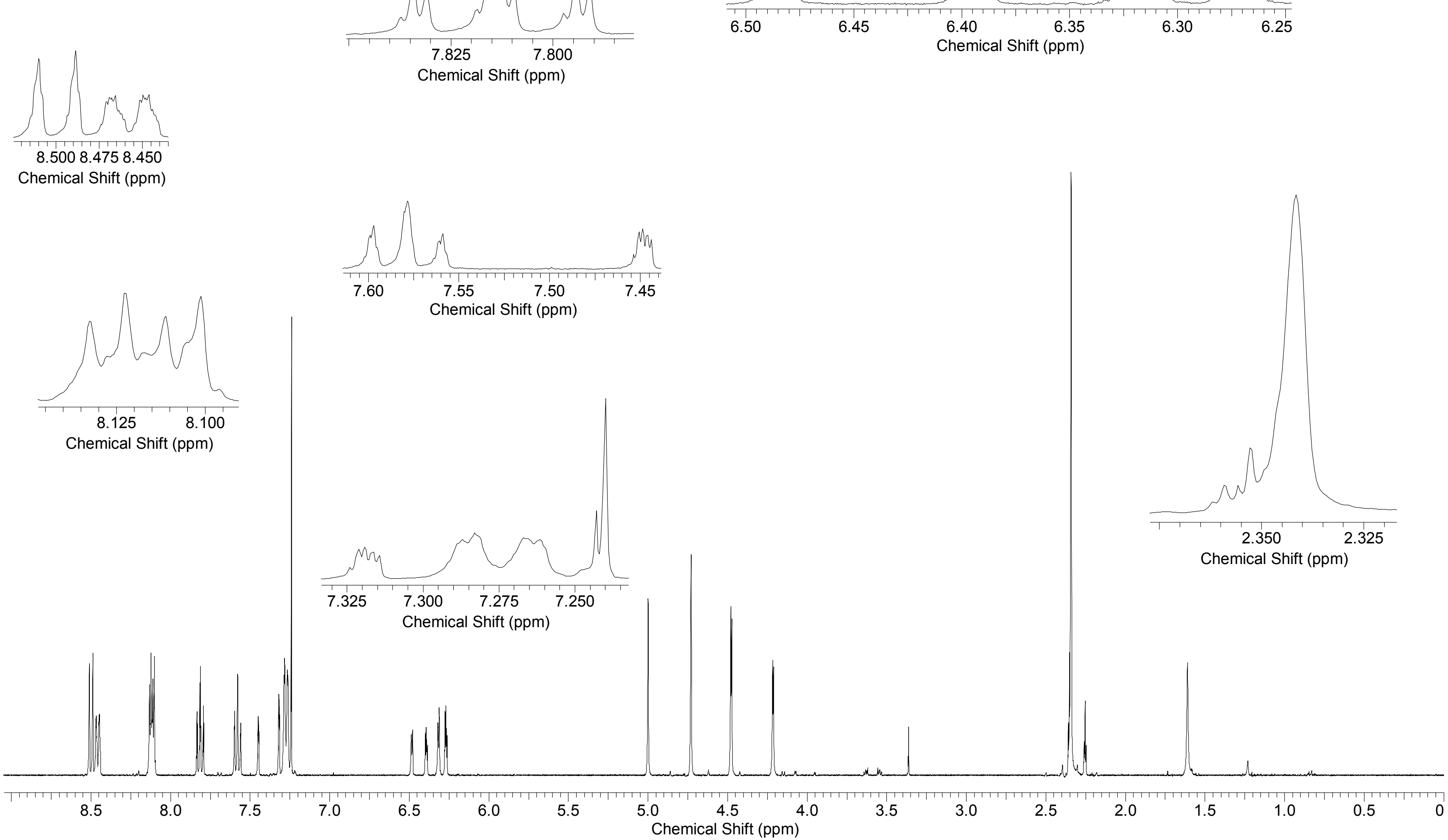
8.500 8.475 8.450
Chemical Shift (ppm)
7.825 7.800
Chemical Shift (ppm)
6.50 6.45 6.40 6.35 6.30 6.25
Chemical Shift (ppm)
8.125 8.100
Chemical Shift (ppm)
7.60 7.55 7.50 7.45
Chemical Shift (ppm)
7.325 7.300 7.275 7.250
Chemical Shift (ppm)
2.350 2.325
Chemical Shift (ppm)
8.5 8.0 7.5 7.0 6.5 6.0 5.5 5.0 4.5 4.0 3.5 3.0 2.5 2.0 1.5 1.0 0.5 0
Chemical Shift (ppm)

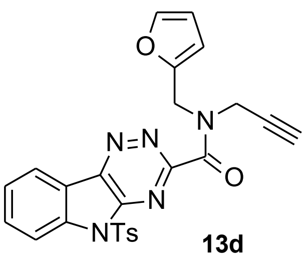
O
N
N=N
N
O
NTs

13d

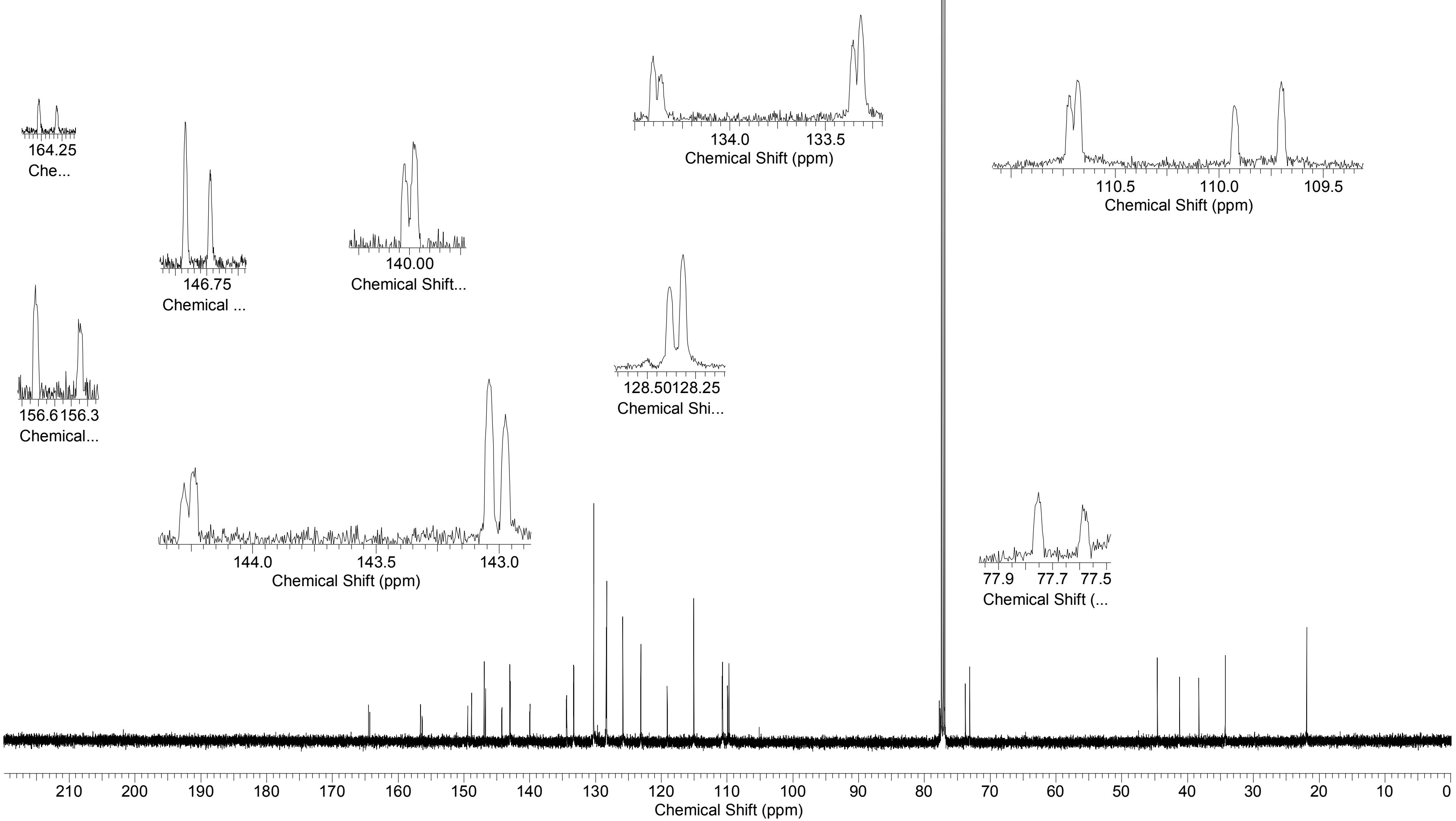
164.25
Che...
146.75
Chemical ...
140.00
Chemical Shift...
156.6 156.3
Chemical...
144.0 143.5 143.0
Chemical Shift (ppm)
134.0 133.5
Chemical Shift (ppm)
128.50128.25
Chemical Shi...
110.5 110.0 109.5
Chemical Shift (ppm)
77.9 77.7 77.5
Chemical Shift (...
210 200 190 180 170 160 150 140 130 120 110 100 90 80 70 60 50 40 30 20 10 0
Chemical Shift (ppm)

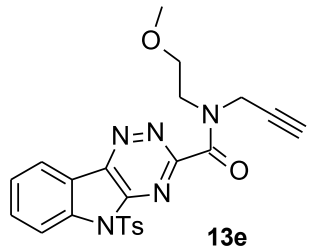
N
O
N=N
N
NTs

13e

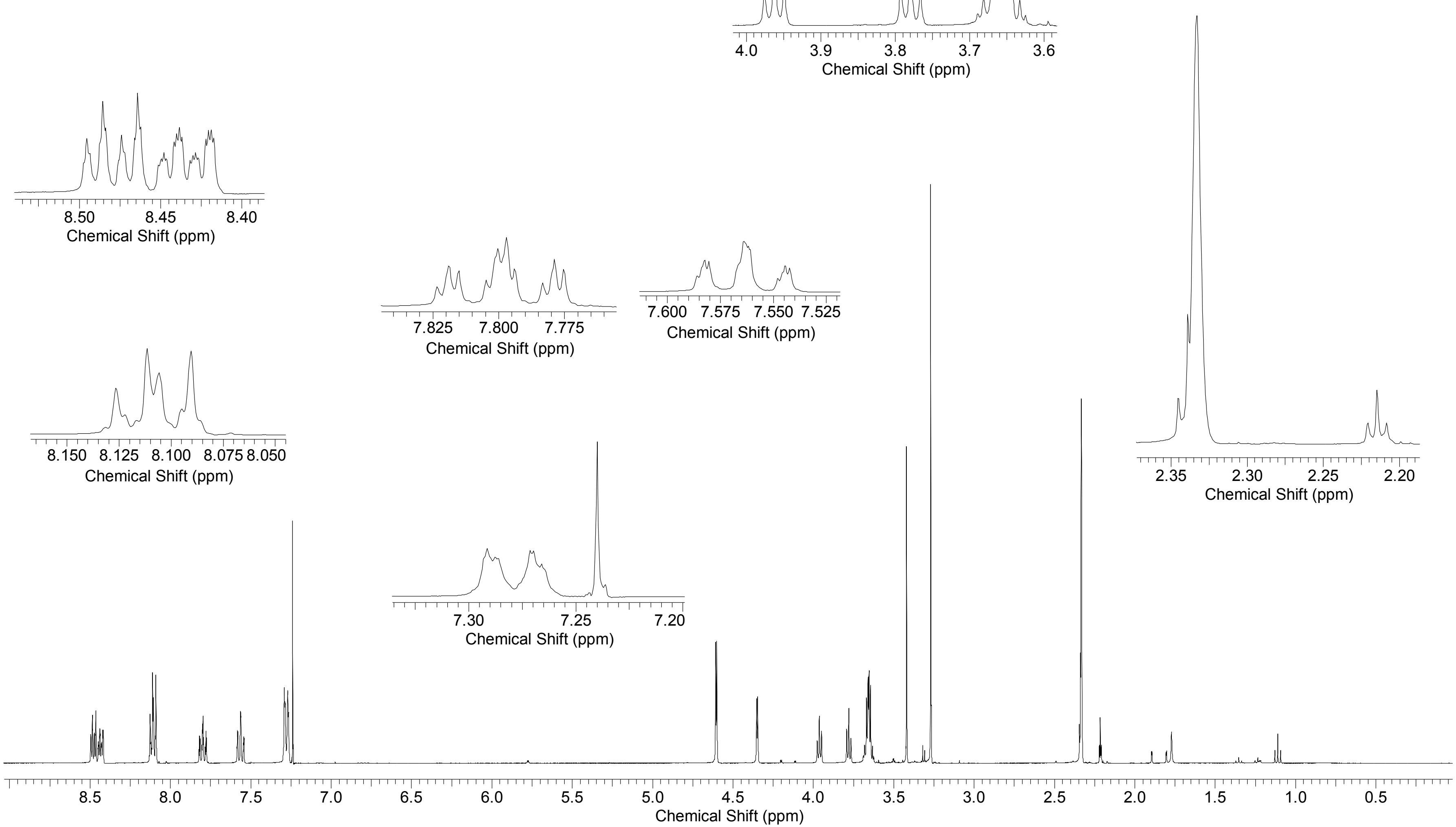
4.0 3.9 3.8 3.7 3.6
Chemical Shift (ppm)
8.50 8.45 8.40
Chemical Shift (ppm)
7.825 7.800 7.775
Chemical Shift (ppm)
7.600 7.575 7.550 7.525
Chemical Shift (ppm)
8.150 8.125 8.100 8.075 8.050
Chemical Shift (ppm)
2.35 2.30 2.25 2.20
Chemical Shift (ppm)
7.30 7.25 7.20
Chemical Shift (ppm)
8.5 8.0 7.5 7.0 6.5 6.0 5.5 5.0 4.5 4.0 3.5 3.0 2.5 2.0 1.5 1.0 0.5
Chemical Shift (ppm)

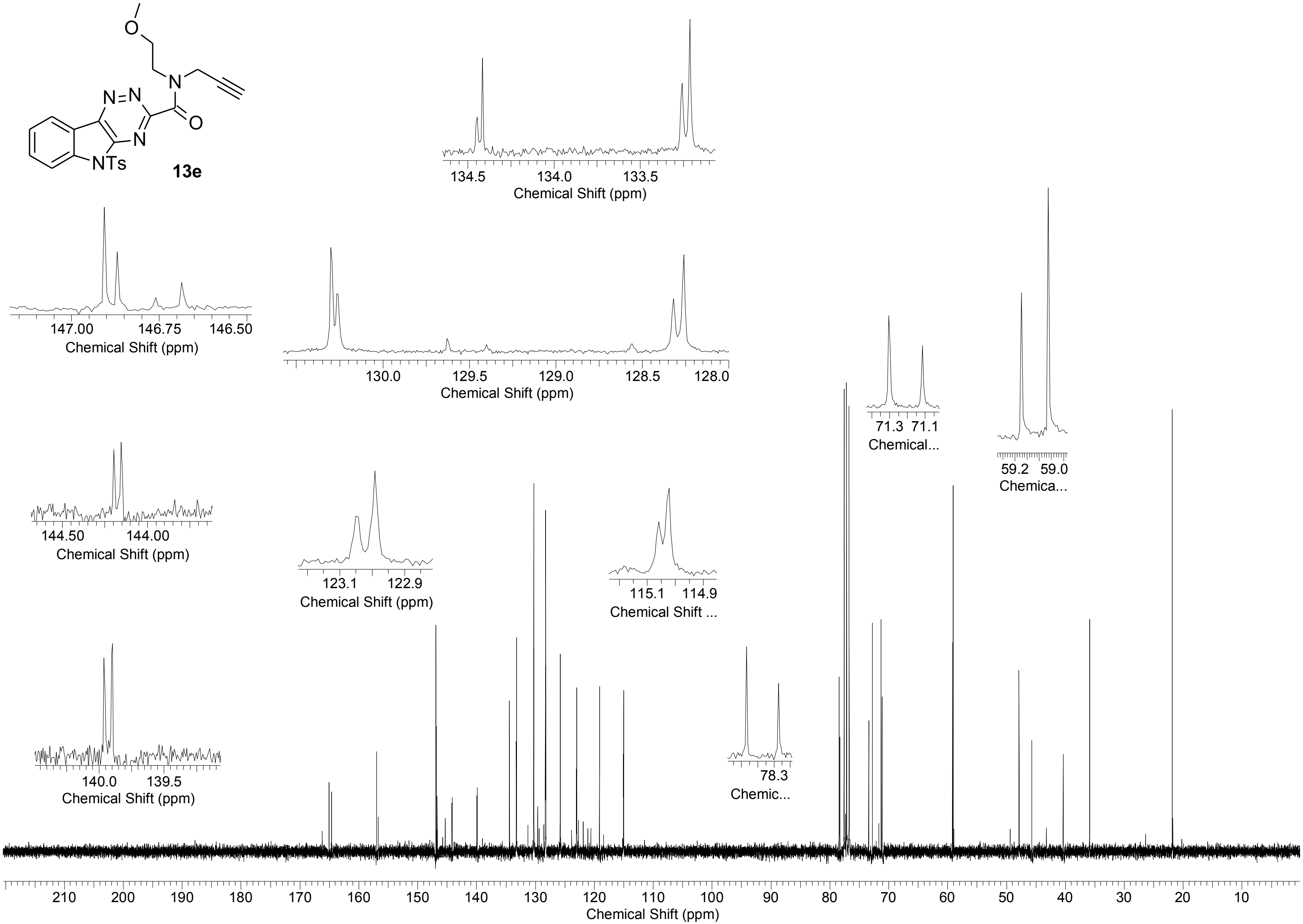
13e
134.5 134.0 133.5
Chemical Shift (ppm)
147.00 146.75 146.50
Chemical Shift (ppm)
130.0 129.5 129.0 128.5 128.0
Chemical Shift (ppm)
71.3 71.1
Chemical...
59.2 59.0
Chemica...
144.50 144.00
Chemical Shift (ppm)
123.1 122.9
Chemical Shift (ppm)
115.1 114.9
Chemical Shift ...
78.3
Chemic...
140.0 139.5
Chemical Shift (ppm)
210 200 190 180 170 160 150 140 130 120 110 100 90 80 70 60 50 40 30 20 10
Chemical Shift (ppm)

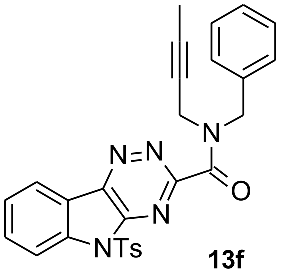
N=N
N
NTs
O
N

13f

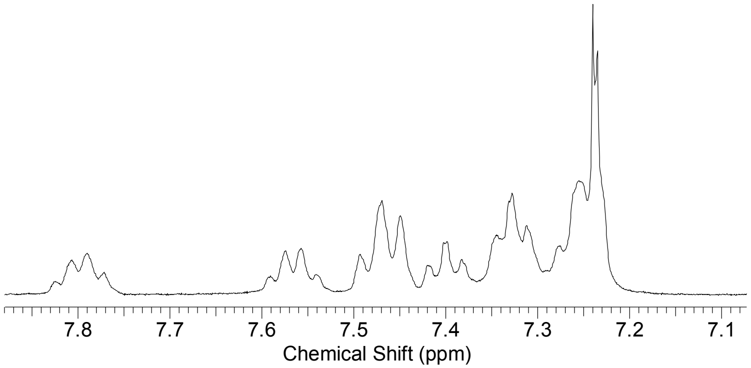
7.8
7.7
7.6
7.5
7.4
7.3
7.2
7.1
Chemical Shift (ppm)

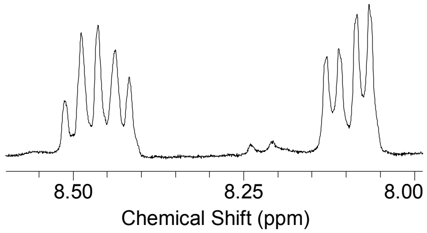
8.50
8.25
8.00
Chemical Shift (ppm)

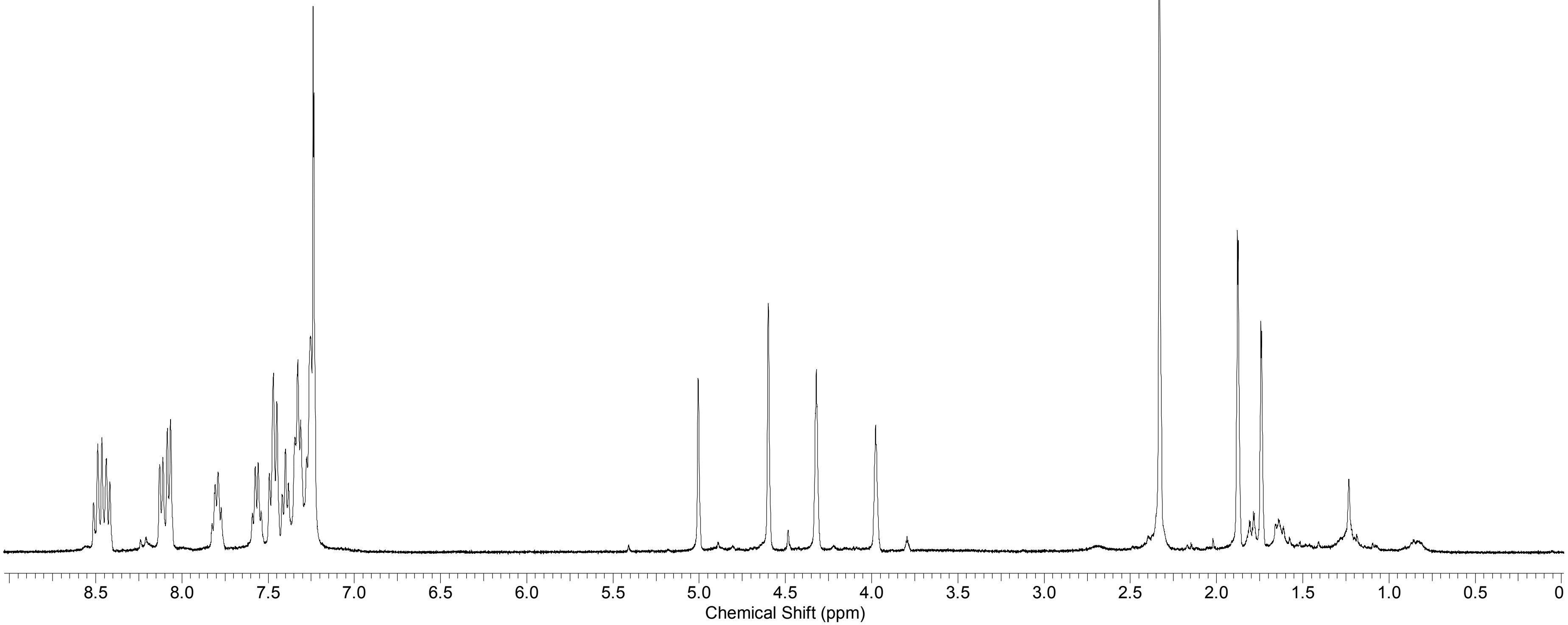
8.5
8.0
7.5
7.0
6.5
6.0
5.5
5.0
4.5
4.0
3.5
3.0
2.5
2.0
1.5
1.0
0.5
0
Chemical Shift (ppm)

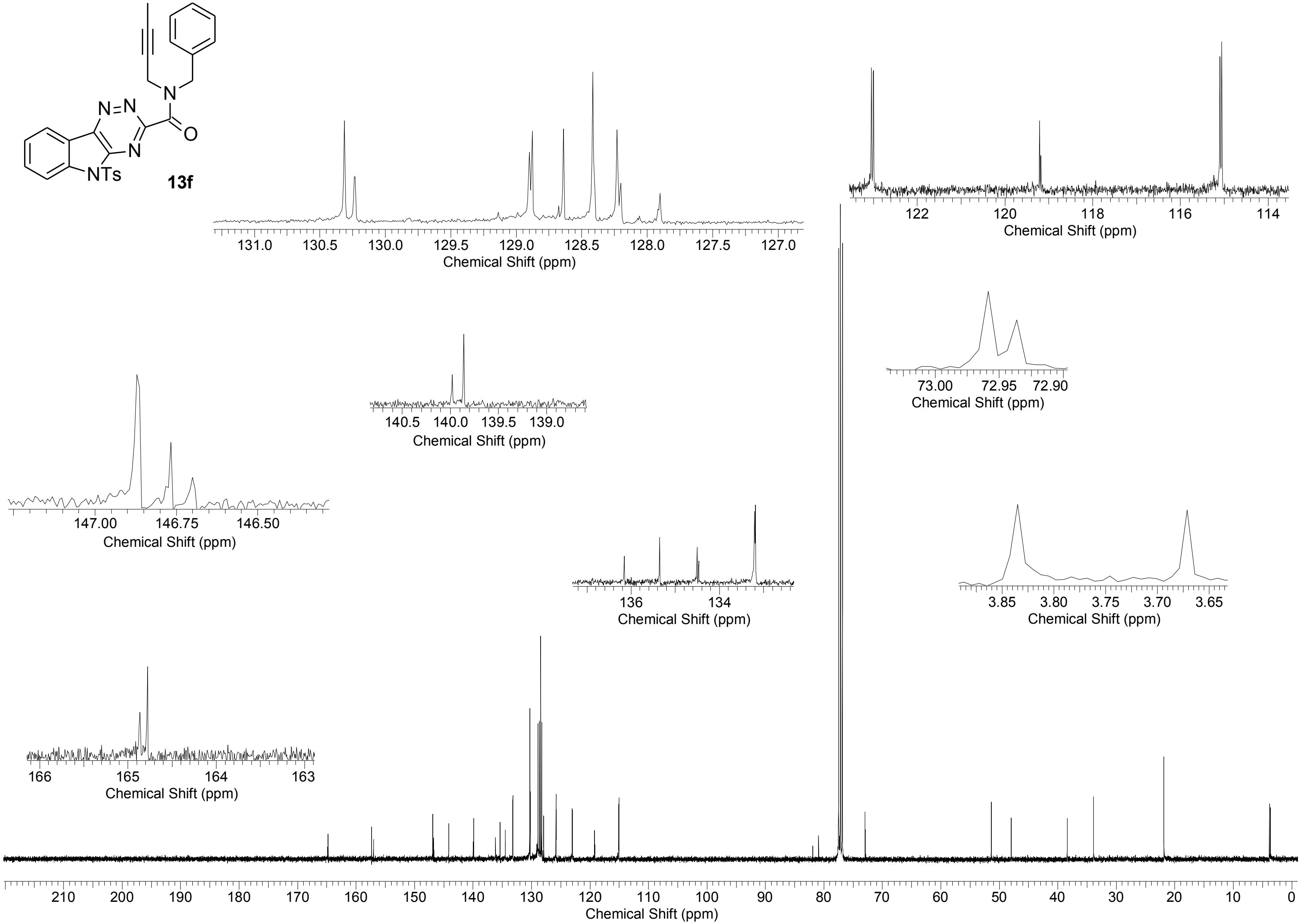

13f

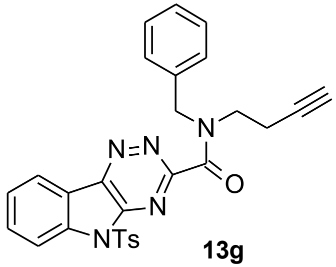

13g

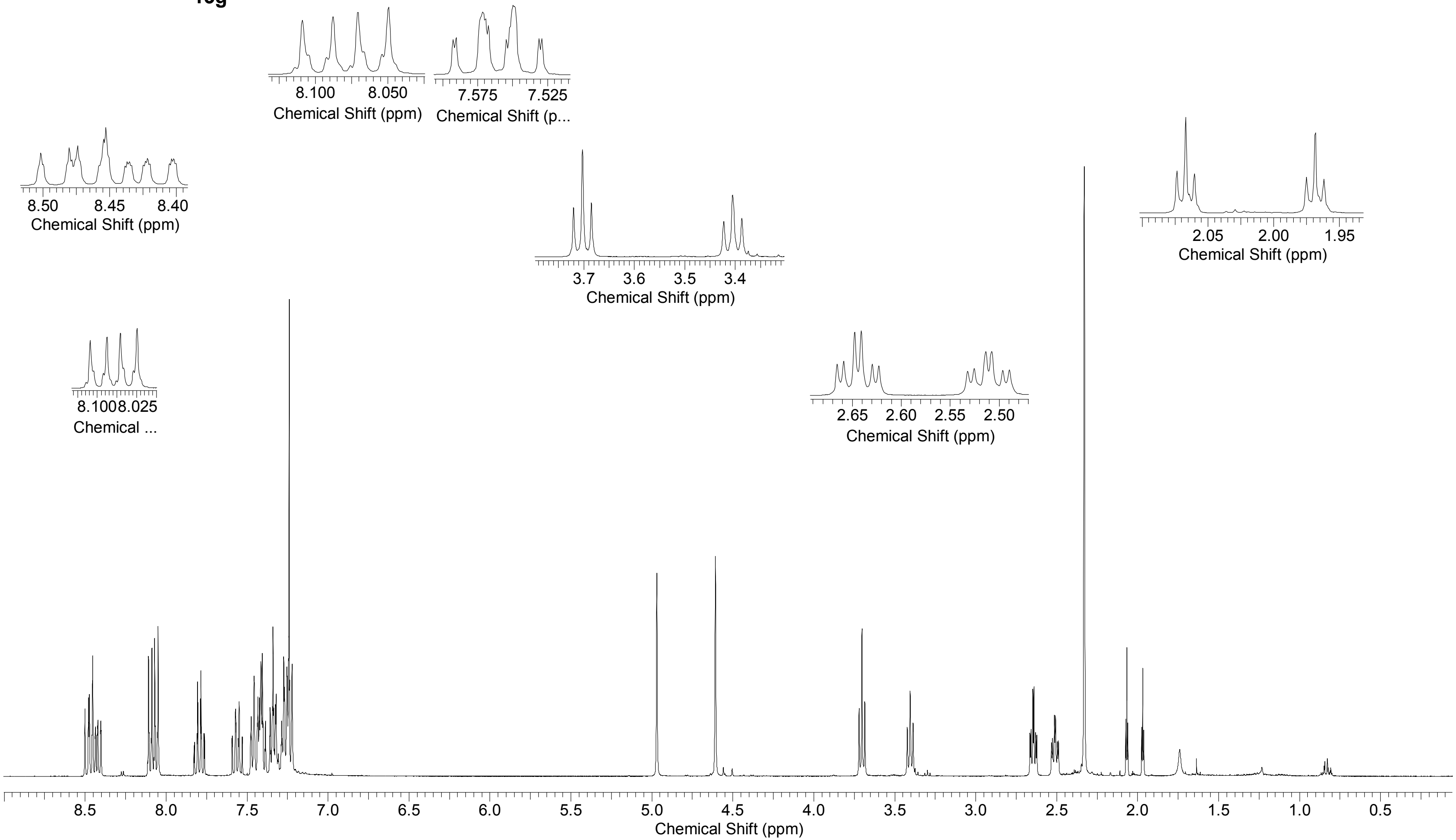

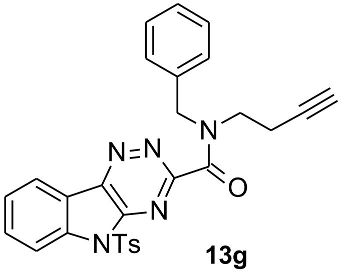
N=N
N
O
N
NTs

13g

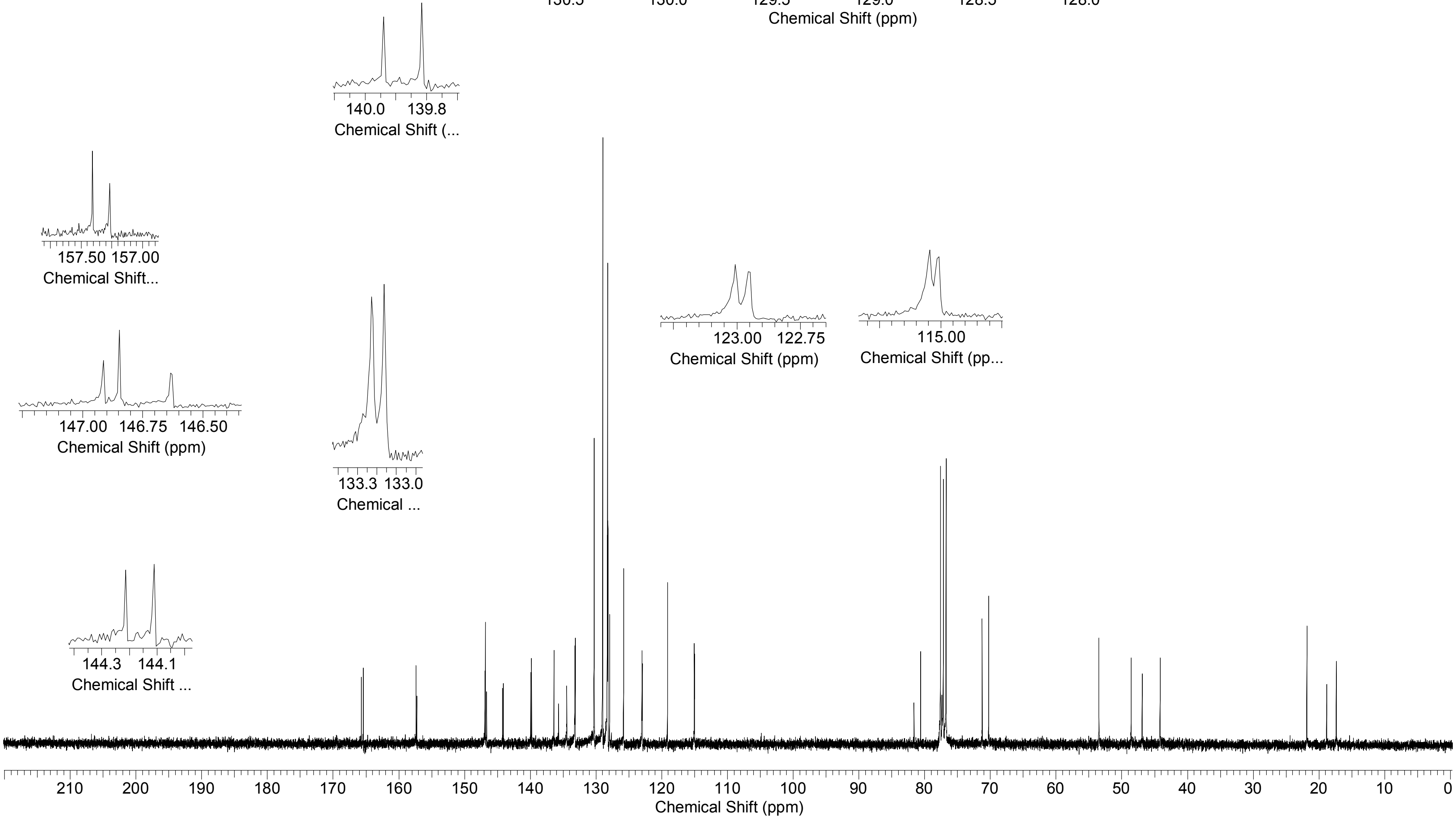
130.5 130.0 129.5 129.0 128.5 128.0
Chemical Shift (ppm)
140.0 139.8
Chemical Shift (...
157.50 157.00
Chemical Shift...
147.00 146.75 146.50
Chemical Shift (ppm)
133.3 133.0
Chemical ...
123.00 122.75
Chemical Shift (ppm)
115.00
Chemical Shift (pp...
144.3 144.1
Chemical Shift ...
210 200 190 180 170 160 150 140 130 120 110 100 90 80 70 60 50 40 30 20 10 0
Chemical Shift (ppm)

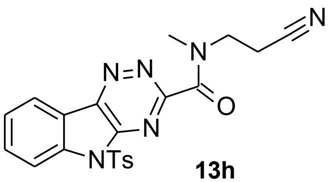
N
N=N
N
NTs
O

13h

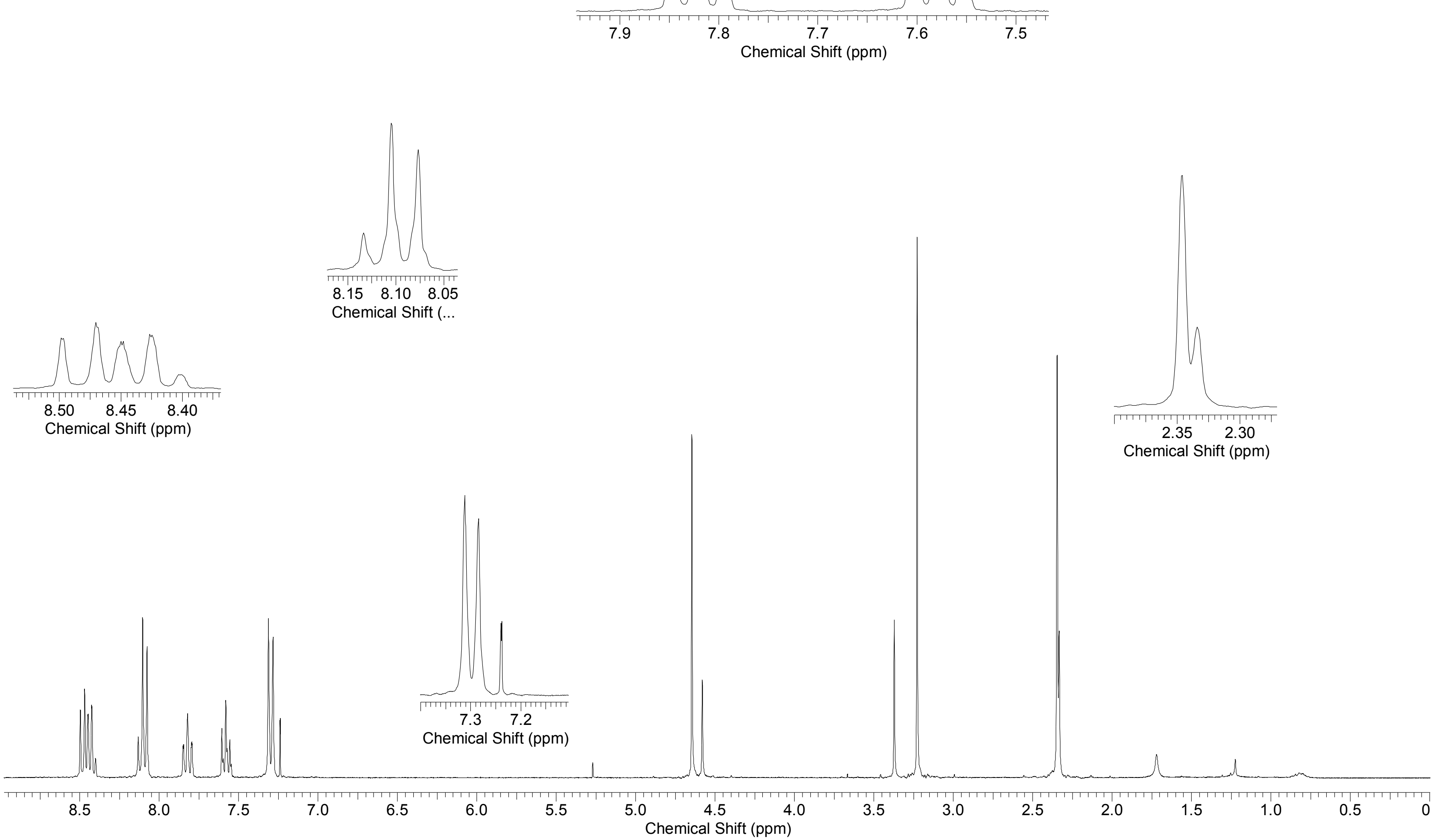
Chemical Shift (ppm)
Chemical Shift (...
Chemical Shift (ppm)
Chemical Shift (ppm)
Chemical Shift (ppm)
Chemical Shift (ppm)

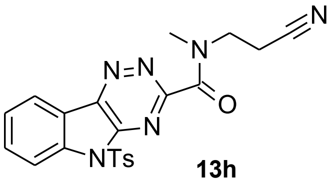
N=N
N
NTs
O
N
N

13h

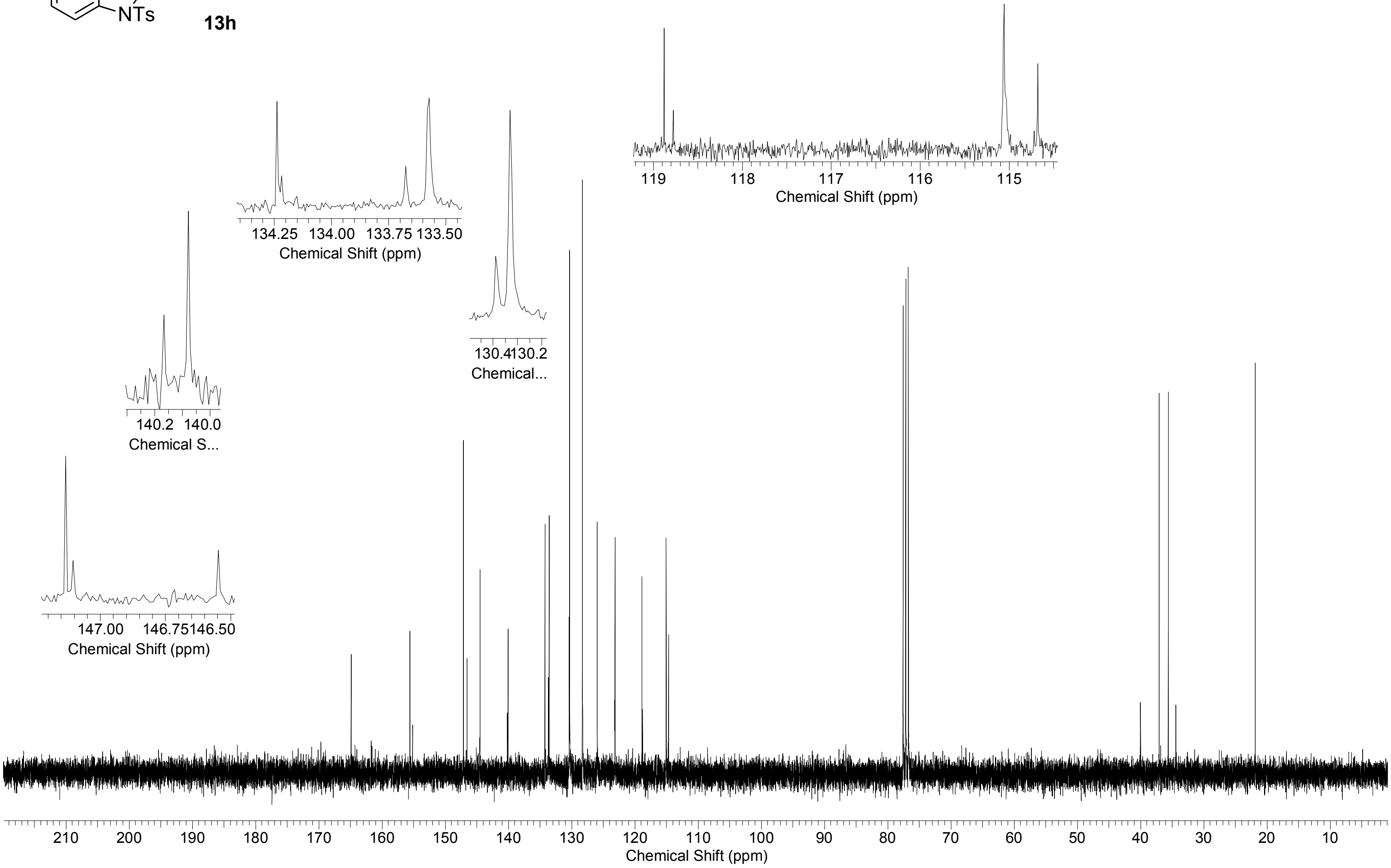
147.00 146.75 146.50
Chemical Shift (ppm)
140.2 140.0
Chemical S...
134.25 134.00 133.75 133.50
Chemical Shift (ppm)
130.4 130.2
Chemical...
119 118 117 116 115
Chemical Shift (ppm)
210 200 190 180 170 160 150 140 130 120 110 100 90 80 70 60 50 40 30 20 10
Chemical Shift (ppm)

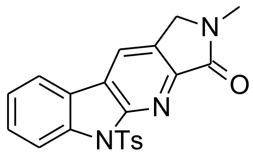

14a

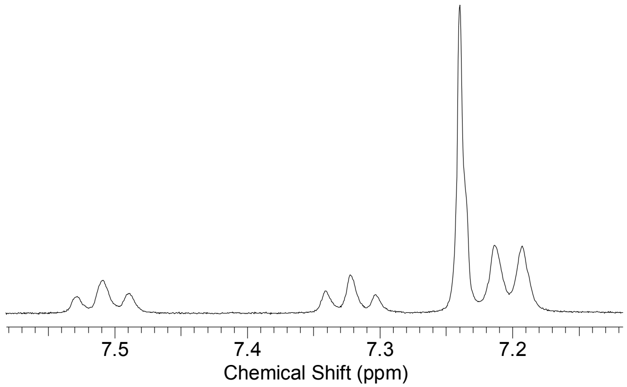
7.5
7.4
7.3
7.2
Chemical Shift (ppm)

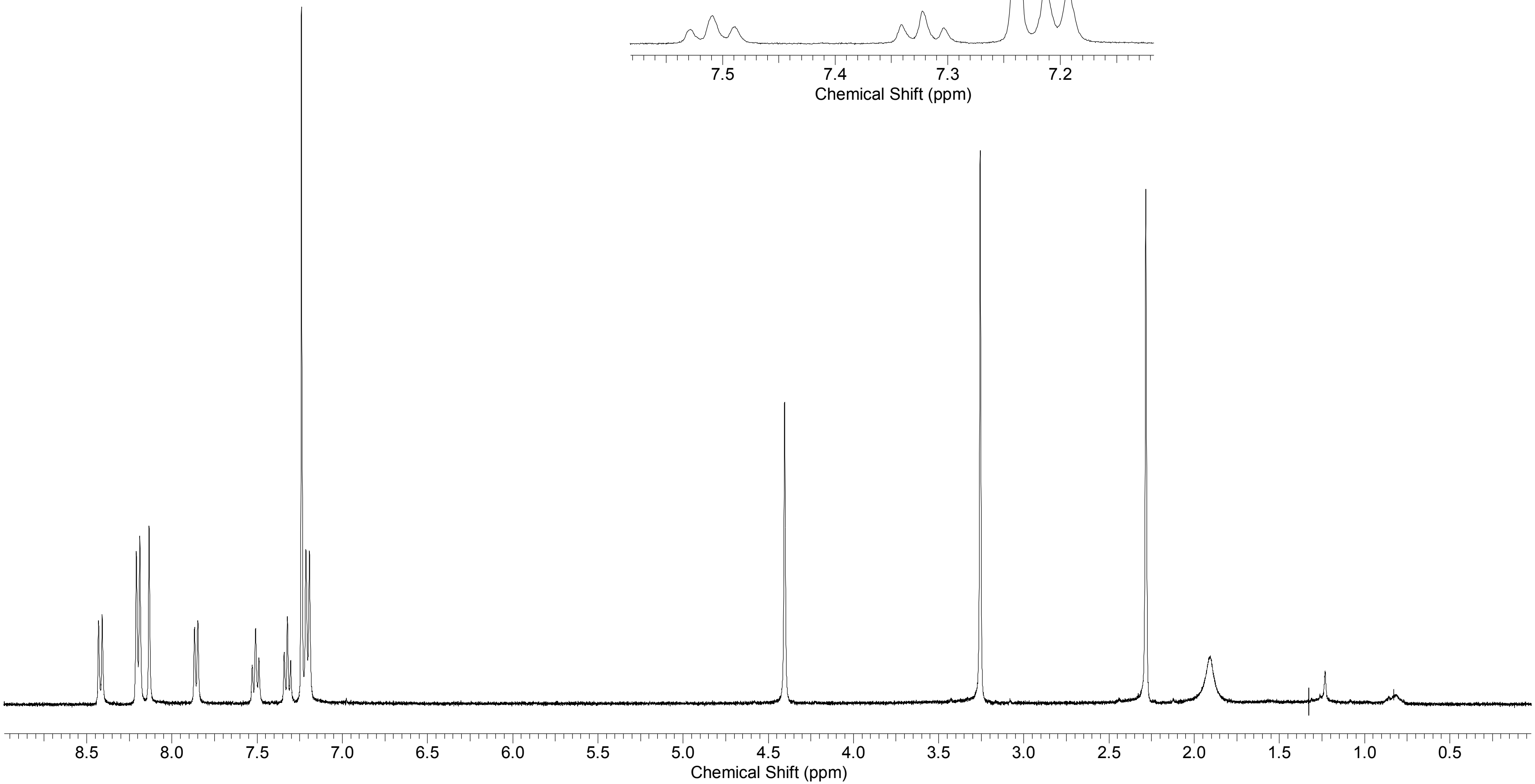
8.5
8.0
7.5
7.0
6.5
6.0
5.5
5.0
4.5
4.0
3.5
3.0
2.5
2.0
1.5
1.0
0.5
Chemical Shift (ppm)

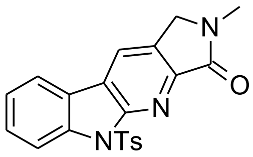

14a

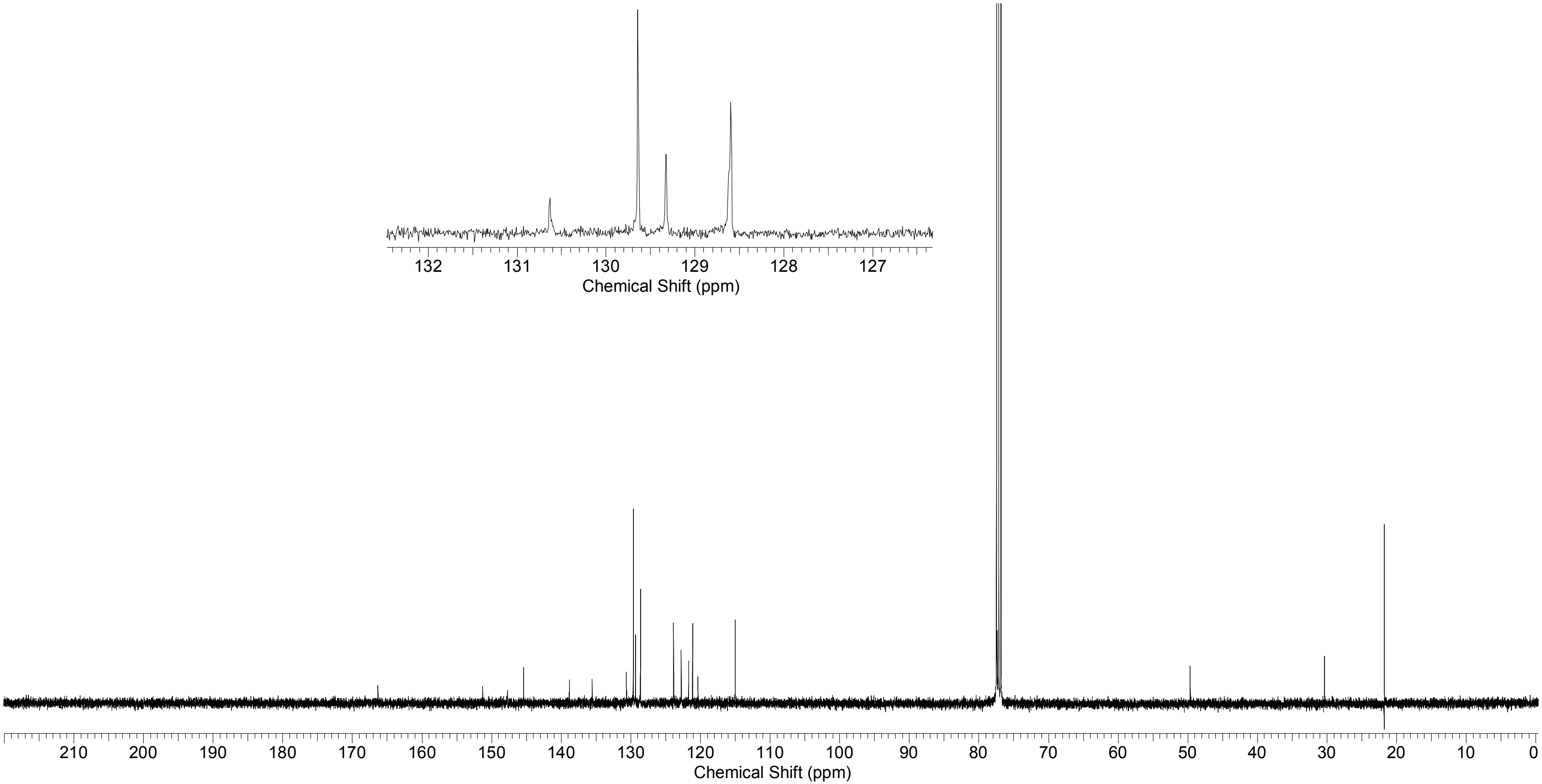

132
131
130
129
128
127
Chemical Shift (ppm)
210
200
190
180
170
160
150
140
130
120
110
100
90
80
70
60
50
40
30
20
10
0
Chemical Shift (ppm)

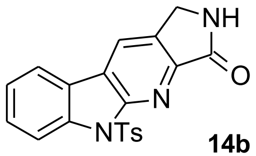

14b

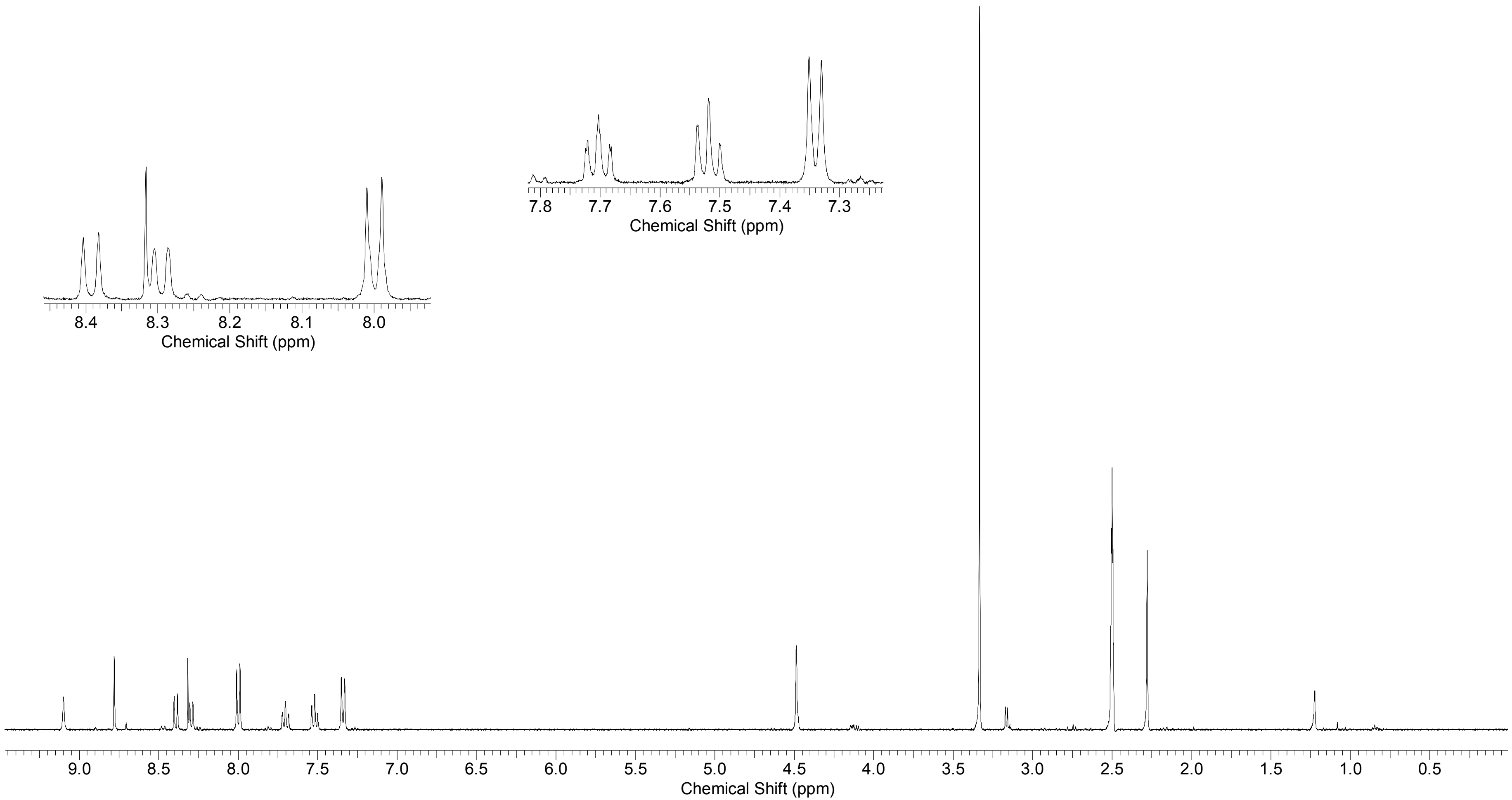

8.4
8.3
8.2
8.1
8.0
Chemical Shift (ppm)
7.8
7.7
7.6
7.5
7.4
7.3
Chemical Shift (ppm)
9.0
8.5
8.0
7.5
7.0
6.5
6.0
5.5
5.0
4.5
4.0
3.5
3.0
2.5
2.0
1.5
1.0
0.5
Chemical Shift (ppm)

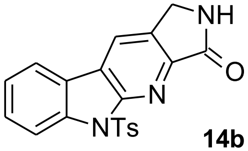
NH
O
N
NTs

14b

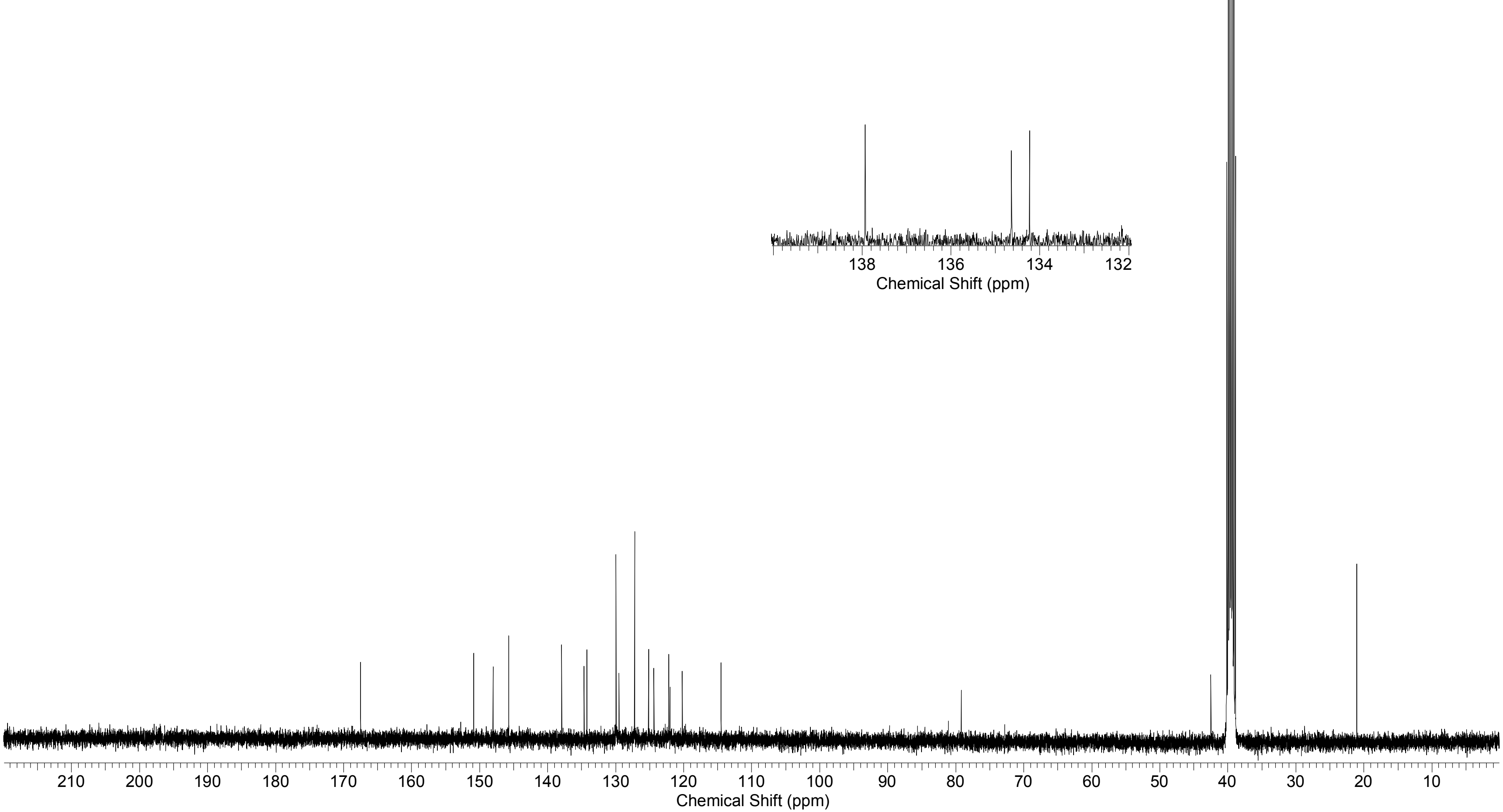
138
136
134
132
Chemical Shift (ppm)
210
200
190
180
170
160
150
140
130
120
110
100
90
80
70
60
50
40
30
20
10
Chemical Shift (ppm)

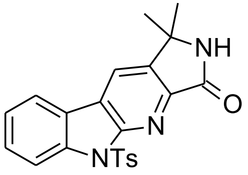
NH
O
N
NTs

14c

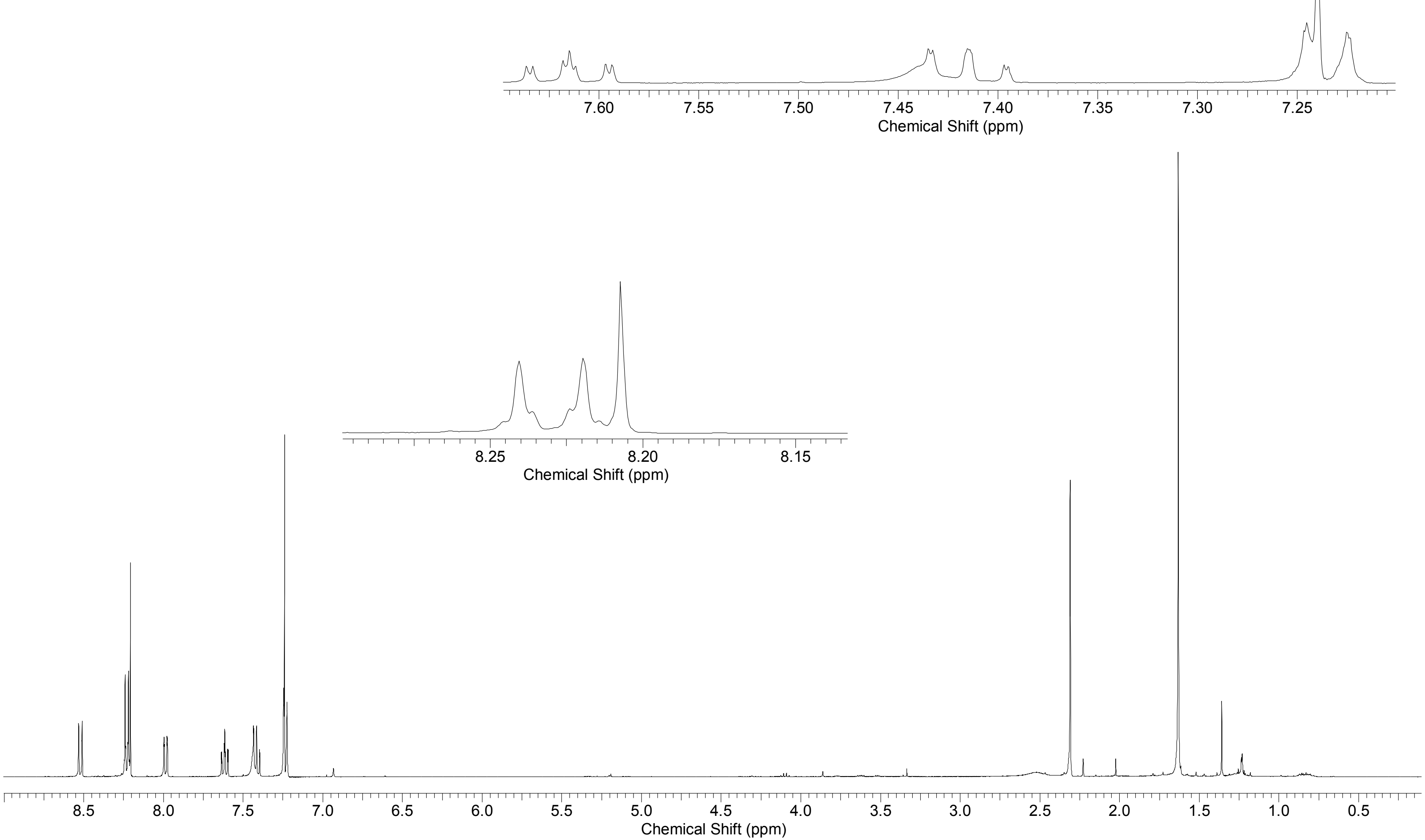
7.60 7.55 7.50 7.45 7.40 7.35 7.30 7.25
Chemical Shift (ppm)
8.25 8.20 8.15
Chemical Shift (ppm)
8.5 8.0 7.5 7.0 6.5 6.0 5.5 5.0 4.5 4.0 3.5 3.0 2.5 2.0 1.5 1.0 0.5
Chemical Shift (ppm)

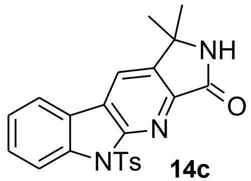
NH
O
N
NTs

14c

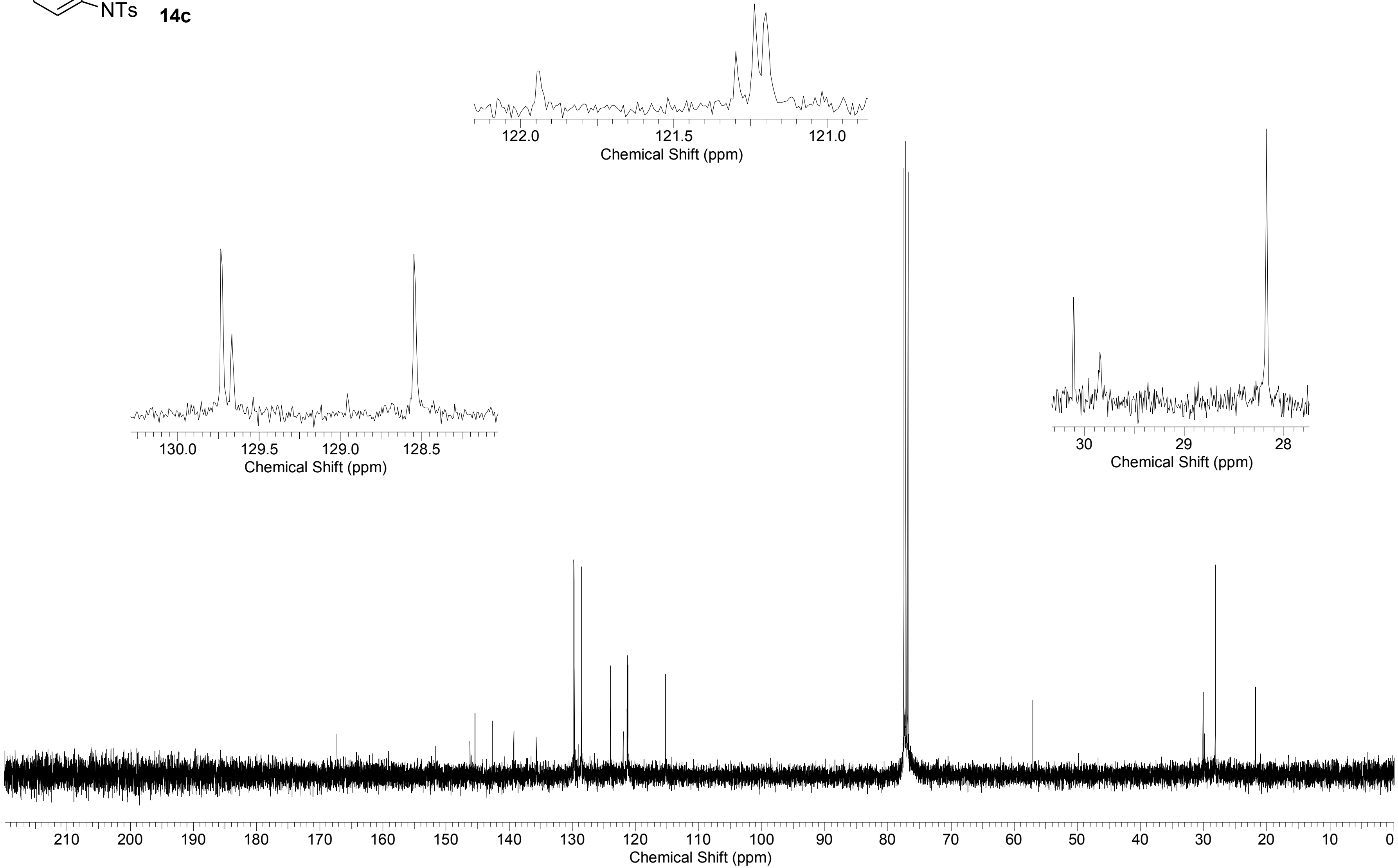
122.0
121.5
121.0
Chemical Shift (ppm)
130.0
129.5
129.0
128.5
Chemical Shift (ppm)
30
29
28
Chemical Shift (ppm)
210 200 190 180 170 160 150 140 130 120 110 100 90 80 70 60 50 40 30 20 10 0
Chemical Shift (ppm)

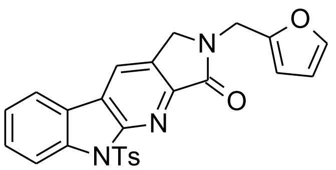
O
N
O
N
NTs

14d

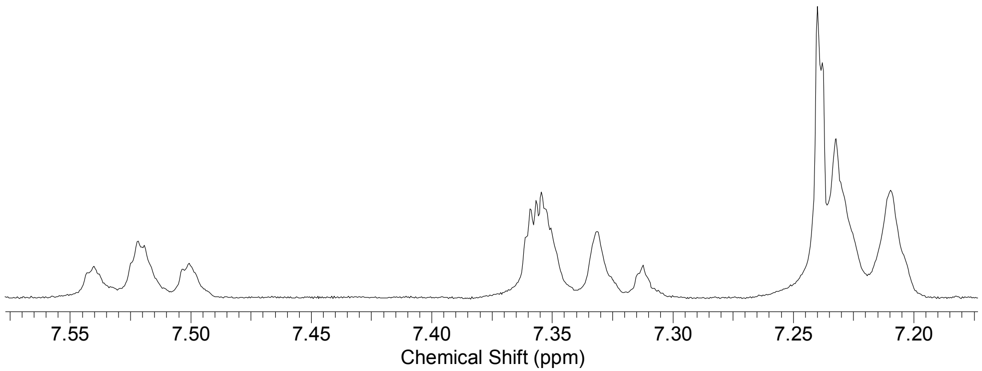
7.55
7.50
7.45
7.40
7.35
7.30
7.25
7.20
Chemical Shift (ppm)

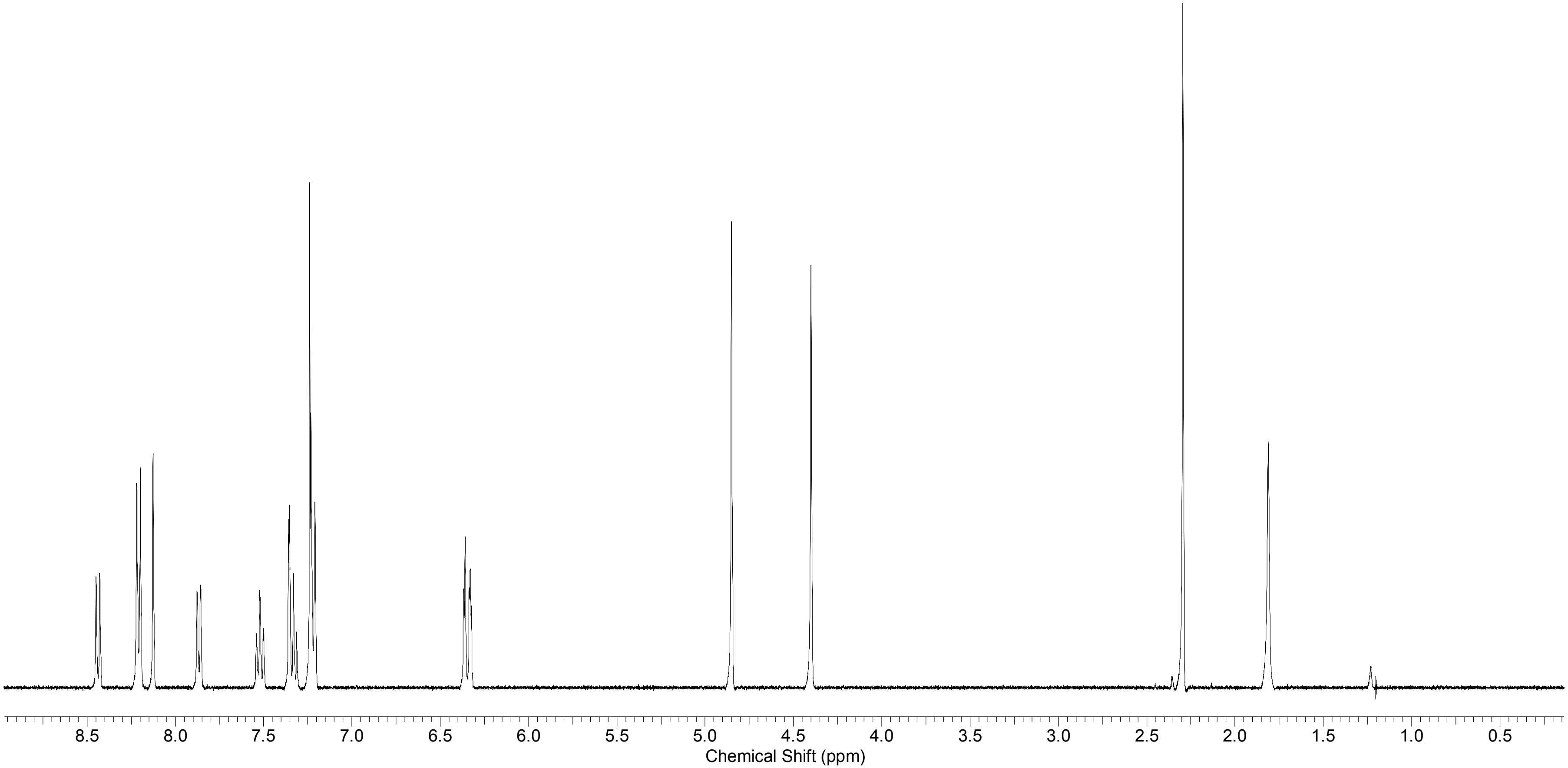
8.5
8.0
7.5
7.0
6.5
6.0
5.5
5.0
4.5
4.0
3.5
3.0
2.5
2.0
1.5
1.0
0.5
Chemical Shift (ppm)

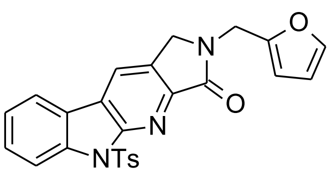
O
N
O
N
NTs

14d

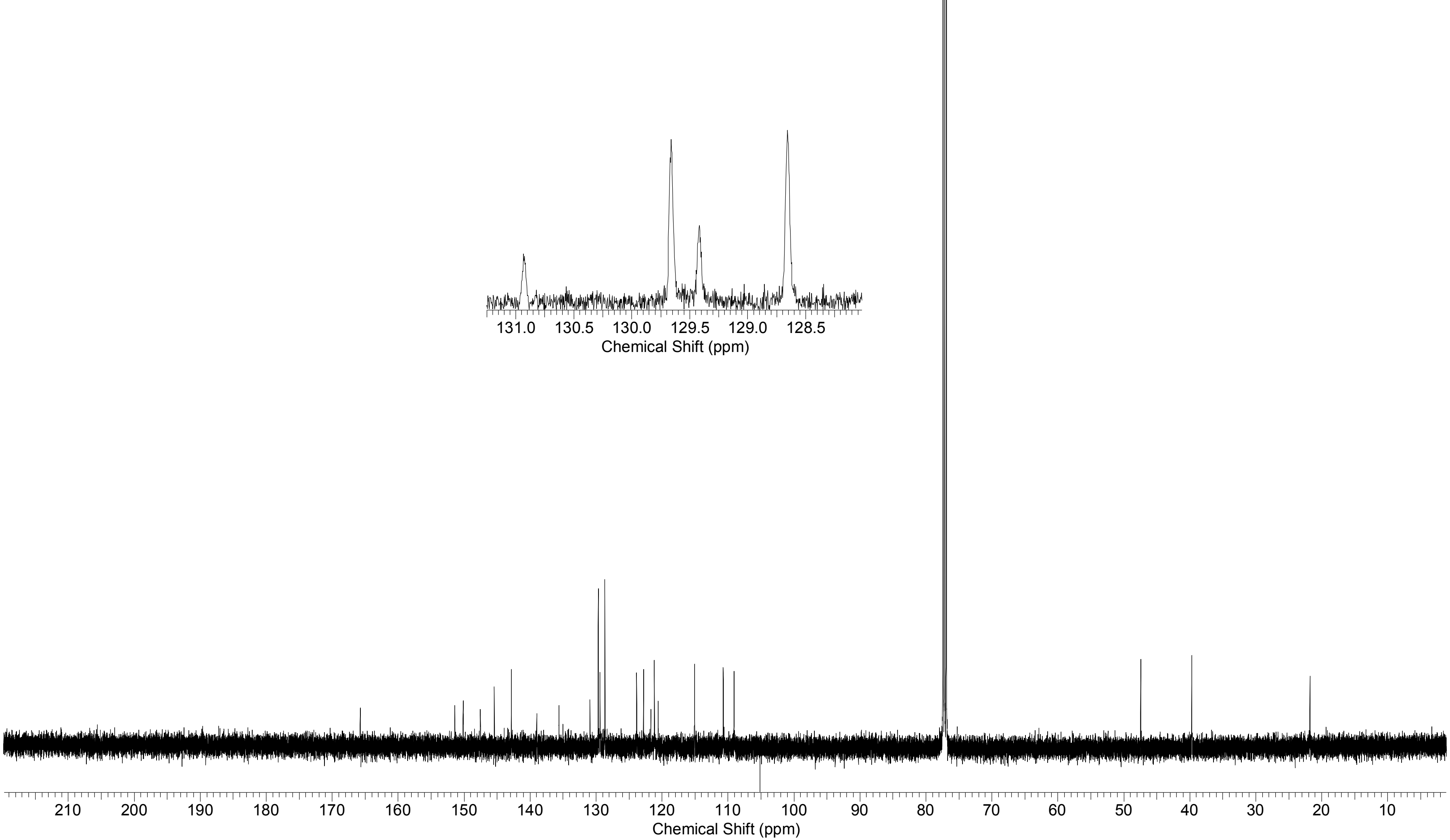
131.0 130.5 130.0 129.5 129.0 128.5
Chemical Shift (ppm)
210 200 190 180 170 160 150 140 130 120 110 100 90 80 70 60 50 40 30 20 10
Chemical Shift (ppm)

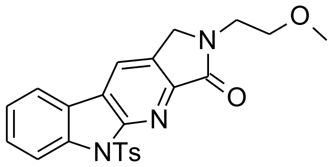

14e

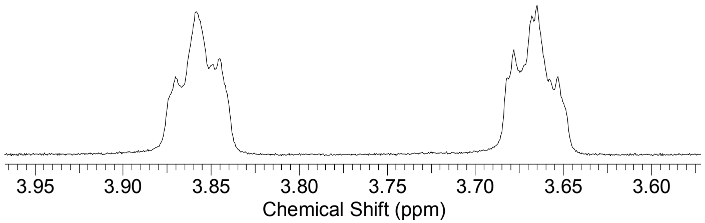

3.95 3.90 3.85 3.80 3.75 3.70 3.65 3.60
Chemical Shift (ppm)

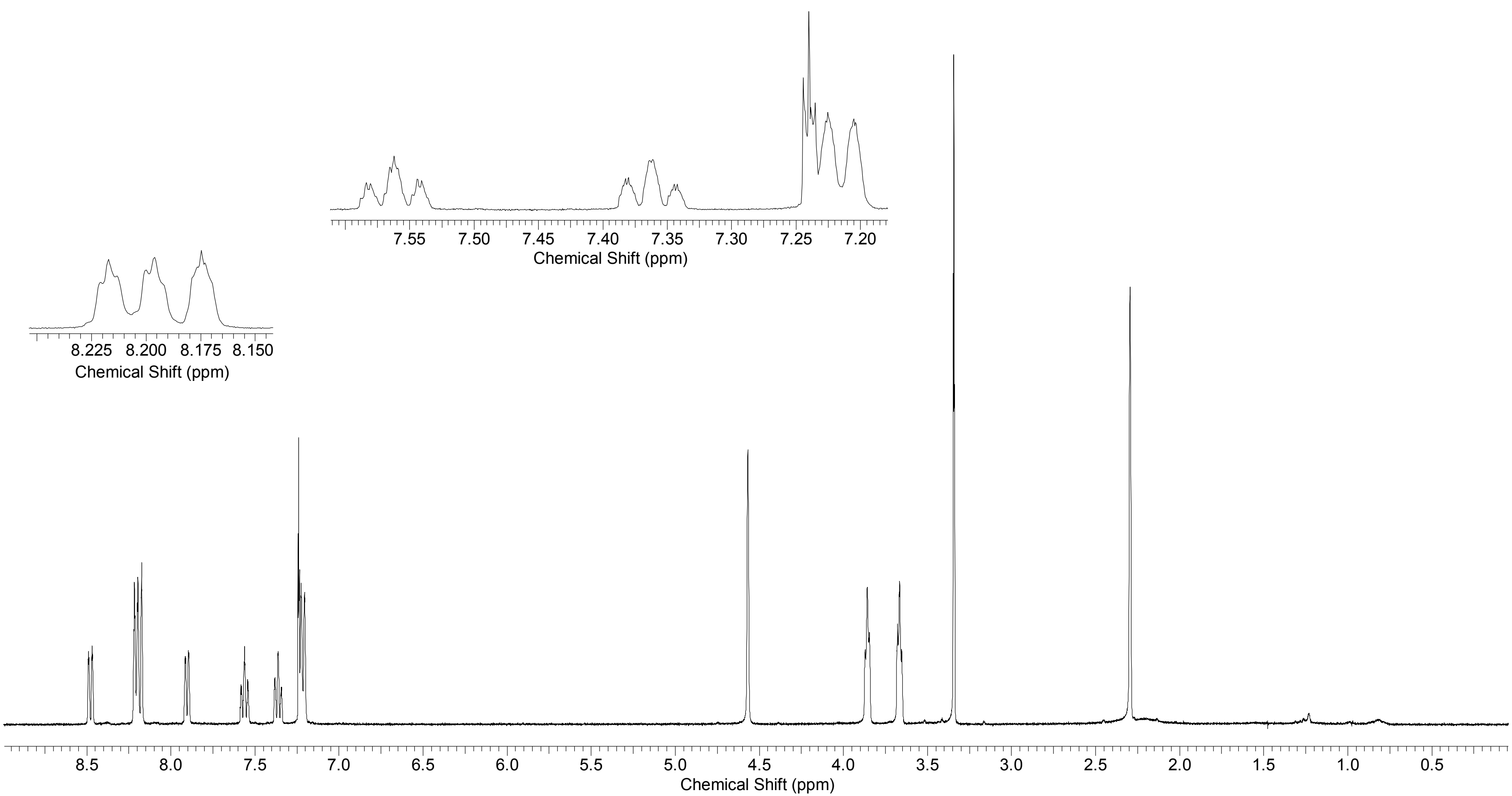

7.55 7.50 7.45 7.40 7.35 7.30 7.25 7.20
Chemical Shift (ppm)
8.225 8.200 8.175 8.150
Chemical Shift (ppm)
8.5 8.0 7.5 7.0 6.5 6.0 5.5 5.0 4.5 4.0 3.5 3.0 2.5 2.0 1.5 1.0 0.5
Chemical Shift (ppm)

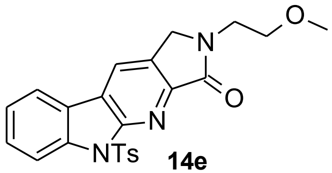
O
N
O
N
NTs

14e

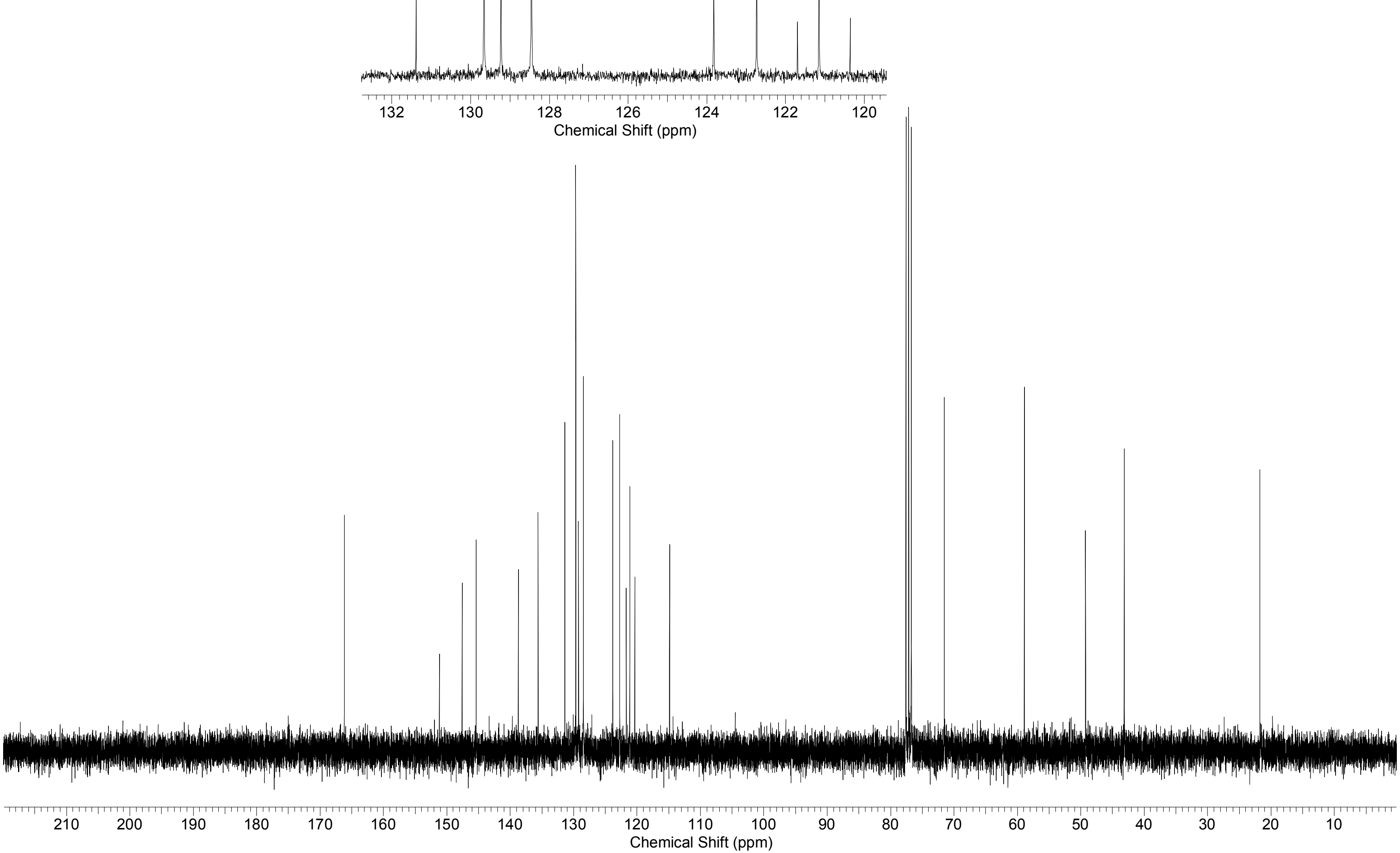
132 130 128 126 124 122 120
Chemical Shift (ppm)
210 200 190 180 170 160 150 140 130 120 110 100 90 80 70 60 50 40 30 20 10
Chemical Shift (ppm)

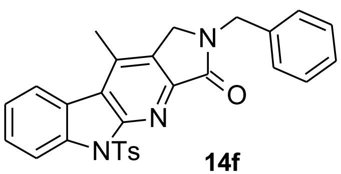
NTs
N
O
N

14f

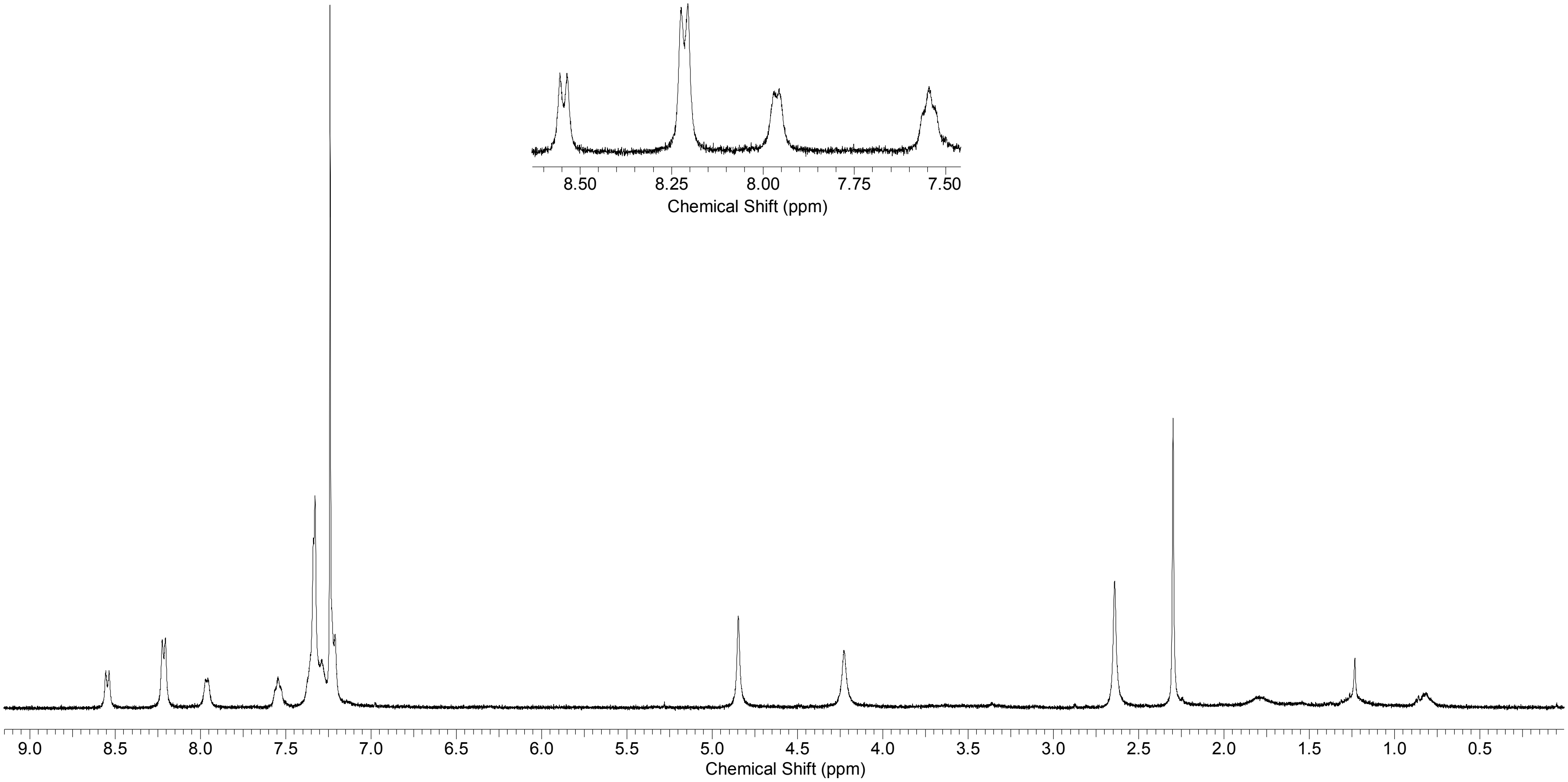
8.50
8.25
8.00
7.75
7.50
Chemical Shift (ppm)
9.0
8.5
8.0
7.5
7.0
6.5
6.0
5.5
5.0
4.5
4.0
3.5
3.0
2.5
2.0
1.5
1.0
0.5
Chemical Shift (ppm)

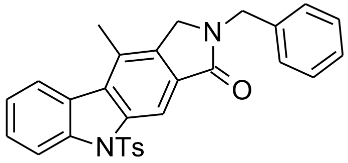
N
O
NTs

14f

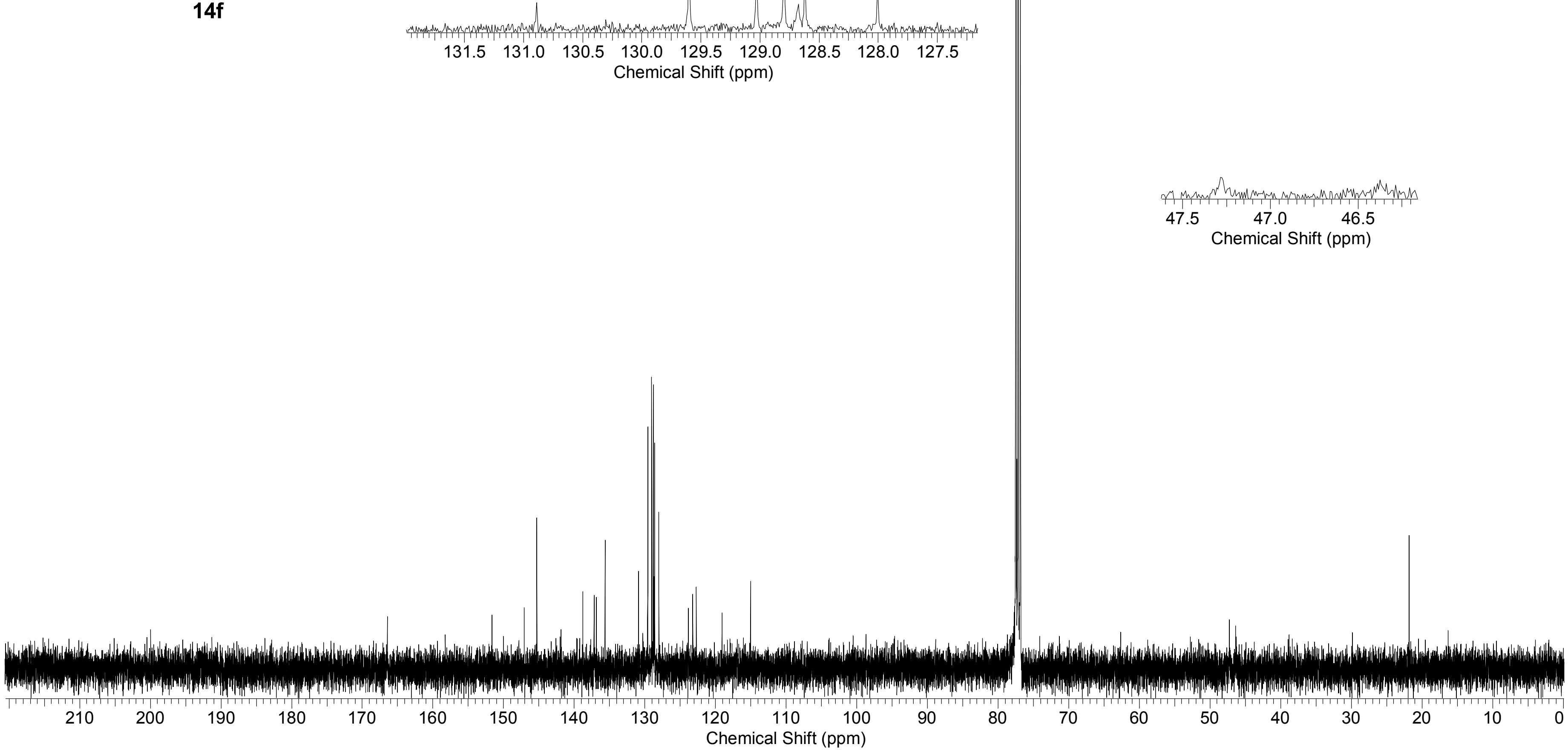
131.5 131.0 130.5 130.0 129.5 129.0 128.5 128.0 127.5
Chemical Shift (ppm)
47.5 47.0 46.5
Chemical Shift (ppm)
210 200 190 180 170 160 150 140 130 120 110 100 90 80 70 60 50 40 30 20 10 0
Chemical Shift (ppm)

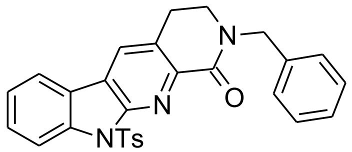

14g

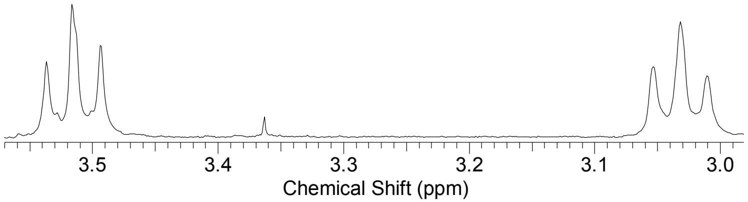

3.5
3.4
3.3
3.2
3.1
3.0
Chemical Shift (ppm)

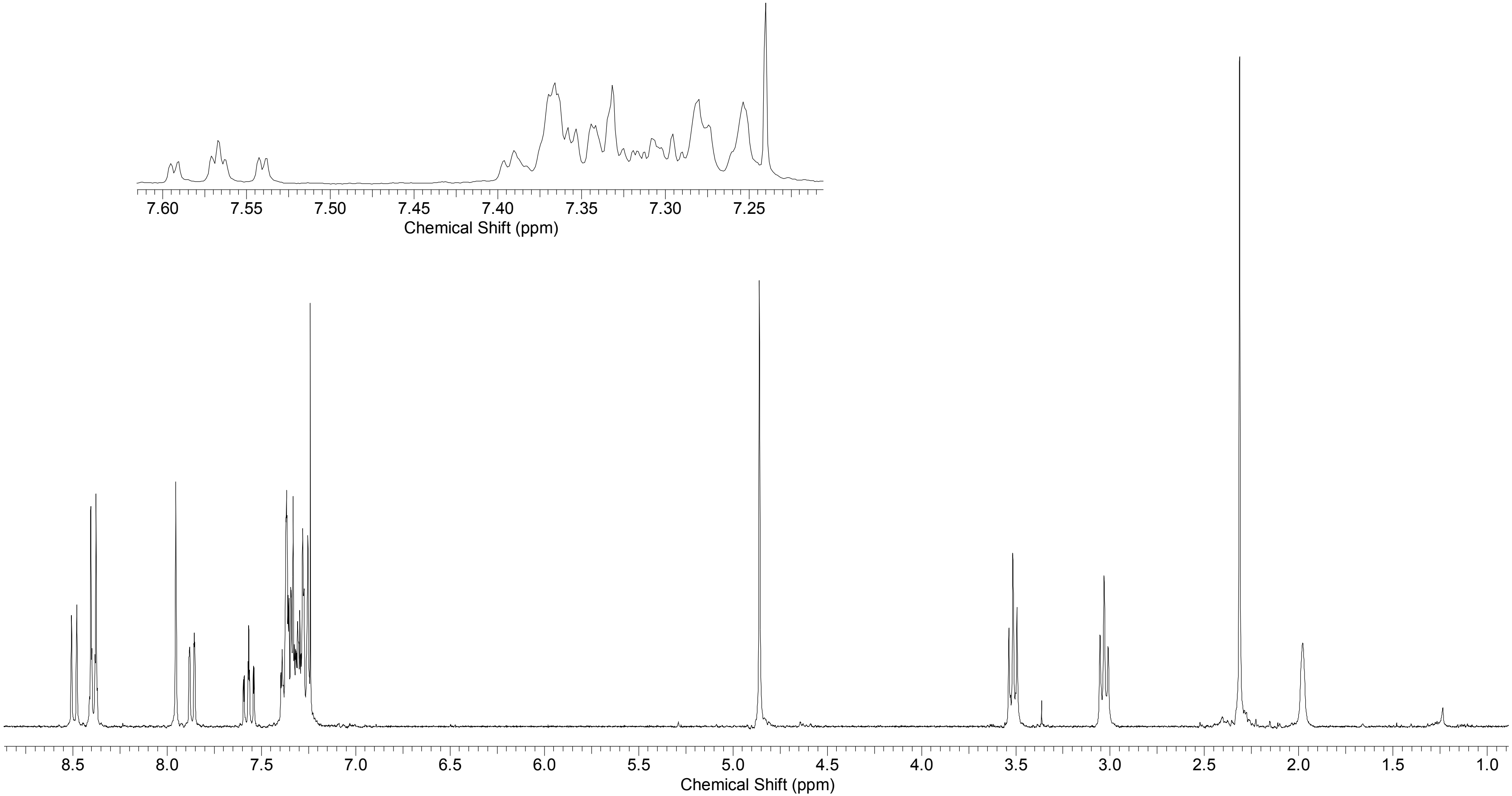

7.60
7.55
7.50
7.45
7.40
7.35
7.30
7.25
Chemical Shift (ppm)
8.5
8.0
7.5
7.0
6.5
6.0
5.5
5.0
4.5
4.0
3.5
3.0
2.5
2.0
1.5
1.0
Chemical Shift (ppm)

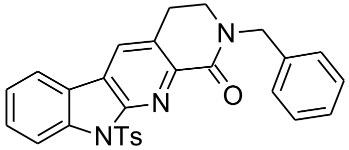
NTs
N
O
N

14g

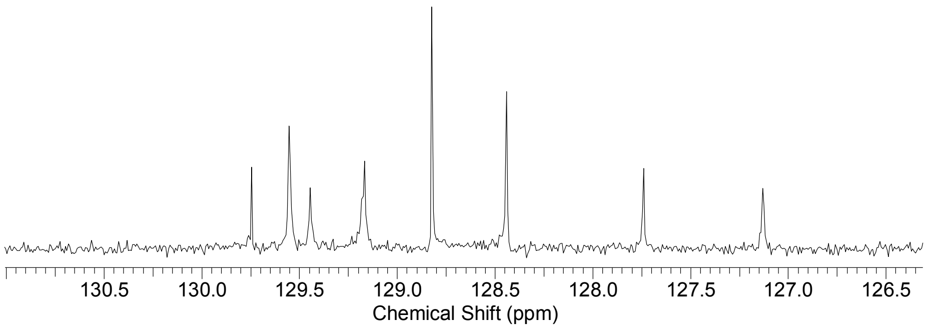
130.5 130.0 129.5 129.0 128.5 128.0 127.5 127.0 126.5
Chemical Shift (ppm)

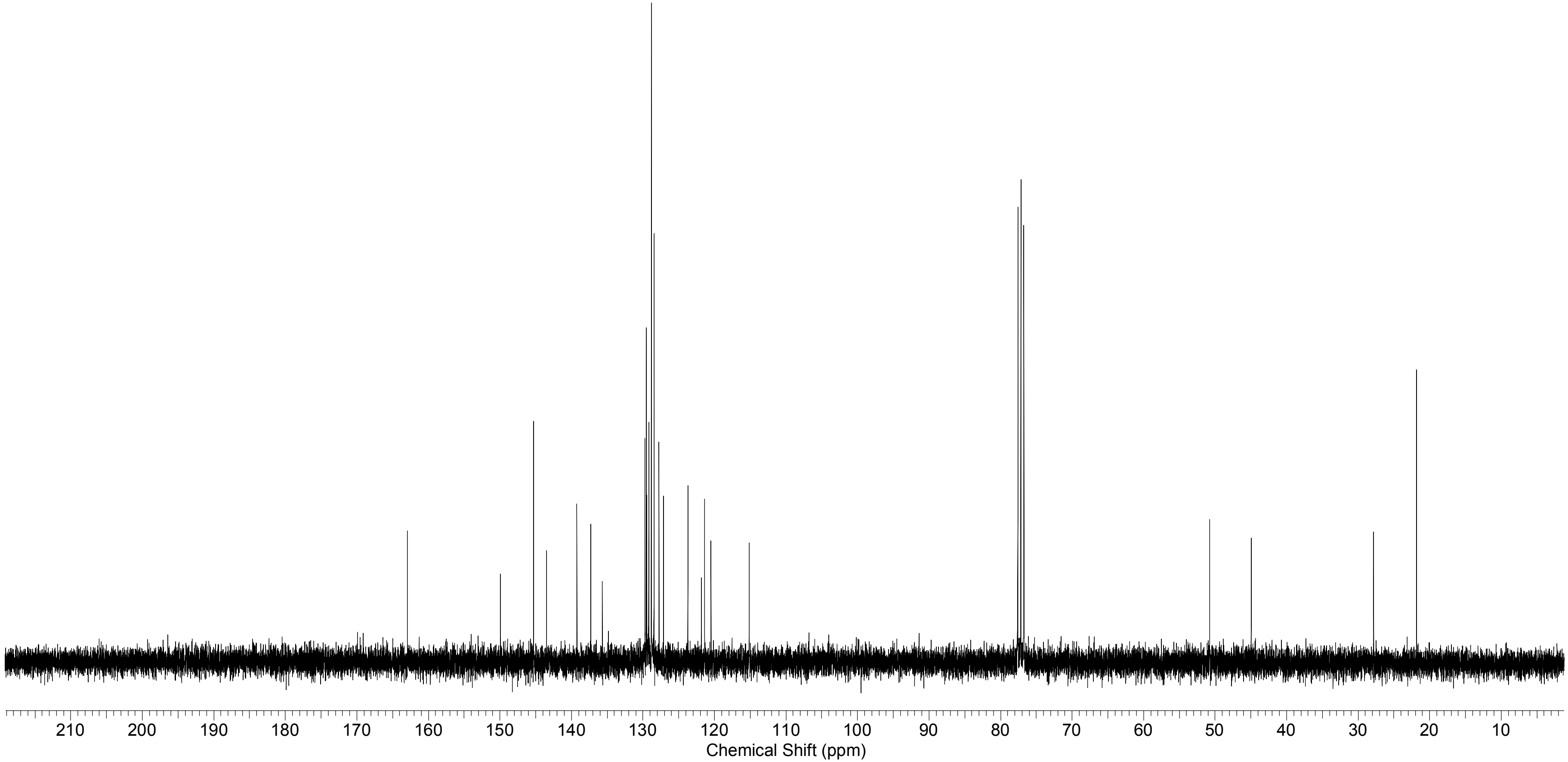
210 200 190 180 170 160 150 140 130 120 110 100 90 80 70 60 50 40 30 20 10
Chemical Shift (ppm)

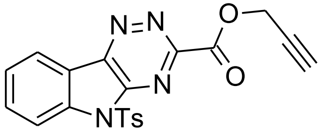

17a

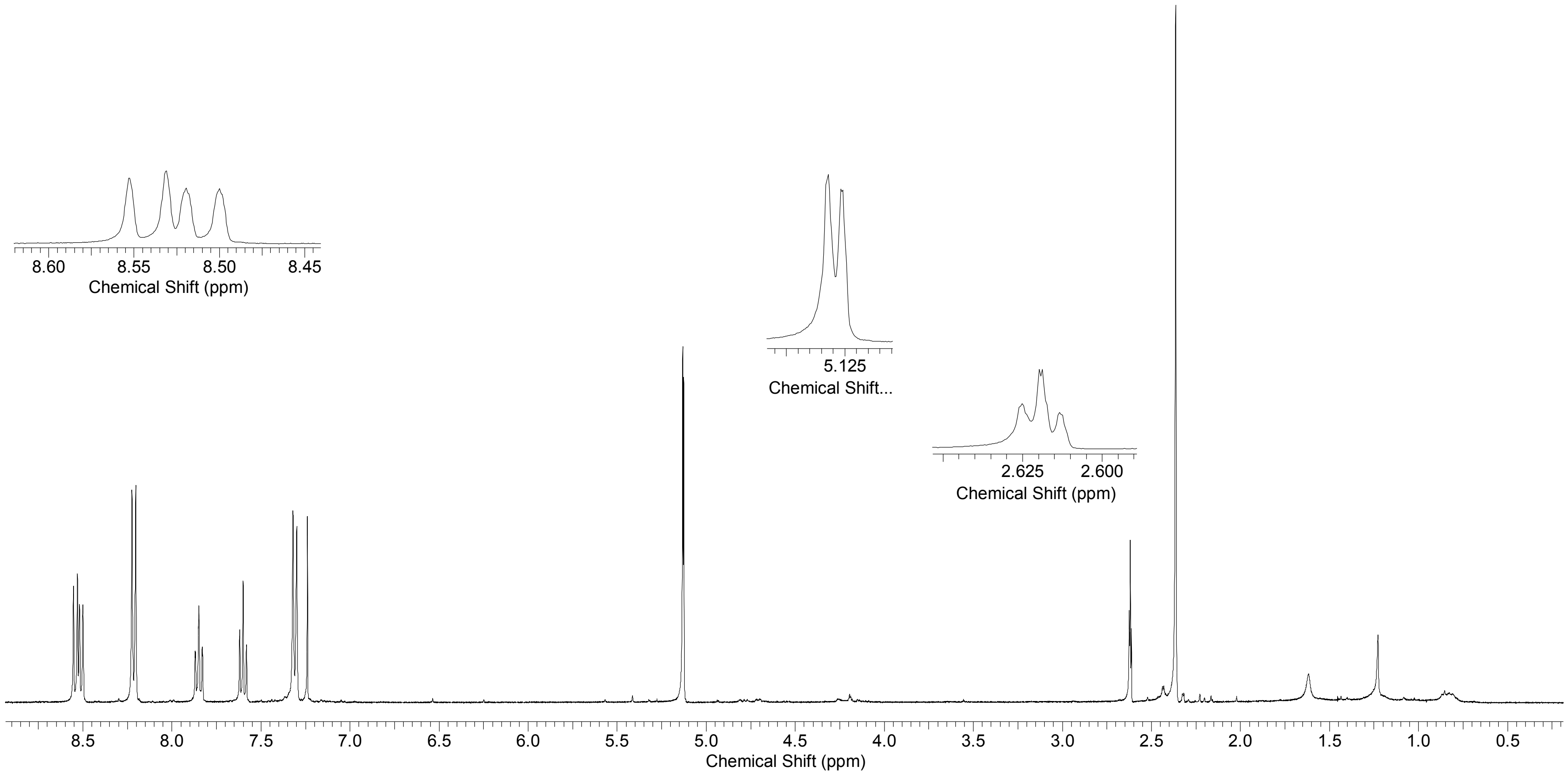

8.60 8.55 8.50 8.45
Chemical Shift (ppm)
5.125
Chemical Shift...
2.625 2.600
Chemical Shift (ppm)
8.5 8.0 7.5 7.0 6.5 6.0 5.5 5.0 4.5 4.0 3.5 3.0 2.5 2.0 1.5 1.0 0.5
Chemical Shift (ppm)

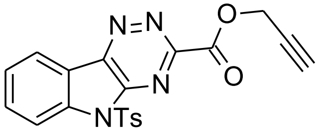
N=N
O
N
O
NTs

17a

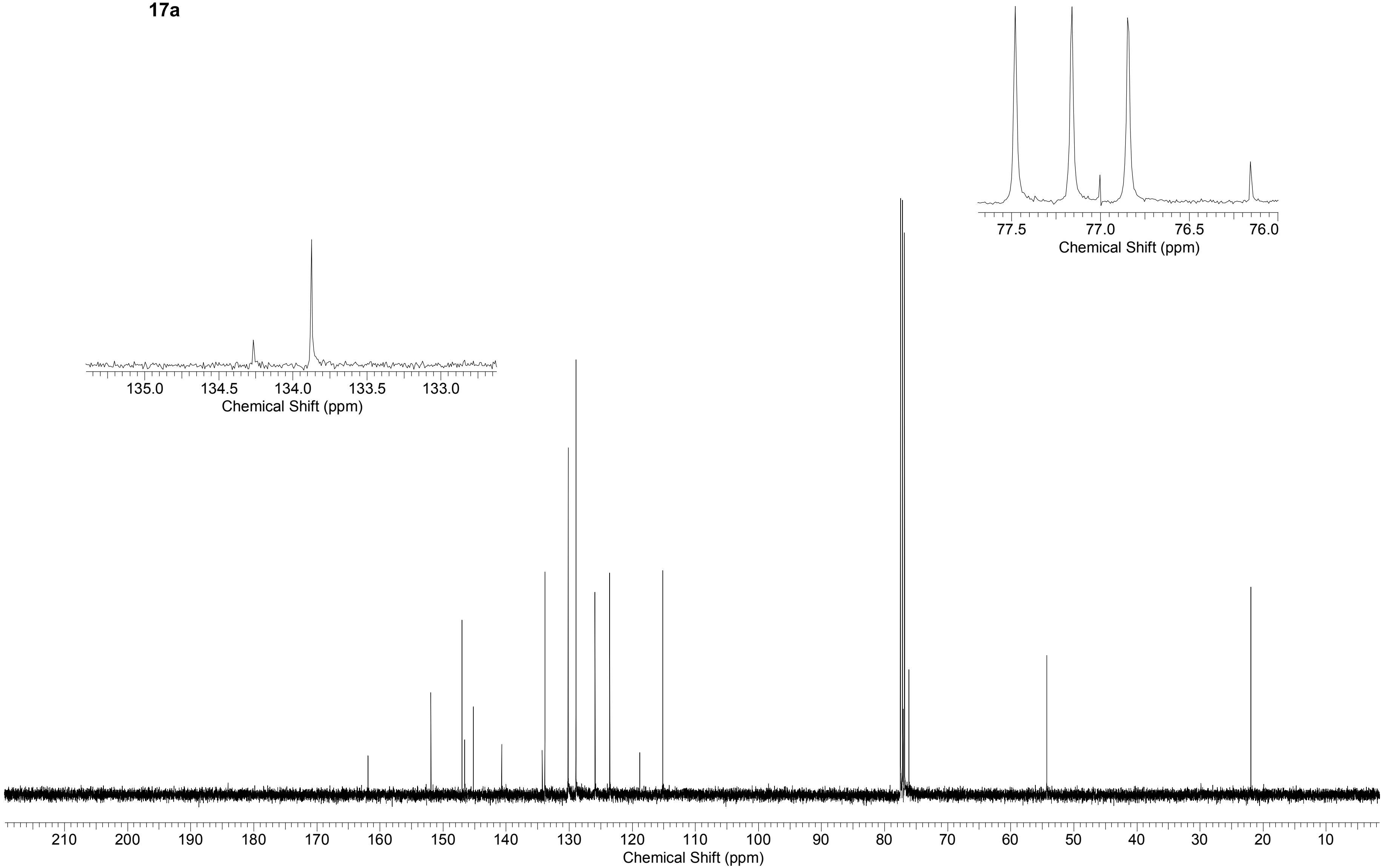
77.5
77.0
76.5
76.0
Chemical Shift (ppm)
135.0
134.5
134.0
133.5
133.0
Chemical Shift (ppm)
210
200
190
180
170
160
150
140
130
120
110
100
90
80
70
60
50
40
30
20
10
Chemical Shift (ppm)

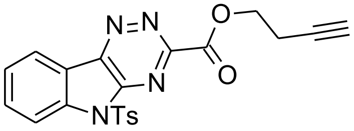

17b

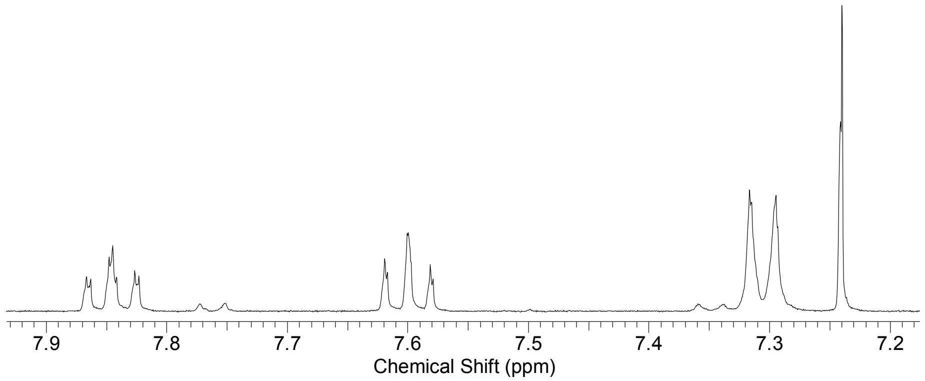
7.9 7.8 7.7 7.6 7.5 7.4 7.3 7.2
Chemical Shift (ppm)

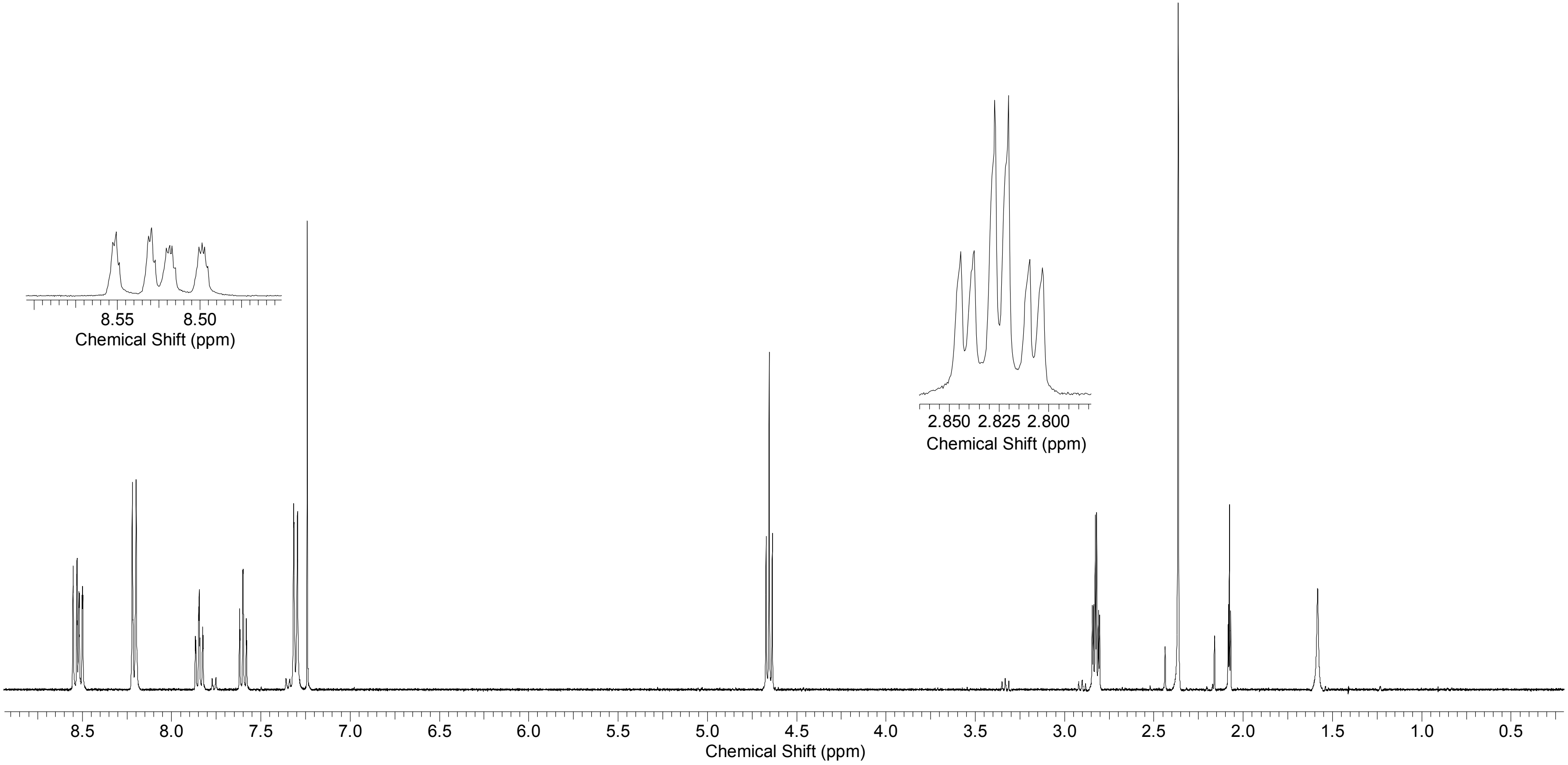
8.55 8.50
Chemical Shift (ppm)
2.850 2.825 2.800
Chemical Shift (ppm)
8.5 8.0 7.5 7.0 6.5 6.0 5.5 5.0 4.5 4.0 3.5 3.0 2.5 2.0 1.5 1.0 0.5
Chemical Shift (ppm)

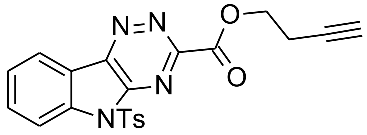
N=N
O
N
NTs
O

17b

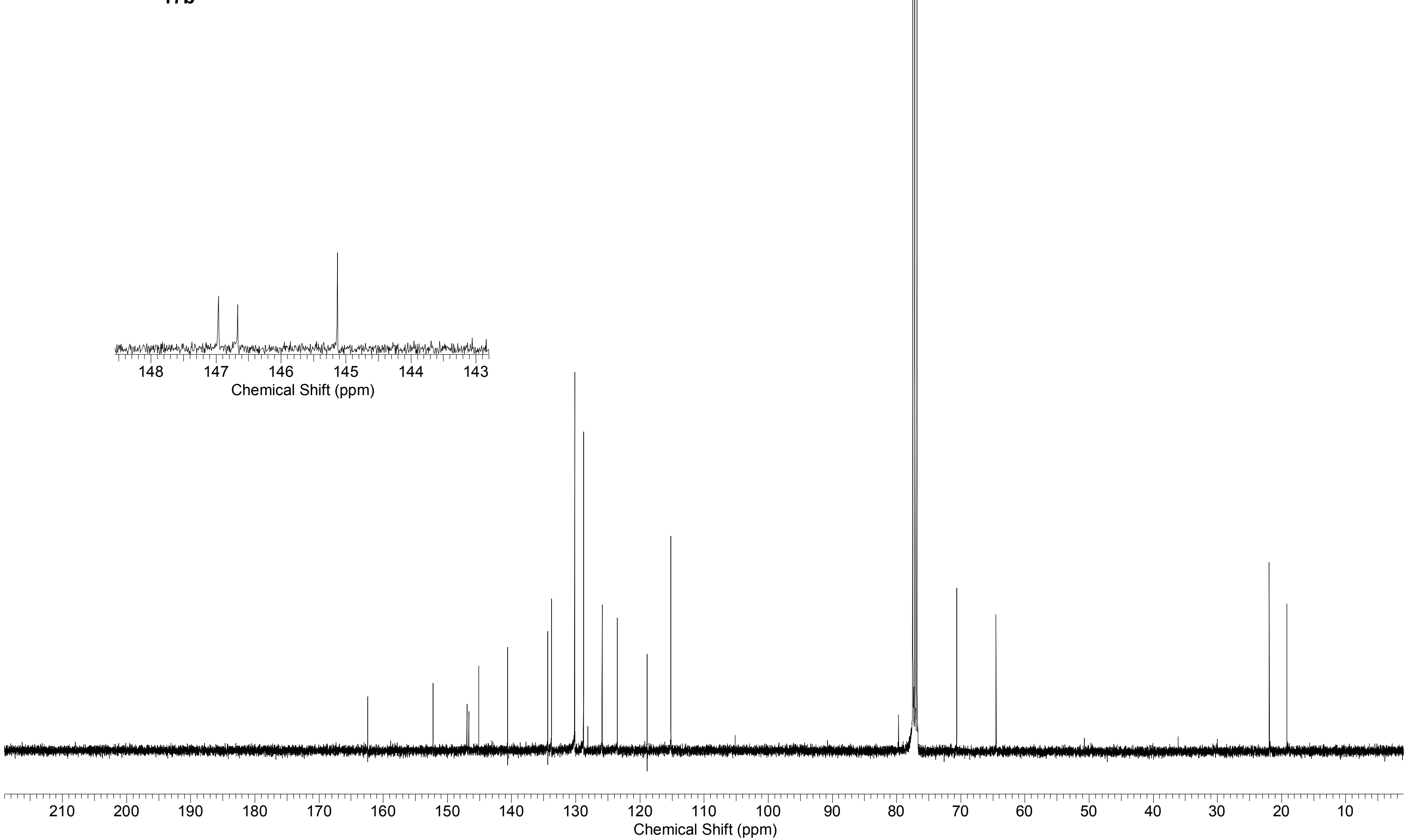
148 147 146 145 144 143
Chemical Shift (ppm)
210 200 190 180 170 160 150 140 130 120 110 100 90 80 70 60 50 40 30 20 10
Chemical Shift (ppm)

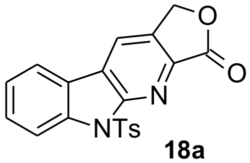
O
O
N
NTs

18a

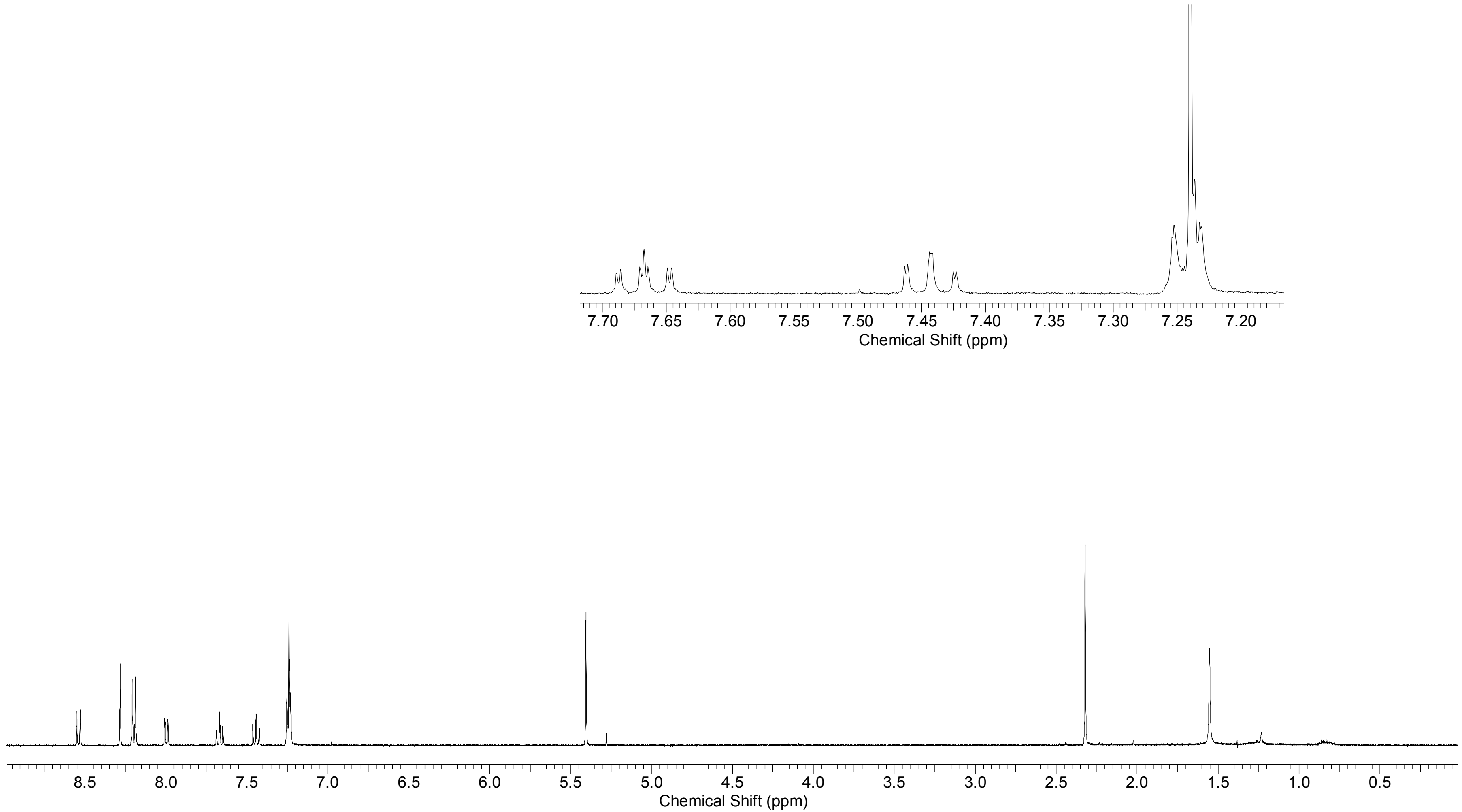
7.70
7.65
7.60
7.55
7.50
7.45
7.40
7.35
7.30
7.25
7.20
Chemical Shift (ppm)
8.5
8.0
7.5
7.0
6.5
6.0
5.5
5.0
4.5
4.0
3.5
3.0
2.5
2.0
1.5
1.0
0.5
Chemical Shift (ppm)

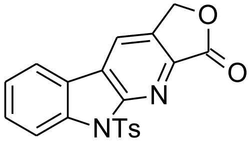
O
O
N
NTs

18a

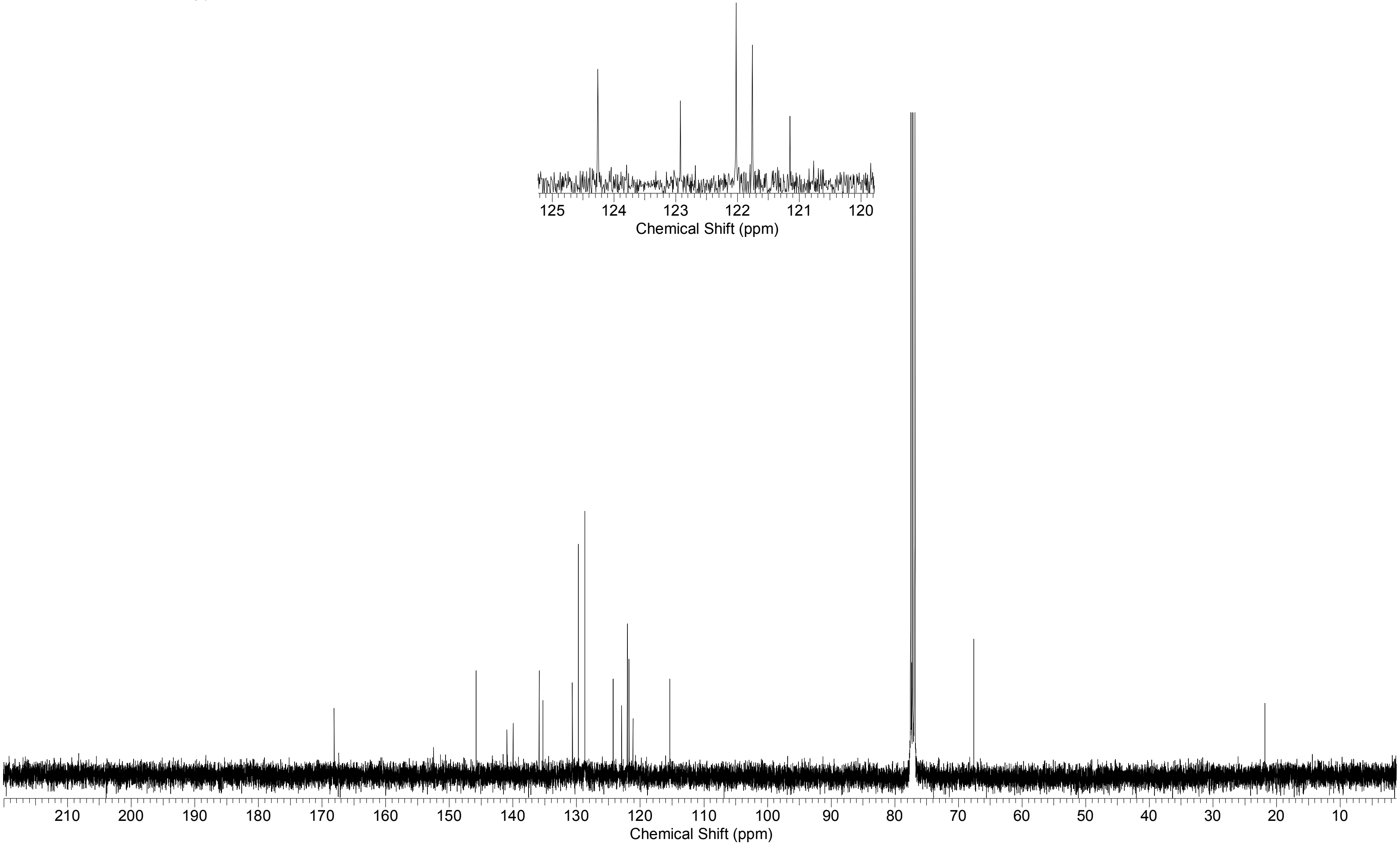
125 124 123 122 121 120
Chemical Shift (ppm)
210 200 190 180 170 160 150 140 130 120 110 100 90 80 70 60 50 40 30 20 10
Chemical Shift (ppm)
